# Synthesis of Spiroindenyl-2-Oxindoles through Palladium-Catalyzed Spirocyclization of 2-Bromoarylamides and Vinyl Bromides

**DOI:** 10.3390/molecules26247496

**Published:** 2021-12-10

**Authors:** Shuai Yang, Yanghui Zhang

**Affiliations:** Shanghai Key Laboratory of Chemical Assessment and Sustainability, School of Chemical Science and Engineering, Tongji University, 1239 Siping Road, Shanghai 200092, China; ys1987ywj@163.com

**Keywords:** C–H functionalization, palladium catalysis, spiroindenyl-2-oxindoles, palladacycles, cascade reaction

## Abstract

An expeditious approach to the construction of spiroindenyl-2-oxindoles was developed via a palladium-catalyzed spirocyclization reaction of 2-bromoarylamides with vinyl bromides. The reaction formed spiropalladacycles as the intermediates via carbopalladation and the C–H functionalization of 2-bromoarylamides. The spiropalladacycles reacted with vinyl bromides to form spiroindenyl-2-oxindoles. A Heck process rather than vinylic C–H functionalization was involved in the reaction.

## 1. Introduction

**Figure 1:**
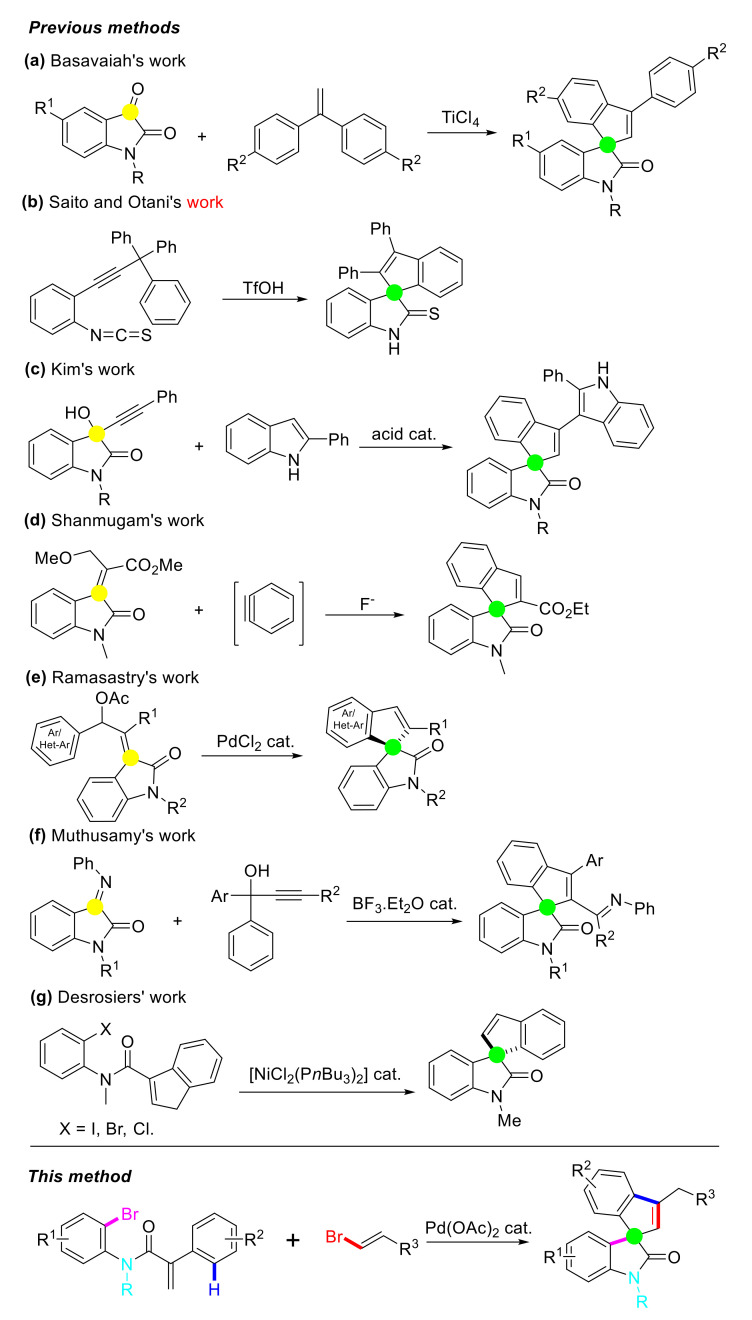
Synthetic methods of spiroindenyl-2-oxindoles.

Spirooxindoles are ubiquitous in bioactive natural products and have found extensive applications in drug discovery [1,2,3,4,5,6,7]. On the other hand, spiroindenes have also gained considerable interest in medicinal chemistry [8,9,10,11,12,13]. The investigation of biological and pharmaceutical properties of spiroindenyl-2-oxindoles that contain both spiro-bridged indene and oxindole moieties is highly intriguing [14,15]. To achieve this, it is essential to develop efficient synthetic methods for the analysis of compounds of such a type. Currently, the reactions for the synthesis of spiroindenyl-2-oxindoles are rare, and the majority of them rely on the use of 3-substituted indoles as the starting materials (Figure 1a–f) [14,15,16,17,18,19]. It should be noted that Desrosiers and coworkers reported an elegant synthetic approach for spiroindenyl-2-oxindoles through nickel-catalyzed intramolecular Heck cyclization (Figure 1g) [20,21].

Over the past few years, domino Heck/C–H functionalization reactions have gained considerable interest and made noticeable progress in organic synthesis [22,23]. By using alkene-tethered aryl halides as substrates, the reactions of such a type undergo the oxidative addition of aryl halides to Pd^0^ and intramolecular carbopalladation of alkene moieties to form alkylpalladium^II^ species. The alkylpalladium^II^ species can cleave C–H bonds of an aryl group tethered to the alkene moiety to generate C,C-palladacycles. The palladacycles may undergo intramolecular cyclization [24,25,26,27,28,29,30,31,32,33,34,35] or be captured with external reagents [36,37,38,39,40,41,42,43,44,45,46,47,48,49,50,51,52,53,54,55]. These types of reactions not only represent a novel strategy to activate C–H bonds that are not in proximity to directing groups, but also provide easy access to complex polycyclic compounds. Notably, the reactions also open a new avenue for the synthesis of spirocyclic scaffolds. By the judicious design of alkene-tethered aryl halides substrates, spiropalladacycles can be formed by a domino Heck/C–H functionalization sequence. The resulting spiropalladacycles are very effective intermediates for the synthesis of spirocyclic compounds. During recent years, quite a few reactions of this type have been developed. Spiropalladacycles have undergone cyclization [30,31,32,33,34,35] and have been trapped by a variety of external reagents including diaziridinone [44], benzynes [45,46,47], carbenoids [48,49], alkynes [50,51], CH_2_Br_2_ [52], alkyl chlorides [53], and aryl iodides [54,55], affording various spirocyclic products.

## 2. Results and Discussion

### 2.1. Optimization of the Reaction Conditions

Herein, we report a new approach for the synthesis of spiroindenyl-2-oxindoles through domino Heck/C–H functionalization reactions. This work was inspired by the reaction of C(sp^2^), C(sp^3^)-palladacycle derived from *ortho*-iodomethoxybenzenes with vinyl bromides [56]. It should be mentioned that the reactions of 2-iodobiphenyls with vinyl bromides have also been reported [57,58]. The reactions also proceeded via C,C-palladacycle intermediates, which were captured by vinyl bromides to form 9-fluorenylidene products.

The research was commenced by investigating the reaction of model substrates acrylamide (**1a**) and 1-bromoprop-1-ene (**2a**) (Table 1). After an extensive condition survey, spiroindenyl-2-oxindoles **3aa** was generated in a yield of 74% under the reaction conditions shown in entry 1. The optimal yield was obtained by using 18-crown-6, which promoted the reaction perhaps by enhancing the solubility of K_2_CO_3_ in THF (entry 2). Ligand s-phos played a crucial role in the reaction since its absence led to a very low yield and other phosphine ligands gave lower yields (entries 3–7). K_2_CO_3_ was an essential base, and only a trace amount of the product was observed when other bases such as Na_2_CO_3_ and KOAc were used (entries 8 and 9). Although **3aa** was also formed when the reaction was carried out in other solvents, the yields were much lower (entries 10–12).

### 2.2. Substrate Scope for Acrylamides

**Scheme 1:**
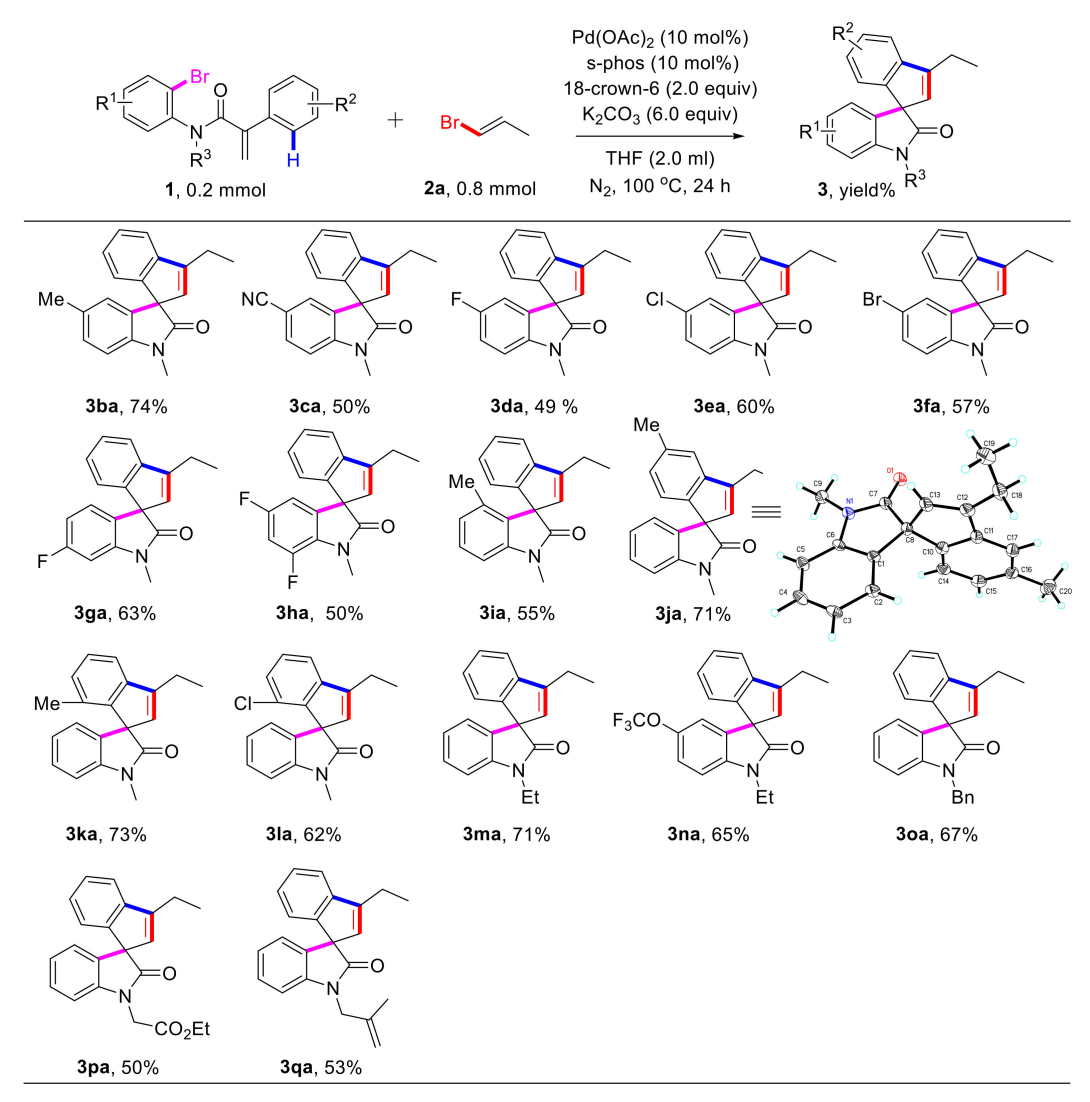
Acrylamide scope. All the reactions were carried out in a Schlenk tube in the presence of **1a** (0.2 mmol), **2a** (0.8 mmol), Pd(OAc)_2_ (10 mol%), s-phos (10 mol%), 18-crown-6 (2.0 equiv), K_2_CO_3_ (6.0 equiv), and THF (2.0 mL) at 100 °C for 24 h under N_2_.

Having developed an approach for the synthesis of spiroindenyl-2-oxindoles, we then probed its substrate scope (Scheme 1). We first examined the performance of acrylamides bearing different functionalities on the bromophenyl groups. The acrylamides containing an electron-donating methyl or electron-withdrawing cyano group underwent the cascade reaction (**3ba** and **3ca**). Fluoro, chloro, and even bromo groups were well tolerated, and the corresponding spiroindenyl-2-oxindoles were formed in moderate yields (**3da**–**3fa**). The substituents on the other positions of the bromophenyl groups were also suitable (**3ga**–**3ia**). Furthermore, substrates bearing a substituent on the phenyl groups linked to the double bonds could also be transformed into spiroindenyl-2-oxindole products (**3ja**–**3la**). The structure of **3ja** was confirmed by single crystal X-ray crystallography.

**Scheme 2:**
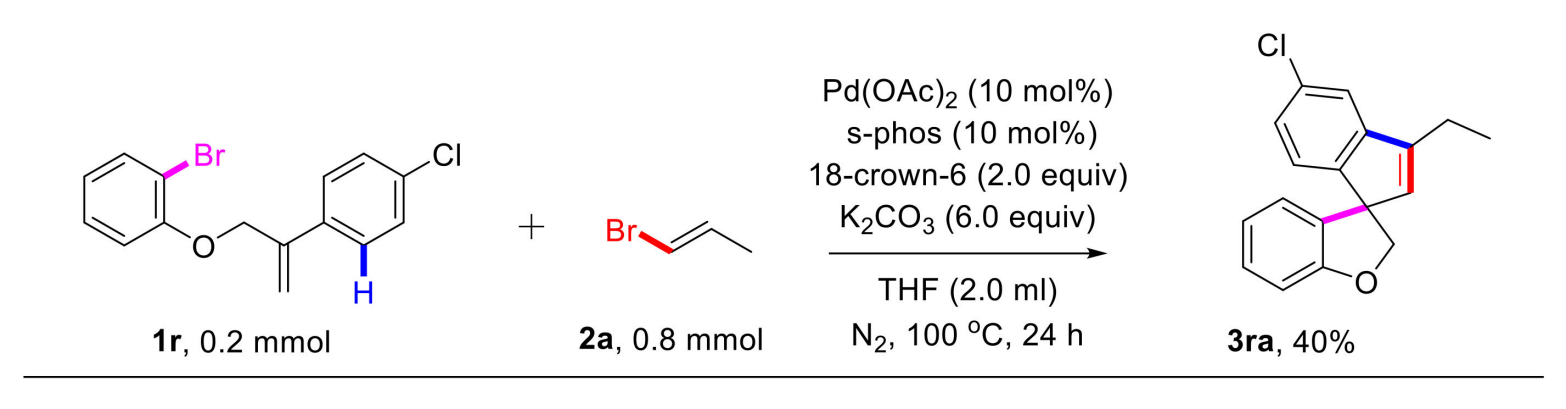
The reactions were carried out in a Schlenk tube in the presence of **1r** (0.2 mmol), **2a** (0.8 mmol), Pd(OAc)_2_ (10 mol%), s-phos (10 mol%), 18-crown-6 (2.0 equiv), K_2_CO_3_ (6.0 equiv), and THF (2.0 mL) at 100 °C for 24 h under N_2_.

Next, the reactions of acrylamide bearing different *N*-substituents were probed. A range of *N*-substituents, including the ethyl, benzyl 2-ethoxy-2-oxoethyl and 2-methylallyl group, were compatible, and a variety of spiroindenyl-2-oxindole derivatives were afforded (**3ma**–**3qa**). Finally, it should be noted that the substrate containing an ether linkage could also form the desired spirocyclic product **3ra** (Scheme 2).

### 2.3. Substrate Scope for Vinyl Bromides

**Scheme 3:**
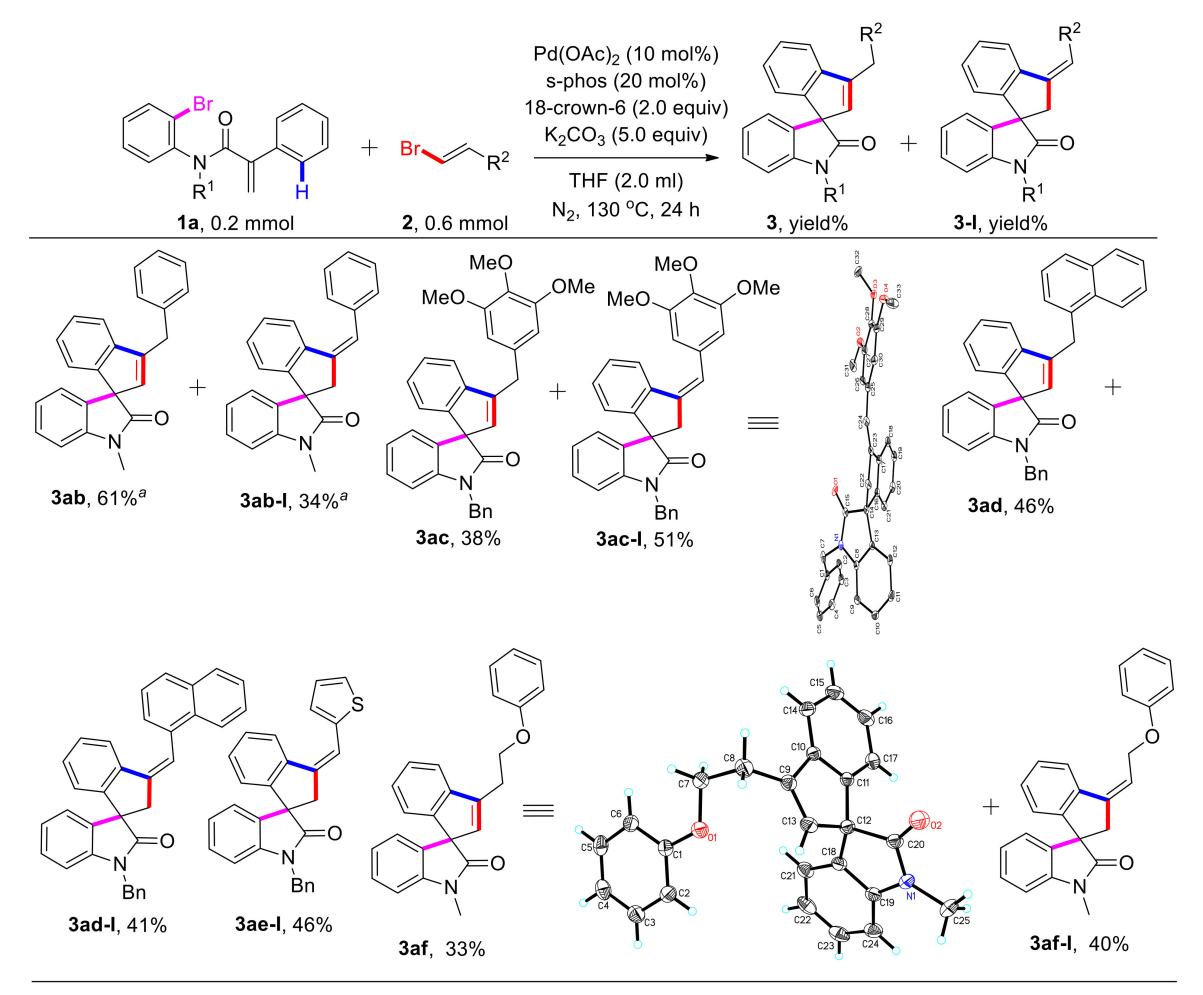
Vinyl bromide scope. All the reactions were carried out in a Schlenk tube in the presence of **1a** (0.2 mmol), **2** (0.6 mmol), Pd(OAc)_2_ (10 mol%), s-phos (20 mol%), 18-crown-6 (2.0 equiv), K_2_CO_3_ (5.0 equiv), and THF (2.0 mL) at 130 °C for 24 h under N_2_. *^a^* 0.4 mmol vinyl bromide.

The vinyl bromide scope was then explored (Scheme 3). When styryl bromide was allowed to react with **1a** under the slightly modified standard conditions, spiroindenyl-2-oxindole **3ab** and compound **3ab-I** were obtained. The formation of **3ab-I** should be due to the stabilization of the exocyclic double bond by the phenyl group. As expected, styryl bromide derivatives, such as trimethoxystyryl bromide and (*E*)-1-(2-bromovinyl)naphthalene, also gave two isomers (**3ac** and **3ac-I**). The structure of **3ac-I** was confirmed by single-crystal X-ray crystallography. It should be noted that the trimethoxyphenyl group was on the same side as the benzene ring, and the double bond in compound **3ac-I** had *Z*-configuration. This structure provides crucial evidence regarding the mechanism of the reaction. (*E*)-2-(2-bromovinyl)thiophene was also reactive, and only the exocyclic double bond product (**3ae-I**) was obtained. Intriguingly, two products (**3af** and **3af-I**) were also obtained in the reaction of alkylvinyl bromide **2f**. The structure of **3af** was also confirmed by single-crystal X-ray crystallography.

### 2.4. Mechanistic Studies

**Scheme 4:**
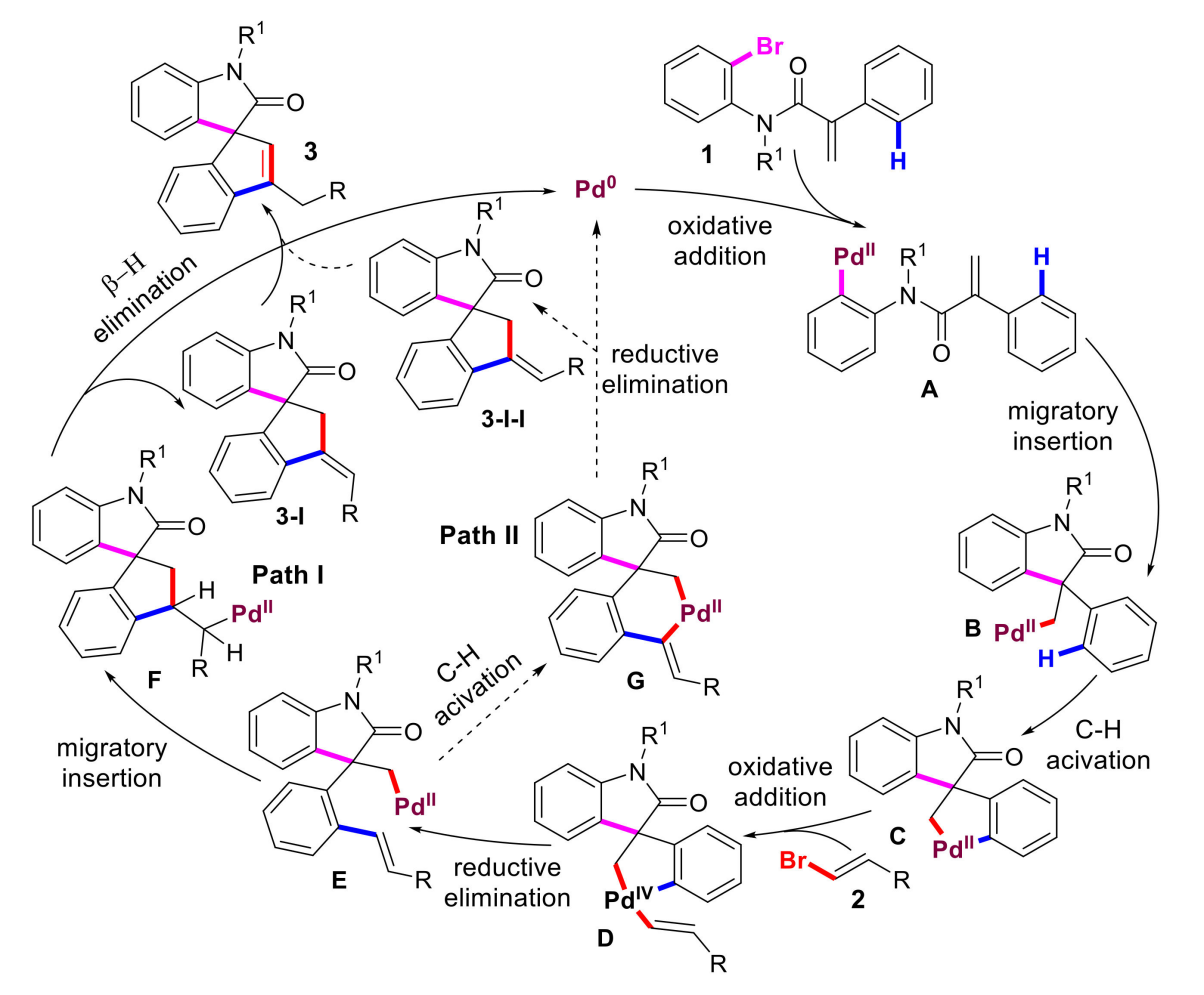
Proposed mechanism.

On the basis of the formation of the products and the previous reports [52,56,57,58], a tentative mechanism was proposed as shown in Scheme 4. The catalytic cycle starts with the oxidative addition of substrate 1 to Pd^0^ to form Pd^II^ species **A**, which is followed by intramolecular migratory insertion to give alkylPd^II^ species **B**. The subsequent intramolecular C–H functionalization affords palladacycle **C**. **C** undergoes oxidative addition with 2-bromoalkenyl derivatives to form Pd^IV^ species **D**. The reductive elimination of **D** yields intermediate **E**. At this stage, **E** may undergo two pathways to form final product **3**. Path **I** involves intermediate **3-I**, which is formed through intramolecular migratory insertion and subsequent β-H elimination. Alternatively, the alkylPd^II^ species of **E** may cleave the vinyl C–H bond to yield palladacycle **G**. **G** forms product **3-I-I** and Pd^0^ by reductive elimination (path **II**). Both **3-I** and **3-I-I** can isomerize to yield final product **3**. It is challenging to distinguish these two pathways. Fortunately, the mechanism can be deciphered based on the structure of intermediate **3ac-I**. If the reaction undergoes path **II**, **3-I-I**, which has *E*-configuration, should be formed. On the contrary, compound **3-I** with *Z*-configuration should be generated for **path I**. The *Z*-configuration of **3ac-I** indicates that the reaction proceeds via path **I**.

## 3. Materials and Methods

### 3.1. General Information

Pd(OAc)_2_ was purchased from Strem Chemicals (Newburyport, MA, USA). The 1H NMR and ^13^C NMR spectra were recorded on a Bruker ARX400 instrument (400 MHz) or a Bruker DRX-600 instrument (600 MHz). High-resolution mass spectra were measured on a Bruker MicroTOF II ESI-TOF mass spectrometer. NMR spectra were recorded in CDCl_3_. The 1H NMR spectra were referenced to residual CHCl_3_ at 7.26 ppm, and ^13^C NMR spectra were referenced to the central peak of CDCl_3_ at 77.0 ppm. Chemical shifts (δ) are reported in ppm and coupling constants (J) are in Hertz (Hz). Multiplicities are reported using the following abbreviations: s = singlet, d = doublet, t = triplet, q = quartet, and m = multiplet.

### 3.2. Experimental Procedures

Synthesis of Spiroindenyl-2-Oxindoles (Please see Supplementary Materials).

(a) A 25 mL Schlenk-type tube (with a Teflon screw cap and a side arm) equipped with a magnetic stir bar was charged with Pd(OAc)_2_ (0.02 mmol, 4.4 mg, 0.1 equiv), s-phos (0.02 mmol, 8.2 mg, 0.1 equiv), K_2_CO_3_ (1.2 mmol, 165.9 mg, 6.0 equiv), 18-crown-6 (0.4 mmol, 105.7 mg, 2.0 equiv), acrylamide **1a** (0.2 mmol, 63.2 mg, 1.0 equiv), 1-bromo-1-propene **2a** (0.8 mmol, 96.8 mg, 4.0 equiv), and THF (2.0 mL). The reaction mixture was frozen with liquid nitrogen, and then, the tube was evacuated and backfilled with nitrogen (6 times). The reaction tube was put into an oil bath and then heated to 100 °C. The reaction mixture was stirred at 100 °C for 24 h. After being cooled down to room temperature, the reaction mixture was diluted with EtOAc (15 mL), washed with brine (3 times), dried over Na_2_SO_4_, and concentrated in vacuo. The residue was purified by preparative silica gel TLC with petroleum ether/ethyl acetate (ether/ethyl acetate 25:1) to afford **3aa** (71%, 39.0 mg).
(b) A 25 mL Schlenk-type tube (with a Teflon screw cap and a side arm) equipped with a magnetic stir bar was charged with Pd(OAc)_2_ (0.02 mmol, 4.4 mg, 0.1 equiv), s-phos (0.04 mmol, 16.4 mg, 0.2 equiv) K_2_CO_3_ (1.0 mmol, 138.2 mg, 5.0 equiv), 18-crown-6 (0.4 mmol, 105.7 mg, 2.0 equiv), acrylamide **1a** (0.2 mmol, 63.2 mg, 1.0 equiv), β-bromostyrene **2b** (0.6 mmol, 109.8 mg, 3.0 equiv), and THF (2.0 mL). The reaction mixture was frozen with liquid nitrogen and then the tube was evacuated and backfilled with nitrogen (6 times). The reaction tube was put into an oil bath and then heated to 130 °C. The reaction mixture was stirred at 130 °C for 24 h. After being cooled down to room temperature, the reaction mixture was diluted with EtOAc (15 mL), washed with brine (3 times), dried over Na_2_SO_4_, and concentrated in vacuo. The residue was purified by preparative silica gel TLC with petroleum ether/ethyl acetate (ether/ethyl acetate 25:1) to afford **3ab** (61%, 41.2 mg) and **3ab-l** (34%, 22.9 mg).

## 4. Conclusions

In summary, we developed a palladium-catalyzed spirocyclization reaction between 2-bromoarylamides and vinyl bromides via a cascade Heck/C–H functionalization process. The reaction forms spiropalladacycles as the intermediate by carbopalladation and C–H functionalization of 2-bromoarylamides. The resulting spiropalladacycles react with vinyl bromides effectively and spiroindenyl-2-oxindoles are formed as the final products. The Z-configuration of the precursor was identified, and it indicates that a Heck process instead of a vinylic C–H functionalization is involved in the reaction. This reaction provides a novel and effective strategy for the construction of spiroindenyl-2-oxindoles.

## Figures and Tables

**Table 1:** Optimization of the reaction conditions.

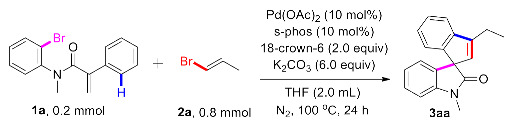		
Entry	Variation from the Standard Conditions	Yield *^a^*
1	No	74% (71% *^b^*)
2	No 18-crown-6	55%
3	No s-phos	18%
4	x-phos instead of s-phos	53%
5	Ru-phos instead of s-phos	62%
6	(o-tolyl)_3_P instead of s-phos	60%
7	Ph_3_P instead of s-phos	63%
8	Na_2_CO_3_ instead of K_2_CO_3_	5%
9	KOAc instead of K_2_CO_3_	3%
10	DMF instead of THF	43%
11	toluene instead of THF	59%
12	CH_3_CN instead of THF	14%

*^a^* The yields were determined by ^1^H NMR analysis of crude reaction mixtures by using CH_2_Br_2_ as the internal standard. *^b^* Isolated yield.

## Data Availability

The data presented in this study are available on request from the corresponding author.

## Supplementary Materials

The following are available online. Synthetic procedure of starting materials, procedure and spectral data of products, copies of ^1^H-NMR, ^13^C-NMR spectra.

## Sample Availability

Samples of the compounds are not available from the authors.
