# Supplementary material for: Synthesis of Spiroindenyl-2-Oxindoles through Palladium-Catalyzed Spirocyclization of 2-Bromoarylamides and Vinyl Bromides
Source: Molecules. 2021 Dec 10;26(24):7496. doi: 10.3390/molecules26247496 (PMC8704520; doi:10.3390/molecules26247496)
Supplement: Supplementary file 1 [file molecules-26-07496-s001.zip › molecules-1501486-supplementary.pdf]

Supporting Information

Synthesis of Spiroindenyl-2-oxindoles through Palladium-Catalyzed Spirocyclization of 2-Bromoarylamides and Vinyl Bromides

Shuai Yang, Yanghui Zhang*

School of Chemical Science and Engineering, Shanghai Key Laboratory of Chemical Assessment and Sustainability, Tongji University 1239 Siping Road, Shanghai 200092, China.
E-mail: zhangyanghui@tongji.edu.cn

Table of Contents

1. **General Information..S2**
2. **General Procedures for the Synthesis of Spiroindenyl-2-Oxindoles............S2**
3. **Characterization Data for the Products……………….................................S3**
4. **References...S14**
5. **Scanned ^{1}H NMR and ^{13}C NMR Spectra of the Products…….....................S15**
6. **X-Ray crystal structure of 3ja (CCDC: 2119724) …….........................S42**
7. **X-Ray crystal structure of 3ac-l (CCDC: 2119735)..................................S43**
8. **X-Ray crystal structure of 3af (CCDC: 2119736) S43**

1. General Information

$Pd(OAc)_2$ was purchased from Strem Chemicals. ^{1}H NMR and ^{13}C NMR spectra were recorded on Bruker ARX400 instrument (400 MHz) or Bruker DRX-600 instrument (600 MHz). High resolution mass spectra were measured on Bruker MicroTOF II ESI-TOF mass spectrometer. NMR spectra were recorded in $CDCl_3$. ^{1}H NMR spectra were referenced to residual $CHCl_3$ at 7.26 ppm, and ^{13}C NMR spectra were referenced to the central peak of $CDCl_3$ at 77.0 ppm. Chemical shifts (δ) are reported in ppm, and coupling constants (J) are in Hertz (Hz). Multiplicities are reported using the following abbreviations: s = singlet, d = doublet, t = triplet, q = quartet, m = multiplet.

Acrylamide and styryl bromides were synthesized by following the reported procedures.[1,2]

2. General Procedures for the Synthesis of Spiroindenyl-2-Oxindoles

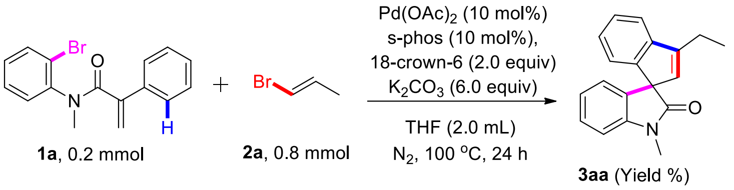

A 25 mL Schlenk-type tube (with a Teflon screw cap and a side arm) equipped with a magnetic stir bar was charged with $Pd(OAc)_2$ (0.02 mmol, 4.4 mg, 0.1 equiv), s-phos (0.02 mmol, 8.2 mg, 0.1 equiv), K_2CO_3 (1.2 mmol, 165.9 mg, 6.0 equiv), 18-crown-6 (0.4 mmol, 105.7 mg, 2.0 equiv), acrylamide **1a** (0.2 mmol, 63.2 mg, 1.0 equiv), 1-bromo-1-propene **2a** (0.8 mmol, 96.8 mg, 4.0 equiv) and THF (2.0 mL). The reaction mixture was frozen with liquid nitrogen and then the tube was evacuated and backfilled with nitrogen (6 times). The reaction tube was put into an oil bath and then heated to 100 °C. The reaction mixture was stirred at 100 °C for 24 hours. After being cooled down to room temperature, the reaction mixture was diluted with EtOAc (15 mL), washed with brine (3 times), dried over Na_2SO_4 and concentrated in vacuo. The residue was purified by preparative silica gel TLC with petroleum ether/ethyl acetate (ether/ethyl acetate 25:1) to afford **3aa** (71%, 39.0 mg).

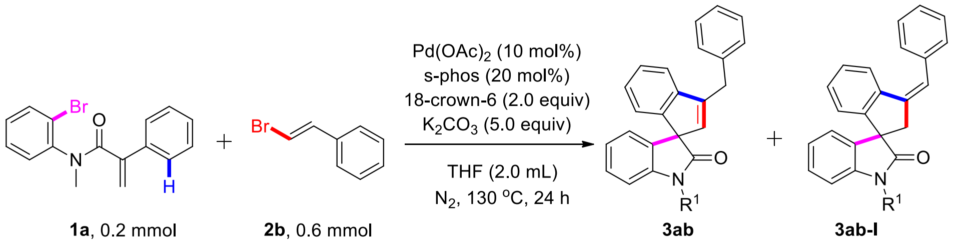

A 25 mL Schlenk-type tube (with a Teflon screw cap and a side arm) equipped with a magnetic stir bar was charged with $Pd(OAc)_2$ (0.02 mmol, 4.4 mg, 0.1 equiv), s-phos (0.04 mmol, 16.4 mg, 0.2 equiv) K_2CO_3 (1.0 mmol, 138.2 mg, 5.0 equiv), 18-crown-6 (0.4 mmol, 105.7 mg, 2.0 equiv), acrylamide **1a** (0.2 mmol, 63.2 mg, 1.0 equiv), β-bromostyrene **2b** (0.6 mmol, 109.8 mg, 3.0 equiv) and THF (2.0 mL). The reaction mixture was frozen with liquid nitrogen and then the tube was evacuated and backfilled with nitrogen (6 times). The reaction tube was put into an oil bath and then heated to 130 °C. The reaction mixture was stirred at 130 °C for 24 hours. After being cooled down to room temperature, the reaction mixture was diluted with EtOAc (15 mL), washed with brine (3 times), dried over Na_2SO_4 and concentrated in vacuo. The residue was purified by preparative silica gel TLC with petroleum ether/ethyl acetate (ether/ethyl acetate 25:1) to afford **3ab** (61%, 41.2 mg) and **3ab-l** (34%, 22.9 mg**).**

3. Characterization Data for the Products

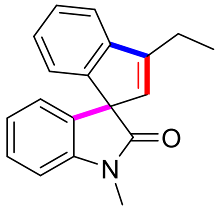

3aa

3-ethyl-1'-methylspiro[indene-1,3'-indolin]-2'-one: White solid, isolated yield 74% (40.8 mg) (eluent: petroleum ether/EtOAc = 25:1); ^{1}**H NMR** (400 MHz, Chloroform-*d*) δ 7.40 (d, *J* =7.2 Hz, 1H), 7.36 – 7.31 (m, 2H), 7.14 (td, *J* = 7.4, 1.0 Hz, 1H), 7.00 – 6.96 (m, 2H), 6.91 (d, *J* = 7.6 Hz, 1H), 6.77 (dd, *J* = 7.4, 1.0 Hz, 1H), 5.95 (t, *J* = 1.6 Hz, 1H), 3.35 (s, 3H), 2.77 – 2.62 (m, 2H), 1.34 (t, *J* = 7.4 Hz, 3H). ^{13}**C NMR** (151 MHz, $CDCl_3$) δ 175.7, 150.4, 146.1, 145.7, 144.8, 128.9, 128.6, 128.5, 127.8, 126.2, 123.5, 122.8, 122.2, 119.8, 108.3, 64.5, 26.9, 20.9, 11.9. HRMS (ESI) m/z: $[M + Na]^+$ calcd for $C_{19}H_{17}NNaO$ 298.1202; found 298.1194.

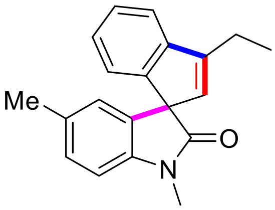

3ba

3-ethyl-1',5'-dimethylspiro[indene-1,3'-indolin]-2'-one Colorless viscous liquid, isolated yield 74% (42.8 mg) (eluent: petroleum ether/EtOAc = 25:1); ^{1}**H NMR** (600 MHz, Chloroform-*d*) δ 7.38 (d, *J* = 7.8 Hz, 1H), 7.32 (t, *J* = 7.5 Hz,1H), 7.14 – 7.10 (m, 2H), 6.91 (d, *J* = 7.2 Hz, 1H), 6.85 (d, *J* = 7.8 Hz, 1H), 6.58 (s, 1H), 5.93 (s, 1H), 3.31 (s, 3H), 2.74 – 2.62 (m, 2H), 2.23 (s, 3H), 1.33 (t, *J* = 7.5 Hz, 3H). ^{13}**C NMR** (151 MHz, CDCl3) δ 175.6, 150.3, 146.3, 145.8, 142.5, 132.4, 128.8, 128.7, 127.8, 126.2, 124.2, 122.3, 119.8, 108.0, 64.4, 27.0, 21.0, 20.9, 11.9. HRMS (ESI) m/z: $[M + Na]^+$ calcd for $C_{20}H_{19}NNaO$ 312.1359; found 312.1352.

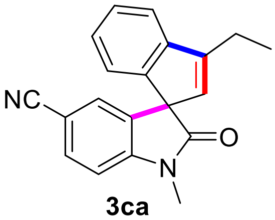

3ca

3-ethyl-1'-methyl-2'-oxospiro[indene-1,3'-indoline]-5'-carbonitrile White solid, isolated yield 50% (30.0 mg) (eluent: petroleum ether/EtOAc = 15:1); ^{1}**H NMR** (600 MHz, Chloroform-*d*) δ 7.64 (dd, *J* = 8.1, 1.5 Hz, 1H), 7.40 (d, *J* = 7.2 Hz, 1H), 7.36 (t, *J* = 7.5 Hz, 1H), 7.15 (t, *J* = 7.2 Hz, 1H), 7.03 – 7.00 (m, 2H), 6.85 (d, *J* = 7.2 Hz, 1H), 5.87 (s, 1H), 3.36 (s, 3H), 2.73 – 2.63 (m, 2H), 1.33 (t, *J* = 7.5 Hz, 3H). ^{13}**C NMR** (151 MHz, $CDCl_3$) δ 175.4, 151.8, 148.6, 145.6, 144.8, 133.7, 130.3, 128.4, 127.1, 126.8, 126.6, 122.1, 120.2, 118.9, 108.7, 106.1, 63.5, 27.2, 20.9, 11.8. HRMS (ESI) m/z: $[M + H]^+$ calcd for $C_{20}H_{17}N_2O$ 301.1335; 301.1336.

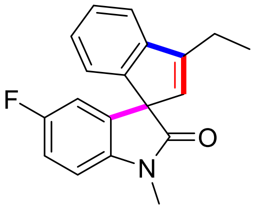

3da

3-ethyl-5'-fluoro-1'-methylspiro[indene-1,3'-indolin]-2'-one White solid, isolated yield 49% (28.8 mg) (eluent: petroleum ether/EtOAc = 25:1); **^{1}H NMR** (600 MHz, Chloroform-*d*) δ 7.37 (d, *J* = 7.2 Hz, 1H), 7.33 (t, *J* = 7.2 Hz, 1H), 7.13 (t, *J* = 6.9 Hz, 1H), 7.00 (td, *J* = 10.2, 2.6 Hz, 1H), 6.89 – 6.86 (m, 2H), 6.51 (dd, *J* = 7.8, 2.4 Hz, 1H), 5.90 (s, 1H), 3.31 (s, 3H), 2.73 – 2.61 (m, 2H), 1.32 (t, *J* = 7.2 Hz, 3H). **^{13}C NMR** (151 MHz, $CDCl_3$) δ 175.3, 159.4 (d, *J* = 241.2 Hz), 151.0, 145.6, 145.6, 140.7 (d, *J* = 1.8 Hz), 130.7 (d, *J* = 8.3 Hz), 128.1, 128.0, 126.4, 122.2, 120.0, 114.6 (d, *J* = 23.6 Hz), 111.5 (d, *J* = 24.9 Hz), 108.7 (d, *J* = 8.2 Hz), 64.4, 27.1, 20.9, 11.9. HRMS (ESI) m/z: $[M + H]^+$ calcd for $C_{19}H_{17}FNO$ 294.1289; found 294.1286.

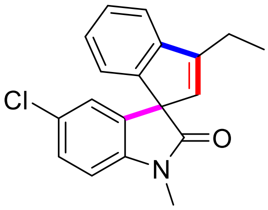

3ea

5'-chloro-3-ethyl-1'-methylspiro[indene-1,3'-indolin]-2'-one White solid, isolated yield 60% (37.2 mg) (eluent: petroleum ether/EtOAc = 25:1); **^{1}H NMR** (600 MHz, Chloroform-*d*) δ 7.38 (d, *J* = 7.2 Hz, 1H), 7.33 (td, *J* = 8.1, 1.2 Hz, 1H), 7.27 (dd, *J* = 8.4, 2.4 Hz, 1H), 7.13 (td, *J* = 7.8, 0.8 Hz, 1H), 6.88 (t, *J* = 7.5Hz, 2H), 6.72 (d, *J* = 2.4 Hz, 1H), 5.89 (t, *J* = 1.5 Hz, 1H), 3.31 (s, 3H), 2.72 – 2.61 (m, 2H), 1.32 (t, *J* = 7.5 Hz, 3H). **^{13}C NMR** (151 MHz, CDCl3) δ 175.2, 151.0, 145.6, 145.4, 143.3, 130.7, 128.4, 128.2, 128.1, 127.9, 126.4, 123.9, 122.2, 120.0, 109.2, 64.1, 27.1, 20.9,11.9. HRMS (ESI) m/z: $[M + Na]^+$ calcd for $C_{19}H_{16}ClNNaO$ 332.0813; found 332.0802.

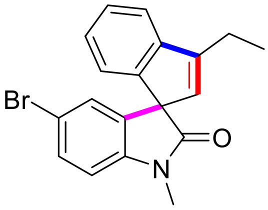

3fa

5'-bromo-3-ethyl-1'-methylspiro[indene-1,3'-indolin]-2'-one White solid, isolated yield 57% (40.4 mg) (eluent: petroleum ether/EtOAc = 25:1); **^{1}H NMR** (600 MHz, Chloroform-*d*) δ 7.43 (dd, *J* = 8.4, 1.8 Hz, 1H), 7.38 (d, *J* = 7.8 Hz, 1H), 7.33 (t, *J* = 7.5 Hz, 1H), 7.13 (t, *J* = 7.2 Hz, 1H), 6.88 (d, *J* = 7.8 Hz, 1H), 6.85 (d, *J* = 1.8 Hz, 1H), 6.83 (d, *J* = 8.4 Hz, 1H), 5.89 (t, *J* = 1.5 Hz, 1H), 3.30 (s, 3H), 2.72 – 2.61 (m, 2H), 1.32 (t, *J* = 7.2 Hz, 3H). **^{13}C NMR** (151 MHz, $CDCl_3$) δ 175.1, 151.0, 145.6, 145.4,

143.8, 131.3, 131.0, 128.1, 127.9, 126.6, 126.4, 122.2, 120.0, 115.4, 109.7, 64.0, 27.0, 20.9, 11.8. HRMS (ESI) m/z: [M + Na]$^+$ calcd for $C_{19}H_{16}BrNNaO$ 376.0307; found 376.0302.

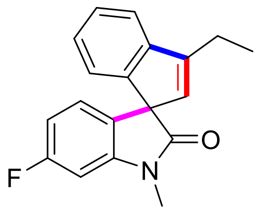

3ga

3-ethyl-6'-fluoro-1'-methylspiro[indene-1,3'-indolin]-2'-one White solid, isolated yield 63% (37.0 mg) (eluent: petroleum ether/EtOAc = 25:1); **^{1}H NMR** (600 MHz, Chloroform-*d*) δ 7.37 (d, *J* = 7.2 Hz, 1H), 7.32 (td, *J* = 8.1, 1.0 Hz, 1H), 7.12 (td, *J* = 8.1, 1.0 Hz, 1H), 6.88 (d, *J* = 7.2 Hz, 1H), 6.71 – 6.68 (m, 2H), 6.65 – 6.62 (m, 1H), 5.89 (t, *J* = 1.5Hz, 1H), 3.30 (s, 3H), 2.72 – 2.61 (m, 2H), 1.32 (t, *J* = 7.2 Hz, 3H). **^{13}C NMR** (151 MHz, $CDCl_3$) δ 176.0, 163.3 (d, *J* = 245.2 Hz), 150.6, 146.2 (d, *J* = 11.6 Hz), 145.9, 145.6, 128.3, 128.0, 126.3, 124.5 (d, *J* = 9.7 Hz), 123.8 (d, *J* = 2.9 Hz), 122.1, 119.9, 108.9 (d, *J* = 22.5 Hz), 97.2 (d, *J* = 27.6 Hz), 63.7, 27.1, 20.9, 11.9. HRMS (ESI) m/z: [M + Na]$^+$ calcd for $C_{19}H_{16}FNNaO$ 316.1108; found 316.1102.

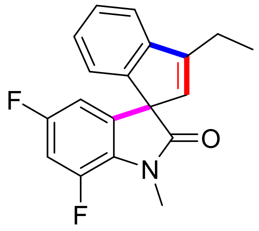

3ha

3-ethyl-5',7'-difluoro-1'-methylspiro[indene-1,3'-indolin]-2'-one White solid, isolated yield 50% (33.1 mg) (eluent: petroleum ether/EtOAc = 25:1); **^{1}H NMR** (600 MHz, Chloroform-*d*) δ 7.37 (d, *J* = 7.8 Hz, 1H), 7.33 (t, *J* = 7.5 Hz, 1H), 7.14 (t, *J* = 7.5 Hz, 1H), 6.91 (d, *J* = 7.2 Hz, 1H), 6.81 – 6.78 (m, 1H), 6.31 (dd, *J* = 7.2, 2.4 Hz, 1H), 5.88 (s, 1H), 3.51 (d, *J* = 2.4 Hz, 3H), 2.72 – 2.61 (m, 2H), 1.32 (t, *J* = 7.2 Hz, 3H). **^{13}C NMR** (151 MHz, $CDCl_3$) δ 174.9, 158.4 (dd, *J* = 244.6, 10.0 Hz), 151.3, 146.9 (dd, *J* = 246.7, 11.9 Hz), 145.5, 145.3, 132.8 (dd, *J* = 9.4, 4.3 Hz), 128.3, 127.6, 127.5 (dd, *J* = 8.8, 3.2 Hz), 126.6, 122.1, 120.0, 107.3 (dd, *J* = 24.5, 3.8 Hz), 104.4 (dd, *J* = 27.0, 23.4 Hz), 64.5, 29.3 (d, *J* =5.4Hz), 20.9, 11.8. HRMS (ESI) m/z: [M + H]$^+$ calcd for $C_{19}H_{16}F_2NO$ 312.1194 ; 312.1192

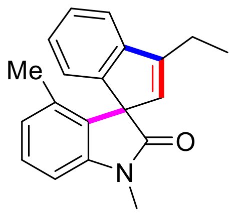

3ia

3-ethyl-1',4'-dimethylspiro[indene-1,3'-indolin]-2'-one White solid, isolated yield 55% (31.8 mg) (eluent: petroleum ether/EtOAc = 25:1); **^{1}H NMR** (600 MHz, Chloroform-*d*) δ 7.39 (d, *J* = 7.2 Hz, 1H),

7.32 (td, J = 8.1, 1.0 Hz, 1H), 7.22 (t, J = 7.8 Hz, 1H), 7.12 (t, J = 7.8 Hz, 1H), 6.91 (d, J = 7.2 Hz, 1H), 6.80 (d, J = 7.8 Hz, 1H), 6.76 (d, J = 7.8 Hz, 1H), 5.86 (t, J = 1.5 Hz, 1H), 3.30 (s, 3H), 2.75 – 2.62 (m, 2H), 1.57 (s, 3H), 1.32 (t, J = 7.5 Hz, 3H). **^{13}C NMR** (151 MHz, $CDCl_3$) δ 175.4, 150.7, 146.0, 144.9, 144.3, 135.4, 128.2, 127.7, 127.0, 126.2, 126.2, 124.9, 122.2, 119.9, 105.7, 64.4, 27.0, 20.9, 16.2, 12.0. HRMS (ESI) m/z: $[M + Na]^+$ calcd for $C_{20}H_{19}NNaO$ 312.1359; 312.1363

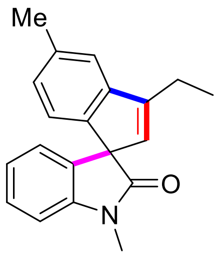

3ja

3-ethyl-1',5-dimethylspiro[indene-1,3'-indolin]-2'-one White solid, isolated yield 71% (41.1 mg) (eluent: petroleum ether/EtOAc = 25:1); **^{1}H NMR** (600 MHz, Chloroform-*d*) δ 7.30 (td, J = 7.8, 1.2 Hz, 1H), 7.19 (s, 1H), 6.96 – 6.93 (m, 3H), 6.78 (d, J = 7.8 Hz, 1H), 6.76 (d, J = 7.8 Hz, 1H), 5.90 (s, 1H), 3.32 (s, 3H), 2.71 – 2.60 (m, 2H), 2.39 (s, 3H), 1.32 (t, J = 7.2 Hz, 3H). **^{13}C NMR** (151 MHz, $CDCl_3$) δ 175.9, 150.3, 145.9, 144.8, 143.3, 137.6, 129.1, 128.9, 128.4, 126.9, 123.4, 122.8, 121.9, 120.6, 108.3, 63.9, 26.9, 21.6, 20.9, 12.0. HRMS (ESI) m/z: $[M + Na]^+$ calcd for $C_{20}H_{19}NNaO$ 312.1359; found 312.1361.

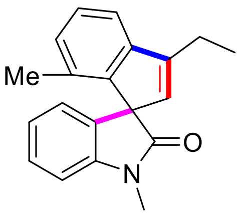

3ka

3-ethyl-1',7-dimethylspiro[indene-1,3'-indolin]-2'-one White solid, isolated yield 73% (42.3 mg) (eluent: petroleum ether/EtOAc = 25:1); **^{1}H NMR** (600 MHz, Chloroform-*d*) δ 7.30 (td, J = 7.8, 1.2 Hz, 1H), 7.24 (t, J = 7.5 Hz, 1H), 7.21 (d, J = 7.2 Hz, 1H), 6.96 – 6.93 (m, 2H), 6.91 (d, J = 7.2 Hz, 1H), 6.73 (d, J = 7.8 Hz, 1H), 5.85 (t, J = 1.5 Hz, 1H), 3.35 (s, 3H), 2.68 – 2.57 (m, 2H), 1.72 (s, 3H), 1.28 (t, J = 7.2 Hz, 3H). **^{13}C NMR** (151 MHz, $CDCl_3$) δ 175.0, 149.6, 146.4, 144.8, 143.7, 133.1 129.1, 128.4, 128.3, 128.0, 127.2, 123.3, 122.8, 117.4, 108.2, 64.1, 26.8, 20.9, 17.2, 11.9. HRMS (ESI) m/z: $[M + H]^+$ calcd for $C_{20}H_{20}NO$ 290.1539; found 290.1532.

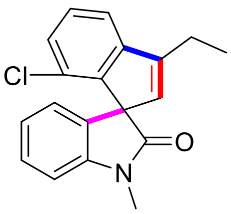

3la

7-chloro-3-ethyl-1'-methylspiro[indene-1,3'-indolin]-2'-one White solid, isolated yield 62% (38.4 mg) (eluent: petroleum ether/EtOAc = 25:1); **^{1}H NMR** (600 MHz, Chloroform-*d*) δ 7.29 (td, *J* = 8.1, 0.8 Hz, 1H), 7.25 – 7.22 (m, 2H), 7.05 (dd, *J* = 7.2, 1.2 Hz, 1H), 6.94 – 6.90 (m, 2H), 6.69 (d, *J* = 7.2 Hz, 1H), 5.89 (s, 1H), 3.31 (s, 3H), 2.64 – 2.53 (m, 2H), 1.25 (t, *J* = 7.2 Hz, 3H). **^{13}C NMR** (151 MHz, $CDCl_3$) δ 173.7, 149.1, 148.4, 145.4, 142.6, 130.2, 129.8, 129.5, 128.6, 126.7, 125.9, 123.1, 122.7, 118.2, 108.3, 64.4, 27.0, 20.9, 11.8. HRMS (ESI) m/z: $[M + Na]^+$ calcd for $C_{19}H_{16}ClNNaO$ 332.0813; found 332.0809.

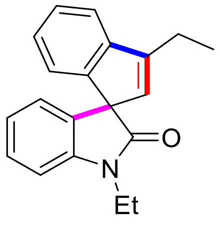

3ma

1',3-diethylspiro[indene-1,3'-indolin]-2'-one White solid, isolated yield 71% (41.1 mg) (eluent: petroleum ether/EtOAc = 25:1); **^{1}H NMR** (600 MHz, Chloroform-*d*) δ 7.36 (d, *J* = 7.8 Hz, 1H), 7.32 – 7.28 (m, 2H), 7.11 (td, *J* = 7.8, 0.8 Hz, 1H), 6.97 (d, *J* = 8.4 Hz, 1H), 6.93 (td, *J* = 8.1, 1.0 Hz, 1H), 6.87 (d, *J* = 7.8 Hz, 1H), 6.75 (d, *J* = 7.2 Hz, 1H), 5.93 (t, *J* = 1.5 Hz, 1H), 3.92 – 3.81 (m, 2H), 2.72 – 2.61 (m, 2H), 1.36 – 1.31 (m, 6H). **^{13}C NMR** (151 MHz, $CDCl_3$) δ 175.2, 150.5, 146.3, 145.7, 143.9, 129.1, 128.6, 128.4, 127.8, 126.2, 123.6, 122.6, 122.0, 119.8, 108.5, 64.3, 35.3, 20.9, 12.9, 12.0. HRMS (ESI) m/z: $[M + Na]^+$ calcd for $C_{20}H_{19}NNaO$ 312.1359; found 312.1340.

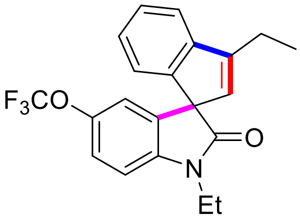

3na

1',3-diethyl-5'-(trifluoromethoxy)spiro[indene-1,3'-indolin]-2'-one White solid, isolated yield 65% (48.5 mg) (eluent: petroleum ether/EtOAc = 25:1); **^{1}H NMR** (600 MHz, Chloroform-*d*) δ 7.38 (d, *J* = 7.2 Hz, 1H), 7.34 (td, *J* = 7.8, 0.6 Hz, 1H), 7.17 (dd, *J* = 10.2,1.5 Hz, 1H), 7.13 (t, *J* = 7.5 Hz,1H), 6.95 (d, *J* = 8.4 Hz, 1H), 6.86 (d, *J* = 7.8 Hz, 1H), 6.64 (d, *J* = 1.2 Hz,1H), 5.91 (t, *J* = 1.5 Hz,1H), 3.92 – 3.81 (m, 2H), 2.73 – 2.62 (m, 2H), 1.36 – 1.32 (m, 6H). **^{13}C NMR** (151 MHz, $CDCl_3$) δ 174.9, 151.2, 145.6, 145.5, 144.7, 142.5, 130.9, 128.1, 127.7, 126.5, 120.4 (q, *J* = 256.7 Hz), 122.0, 121.4, 120.0, 117.6, 108.7, 64.2, 35.5, 20.9, 12.8, 11.9. HRMS (ESI) m/z: $[M + Na]^+$ calcd for $C_{21}H_{18}F_3NNaO_2$ 396.1182 ; 396.1185

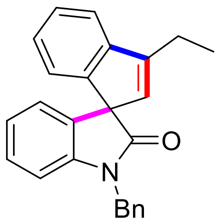

3oa

1'-benzyl-3-ethylspiro[indene-1,3'-indolin]-2'-one White solid, isolated yield 67% (47.1 mg) (eluent: petroleum ether/EtOAc = 25:1); **^{1}H NMR** (600 MHz, Chloroform-*d*) δ 7.39 – 7.32 (m, 6H), 7.29 (t, *J* = 7.2 Hz, 1H), 7.17 (td, *J* = 8.4, 1.2 Hz, 1H), 7.13 (t, *J* = 7.5 Hz, 1H), 6.91 – 6.89 (m, 2H), 6.83 (d, *J* = 7.8 Hz, 1H), 6.74 (d, *J* = 7.2 Hz, 1H), 5.98 (s, 1H), 5.11 (d, *J* = 15.6 Hz, 1H), 4.91 (d, *J* = 15.0 Hz, 1H), 2.74 – 2.63 (m, 2H), 1.34 (t, *J* = 7.5 Hz, 3H). **^{13}C NMR** (151 MHz, $CDCl_3$) δ 175.8, 150.6, 146.3, 145.7, 143.9, 136.1, 128.9, 128.9, 128.6, 128.4, 127.9, 127.7, 127.4, 126.3, 123.5, 122.8, 122.2, 119.9, 109.4, 64.2, 44.3, 21.0, 12.0. HRMS (ESI) m/z: $[M + Na]^+$ calcd for $C_{25}H_{21}NNaO$ 374.1515; found 374.1510

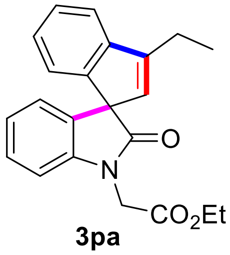

3pa

Ethyl -2-(3-ethyl-2'-oxospiro[indene-1,3'-indolin]-1'-yl)acetate Colorless viscous liquid, isolated yield 50% (34.7 mg) (eluent: petroleum ether/EtOAc = 15:1); **^{1}H NMR** (600 MHz, Chloroform-*d*) δ 7.37 (d, *J* = 7.8 Hz, 1H), 7.32 (td, *J* = 7.8, 0.8 Hz, 1H), 7.29 – 7.26 (m, 1H), 7.13 (t, *J* = 7.8, 0.8 Hz, 1H), 7.02 (d, *J* = 7.2 Hz, 1H), 6.96 (td, *J* = 7.8, 0.6 Hz, 1H), 6.83 (d, *J* = 7.8 Hz, 1H), 6.77 (dd, *J* = 7.2, 0.6 Hz, 1H), 5.96 (t, *J* = 1.5 Hz, 1H), 4.70 (d, *J* = 17.4 Hz, 1H), 4.44 (d, *J* = 17.4 Hz, 1H), 4.26 (q, *J* = 7.2 Hz, 2H), 2.73 – 2.62 (m, 2H), 1.34 –1.29 (m, 6H). **^{13}C NMR** (151 MHz, $CDCl_3$) δ 175.8, 167.6, 150.8, 146.3, 145.5, 143.5, 128.7, 128.4, 128.2, 127.9, 126.4, 123.6, 123.2, 122.5, 119.7, 108.29, 64.0, 61.9, 41.8, 20.9, 14.2, 11.9. HRMS (ESI) m/z: $[M + H]^+$ calcd for $C_{22}H_{22}NO_3$ 348.1594; found 348.1583.

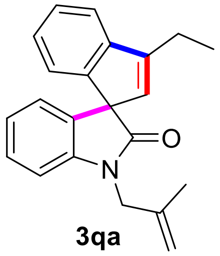

3qa

3-ethyl-1'-(2-methylallyl)spiro[indene-1,3'-indolin]-2'-one White solid, isolated yield 53% (33.4 mg) (eluent: petroleum ether/EtOAc = 25:1); **^{1}H NMR** (600 MHz, Chloroform-*d*) δ 7.38 (d, *J* = 7.2 Hz, 1H), 7.32 (t, *J* = 7.5 Hz, 1H), 7.27 – 7.24 (m, 1H), 7.12 (t, *J* = 7.2 Hz, 1H), 6.95 –6.92 (m, 2H), 6.90 (d, *J* = 7.8 Hz, 1H), 6.75 (d, *J* = 7.8 Hz, 1H), 5.93 (s, 1H), 5.00 (d, *J* = 7.8 Hz, 2H), 4.47 (d, *J* = 16.2 Hz, 1H),

4.28 (d, J = 16.2 Hz, 1H), 2.73 – 2.62 (m, 2H), 1.80 (s, 3H), 1.32 (t, J = 7.5 Hz, 3H). **^{13}C NMR** (151 MHz, $CDCl_3$) δ 175.5, 150.4, 146.3, 145.8, 144.2, 139.2, 128.7, 128.7, 128.3, 127.8, 126.3, 123.5, 122.7, 122.1, 119.8, 112.8, 109.4, 64.2, 46.4, 20.9, 20.0, 11.9. HRMS (ESI) m/z: $[M + H]^+$ calcd for $C_{22}H_{22}NO$ 316.1696; found 316.1694.

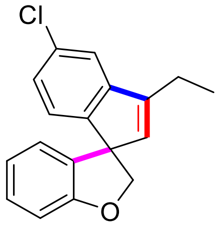

3ra

5'-chloro-3'-ethyl-2H-spiro[benzofuran-3,1'-indene] White solid, isolated yield 40% (22.6 mg) (eluent: petroleum ether/EtOAc = 50:1); **^{1}H NMR** (600 MHz, Chloroform-*d*) δ 7.27 – 7.26 (m, 1H), 7.17 (t, J = 7.8 Hz, 1H), 7.14 – 7.09 (m, 2H), 6.95 (d, J = 7.8 Hz, 1H), 6.77 (t, J = 7.8 Hz, 1H), 6.63 (d, J = 7.2 Hz, 1H), 6.22 (s, 1H), 4.69 (d, J = 9.0 Hz, 1H), 4.61 (d, J = 9.0 Hz, 1H), 2.57 – 2.52 (m, 2H), 1.39 (t, J = 7.5 Hz, 3H). **^{13}C NMR** (151 MHz, $CDCl_3$) δ 160.9, 149.4, 145.6, 145.2, 135.3, 133.4, 129.3, 128.8, 125.9, 123.6, 123.2, 121.0, 119.8, 110.3, 77.9, 61.1, 20.5, 12.1. HRMS (ESI) m/z: $[M + H]^+$ calcd for $C_{18}H_{16}ClO$ 283.0884; found 283.0880.

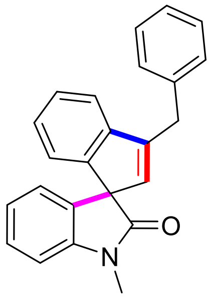

3ab

3-benzyl-1'-methylspiro[indene-1,3'-indolin]-2'-one Colorless viscous liquid, isolated yield 61% (41.2 mg) (eluent: petroleum ether/EtOAc = 25:1); **^{1}H NMR** (600 MHz, Chloroform-*d*) δ 7.35 (d, J = 7.2 Hz, 2H), 7.31 – 7.24 (m, 5H), 7.21 (t, J = 7.2 Hz, 1H), 7.10 (t, J = 7.5 Hz, 1H), 6.96 – 6.93 (m, 2H), 6.89 (d, J = 7.8 Hz, 1H), 6.76 (d, J = 7.8 Hz, 1H), 5.84 (s, 1H), 4.02 (d, J = 16.2 Hz, 1H), 3.97 (d, J = 16.2 Hz, 1H), 3.30 (s, 3H). **^{13}C NMR** (101 MHz, $CDCl_3$) δ 175.3, 147.5, 146.2, 145.3, 144.9, 138.3, 131.9, 129.0, 128.6, 128.5, 127.9, 126.4, 123.5, 122.9, 122.3, 120.3, 108.4, 64.4, 34.4, 26.9. HRMS (ESI) m/z: $[M + H]^+$ calcd for $C_{24}H_{20}NO$ 338.1539; found 338.1544.

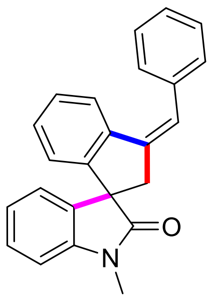

3ab-I

(Z)-3-benzylidene-1'-methyl-2,3-dihydrospiro[indene-1,3'-indolin]-2'-one Colorless viscous liquid, isolated yield 34% (22.9 mg) (eluent: petroleum ether/EtOAc = 25:1); ^{1}**H NMR** (400 MHz, Chloroform-*d*) δ 7.51 (d, *J* = 7.6 Hz, 2H), 7.42 (t, *J* = 7.6 Hz, 2H), 7.35 – 7.31 (m, 3H), 7.15 – 7.02 (m, 4H), 6.96 (d, *J* = 8.0 Hz, 1H), 6.77 – 6.75 (m, 2H), 3.55 (dd, *J* = 15.6, 2.0 Hz, 1H), 3.35 (s, 3H), 3.15 (dd, *J* = 15.2, 1.6 Hz, 1H). ^{13}**C NMR** (101 MHz, $CDCl_3$) δ 179.0, 148.3, 143.2, 140.1, 139.9, 137.7, 134.5, 129.0, 128.6, 128.5, 128.3, 127.6, 127.1, 124.7, 123.7, 123.3, 123.1, 123.0, 108.1, 57.6, 46.3, 26.6. HRMS (ESI) m/z: $[M + H]^+$ calcd for $C_{24}H_{20}NO$ 338.1539; found 338.1538.

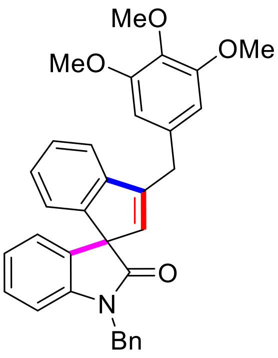

3ac

1'-benzyl-3-(3,4,5-trimethoxybenzyl)spiro[indene-1,3'-indolin]-2'-one Colorless viscous liquid, isolated yield 38% (38.3 mg) (eluent: petroleum ether/EtOAc = 25:1); ^{1}**H NMR** (400 MHz, Chloroform-*d*) δ 7.37 – 7.27 (m, 7H), 7.21 – 7.12 (m, 2H), 6.93 – 6.89 (m, 2H), 6.84 (d, *J* = 8.0 Hz, 1H), 6.77 (d, *J* = 7.2 Hz, 1H), 6.62 (s, 2H), 6.01 (s, 1H), 5.12 (d, *J* = 15.6 Hz, 1H), 4.90 (d, *J* = 15.6 Hz, 1H), 3.97 (s, 2H), 3.84 –3.83 (m, 9H). ^{13}**C NMR** (101 MHz, $CDCl_3$) δ 175.3, 153.2, 147.3, 146.4, 145.1, 144.0, 136.5, 136.0, 134.0, 132.1, 128.9, 128.5, 128.5, 127.9, 127.7, 127.4, 126.5, 123.4, 122.8, 122.2, 120.4, 109.5, 105.9, 64.4, 60.9, 56.1, 44.4, 34.5. HRMS (ESI) m/z: $[M + H]^+$ calcd for $C_{33}H_{30}NO_4$ 504.2169; found 504.2163.

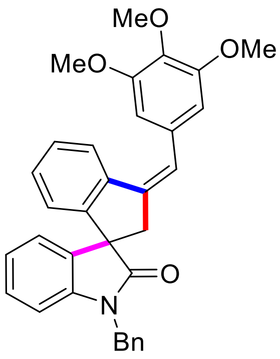

3ac-I

(Z)-1'-benzyl-3-(3,4,5-trimethoxybenzylidene)-2,3-dihydrospiro[indene-1,3'-indolin]-2'-one White solid, isolated yield 51% (51.4 mg) (eluent: petroleum ether/EtOAc = 10:1); **^{1}H NMR** (600 MHz, Chloroform-d) δ 7.48 (d, *J* = 7.8 Hz, 1H), 7.38 – 7.34 (m, 4H), 7.30 – 7.28 (m, 1H), 7.19 (t, *J* = 7.8 Hz, 1H), 7.12 – 7.07 (m, 3H), 7.00 (t, *J* = 7.5 Hz, 1H), 6.83 (d, *J* = 7.8 Hz, 1H), 6.74 – 6.73 (m, 3H), 6.70 (s, 1H), 5.09 (d, *J* = 15.6 Hz, 1H), 4.90 (d, *J* = 15.6 Hz, 1H), 3.92 (s, 3H), 3.86 (s, 6H), 3.55 (dd, *J* = 15.6, 1.8 Hz, 1H), 3.22 (dd, *J* = 15.6, 1.8 Hz, 1H). **^{13}C NMR** (151 MHz, CDCl3) δ 178.0, 152.2, 147.3, 141.5, 138.9, 138.9, 136.1, 135.0, 133.3, 132.1, 128.1, 127.8, 127.2, 126.7, 126.6, 126.3, 123.8, 122.8, 122.4, 122.1, 121.8, 108.2, 104.5, 60.0, 56.4, 55.2, 45.1, 43.0. HRMS (ESI) m/z: [M + H]+ calcd for C33H30NO4 504.2169; found 504.2156.

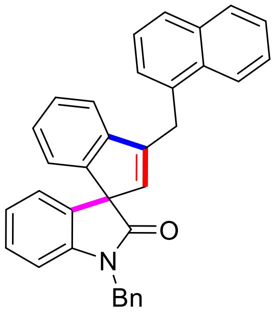

3ad

1'-benzyl-3-(naphthalen-1-ylmethyl)spiro[indene-1,3'-indolin]-2'-one Colorless viscous liquid, isolated yield 46% (42.7 mg) (eluent: petroleum ether/EtOAc = 25:1); **^{1}H NMR** (400 MHz, Chloroform-*d)* δ 8.12 (d, *J* = 7.6 Hz, 1H), 7.91 – 7.88 (m, 1H), 7.80 (d, *J* = 8.0 Hz, 1H), 7.57 – 7.44 (m, 5H), 7.38 – 7.28 (m, 6H), 7.22 – 7.14 (m, 2H), 6.97 (d, *J* = 7.6 Hz, 1H), 6.90 (t, *J* = 7.4 Hz, 1H), 6.81 (d, *J* = 8.0 Hz, 1H), 6.76 (d, *J* = 6.8 Hz, 1H), 5.78 (s, 1H), 5.12 (d, *J* = 15.6 Hz, 1H), 4.86 (d, *J* = 15.6 Hz, 1H), 4.54 – 4.43 (m, 2H). **^{13}C NMR** (101 MHz, $CDCl_3$) δ 175.3, 147.2, 146.4, 145.4, 143.9, 136.0, 134.3, 133.9, 132.3, 132.1, 128.8, 128.7, 128.5, 128.4, 128.0, 127.7, 127.4, 127.3, 127.1, 126.6, 126.0, 125.7, 125.6, 124.2, 123.6, 122.8, 122.3, 120.3, 109.4, 64.4, 44.3, 31.8. HRMS (ESI) m/z: $[M + H]^+$ calcd for $C_{34}H_{26}NO$ 464.2009; found 464.2014.

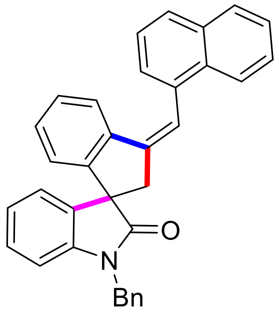

3ad-l

(Z)-1'-benzyl-3-(naphthalen-1-ylmethylene)-2,3-dihydrospiro[indene-1,3'-indolin]-2'-one White solid, isolated yield 41% (38.0 mg) (eluent: petroleum ether/EtOAc = 25:1); **^{1}H NMR** (600 MHz, Chloroform-*d*) δ 8.16 (d, *J* = 9.0 Hz, 1H), 7.92 – 7.91 (m, 1H), 7.87 (d, *J* = 8.4 Hz, 1H), 7.67 (d, *J* = 7.2 Hz, 1H), 7.53 – 7.49 (m, 3H), 7.39 – 7.35 (m, 4H), 7.30 (t, *J* = 7.2 Hz, 1H), 7.21 (td, *J* = 7.8, 1.2 Hz, 1H), 7.16 (d, *J* = 6.6 Hz, 1H), 7.09 (s, 1H), 7.04 – 7.01 (m, 2H), 6.86 –6.83 (m, 2H), 6.79 (d, *J* = 7.8 Hz, 1H), 6.72 (d, *J* = 7.2 Hz, 1H), 5.13 (d, *J* = 15.6 Hz, 1H), 4.91 (d, *J* = 15.6 Hz, 1H), 3.72 (dd, *J* = 15.6, 1.8 Hz, 1H), 3.36 (dd, *J* = 15.6, 2.4 Hz, 1H). **^{13}C NMR** (151 MHz, $CDCl_3$) δ 179.1, 148.1, 142.5, 141.7, 140.1, 136.1, 135.2, 134.4, 133.8, 131.7, 129.0, 128.9, 128.4, 128.3, 127.7, 127.7, 127.4, 126.4, 126.1, 126.1, 125.7, 125.4, 125.0, 123.6, 123.5, 123.1, 120.9, 109.2, 57.7, 45.9, 44.1. HRMS (ESI) m/z: $[M + H]^+$ calcd for $C_{34}H_{26}NO$ 464.2009; found 464.2010.

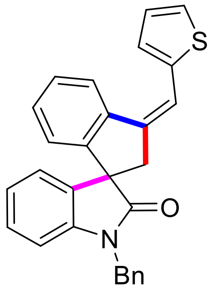

3ae-l

(Z)-1'-benzyl-3-(thiophen-2-ylmethylene)-2,3-dihydrospiro[indene-1,3'-indolin]-2'-one Colorless viscous liquid, isolated yield 46% (38.6 mg) (eluent: petroleum ether/EtOAc = 25:1); **^{1}H NMR** (600 MHz, Chloroform-*d*) δ 7.68 – 7.65 (m, 1H), 7.38 – 7.29 (m, 6H), 7.22 – 7.17 (m, 2H), 7.15 – 7.13 (m, 2H), 7.09 – 7.07 (m, 2H), 6.99 (t, *J* = 7.5 Hz, 1H), 6.82 (d, *J* = 7.8 Hz, 1H), 6.77 – 6.74 (m, 1H), 6.69 (s, 1H), 5.11 (d, *J* = 15.0 Hz, 1H), 4.89 (d, *J* = 15.6 Hz, 1H), 3.58 (d, *J* = 15.6 Hz, 1H), 3.18 (d, *J* = 15.6 Hz, 1H). **^{13}C NMR** (151 MHz, $CDCl_3$) δ 178.9, 148.5, 142.3, 142.1, 139.6, 139.6, 136.0, 134.2, 129.5, 128.9, 128.3, 127.8, 127.7, 127.4, 127.3, 126.4, 125.1, 124.8, 123.7, 123.4, 123.1, 114.9, 109.2, 57.4, 46.4, 44.1 HRMS (ESI) m/z: $[M + H]^+$ calcd for $C_{28}H_{22}NOS$ 420.1417; found 420.1424.

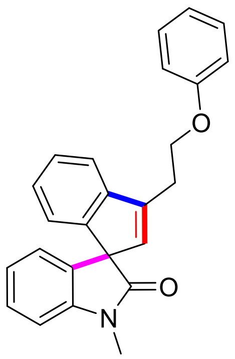

3af

1'-methyl-3-(2-phenoxyethyl)spiro[indene-1,3'-indolin]-2'-one White solid, isolated yield 33% (24.3 mg) (eluent: petroleum ether/EtOAc = 25:1); **^{1}H NMR** (600 MHz, Chloroform-*d*) δ 7.43 (d, *J* = 7.8 Hz, 1H), 7.35 – 7.27 (m, 4H), 7.14 (t, *J* = 7.2 Hz, 1H), 6.97– 6.91 (m, 6H), 6.74 (d, *J* = 7.8 Hz, 1H), 6.07 (s, 1H), 4.34 (t, *J* = 6.9 Hz, 2H), 3.33 (s, 3H), 3.16 (t, *J* = 6.9 Hz, 2H). **^{13}C NMR** (151 MHz, $CDCl_3$) δ 174.2, 157.7, 144.9, 144.2, 143.8, 143.7, 130.1, 128.5, 127.6, 127.3, 126.9, 125.5, 122.5, 121.9, 121.3, 119.8, 118.8, 113.5, 107.3, 64.9, 63.5, 26.8, 25.9. HRMS (ESI) m/z: $[M + H]^+$ calcd for $C_{25}H_{22}NO_2$ 368.1645; found 368.1646.

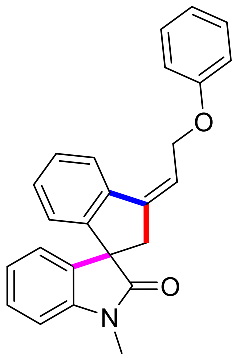

3af-I

(Z)-1'-methyl-3-(2-phenoxyethylidene)-2,3-dihydrospiro[indene-1,3'-indolin]-2'-one Colorless viscous liquid, isolated yield 40% (29.4 mg) (eluent: petroleum ether/EtOAc = 25:1); **^{1}H NMR** (400 MHz, Chloroform-*d*) δ 7.59 (d, *J* = 7.6 Hz, 1H), 7.35 – 7.28 (m, 4H), 7.19 (t, *J* = 7.4 Hz, 1H), 7.02 – 6.95 (m, 5H), 6.92 (d, *J* = 8.0 Hz, 1H), 6.78 (d, *J* = 7.6 Hz, 1H), 6.00 (t, *J* = 6.4 Hz, 1H), 5.10 (dd, *J* = 12.4, 6.8 Hz, 1H), 4.97 (dd, *J* = 12.4, 6.0 Hz, 1H), 3.43 (dd, *J* = 15.6, 1.6 Hz, 1H), 3.30(s, 3H), 3.07 (dd, *J* = 15.2, 1.4 Hz, 1H). **^{13}C NMR** (101 MHz, $CDCl_3$) δ 178.7, 158.5, 148.1, 143.2, 142.5, 139.8, 134.2, 129.6, 129.3, 128.4, 128.3, 125.3, 124.0, 123.3, 123.1, 120.9, 118.9, 114.9, 108.1, 64.7, 57.7, 45.5, 26.6. HRMS (ESI) m/z: $[M + H]^+$ calcd for $C_{25}H_{22}NO_2$ 368.1645; found 368.1637.

4. References

1. A. Lu, X. Ji , B. Zhou, Z. Wu and Y. Zhang, Palladium-Catalyzed C–H Silylation through Palladacycles Generated from Aryl Halides, Angew. Chem. Int. Ed., 2018, 57, 3233.

2. Y. Gao, D. E. Hill, W. Hao, B. J. McNicholas, J. C. Vantourout, R. G. Hadt, S. E. Reisman, D. G. Blackmond and P. S. Baran, Electrochemical Nozaki−Hiyama−Kishi Coupling: Scope, Applications, and Mechanism, J. Am. Chem. Soc., 2021, 143, 9478.

5. Scanned ^{1}H NMR and ^{13}C NMR Spectra of the Products

^{1}H NMR of **3aa** (400 MHz, Chloroform-*d*) and ^{13}C NMR of **3aa** (151 MHz, Chloroform-*d*)

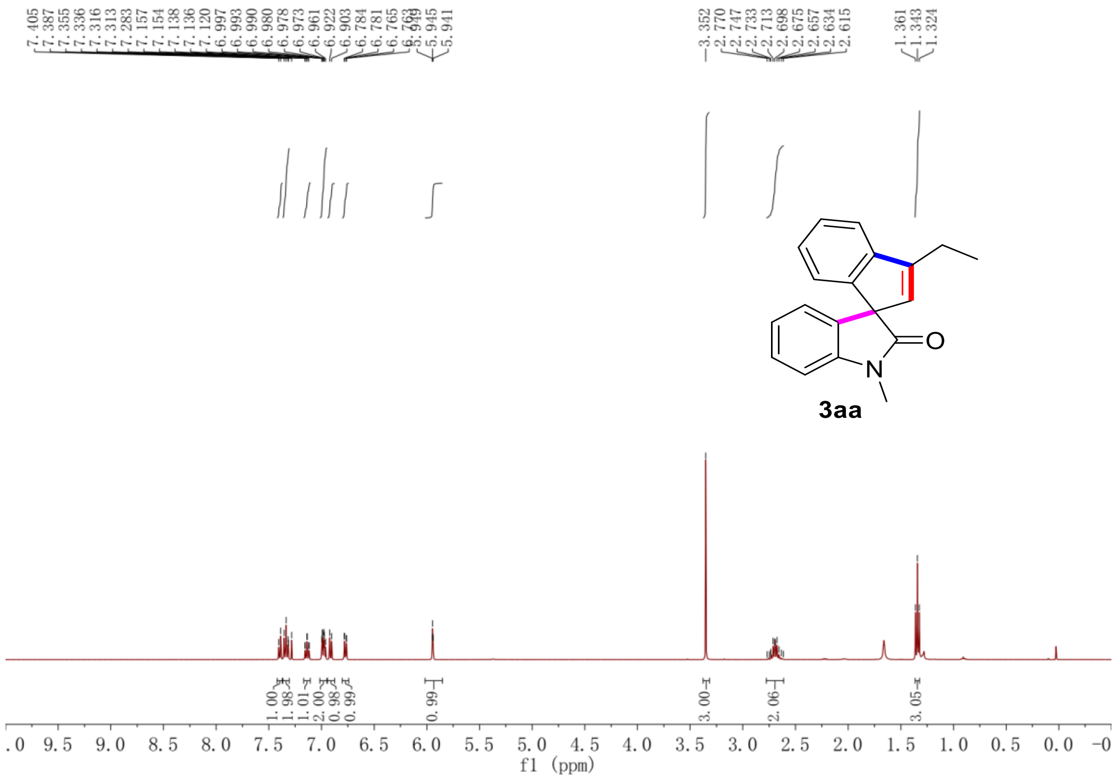

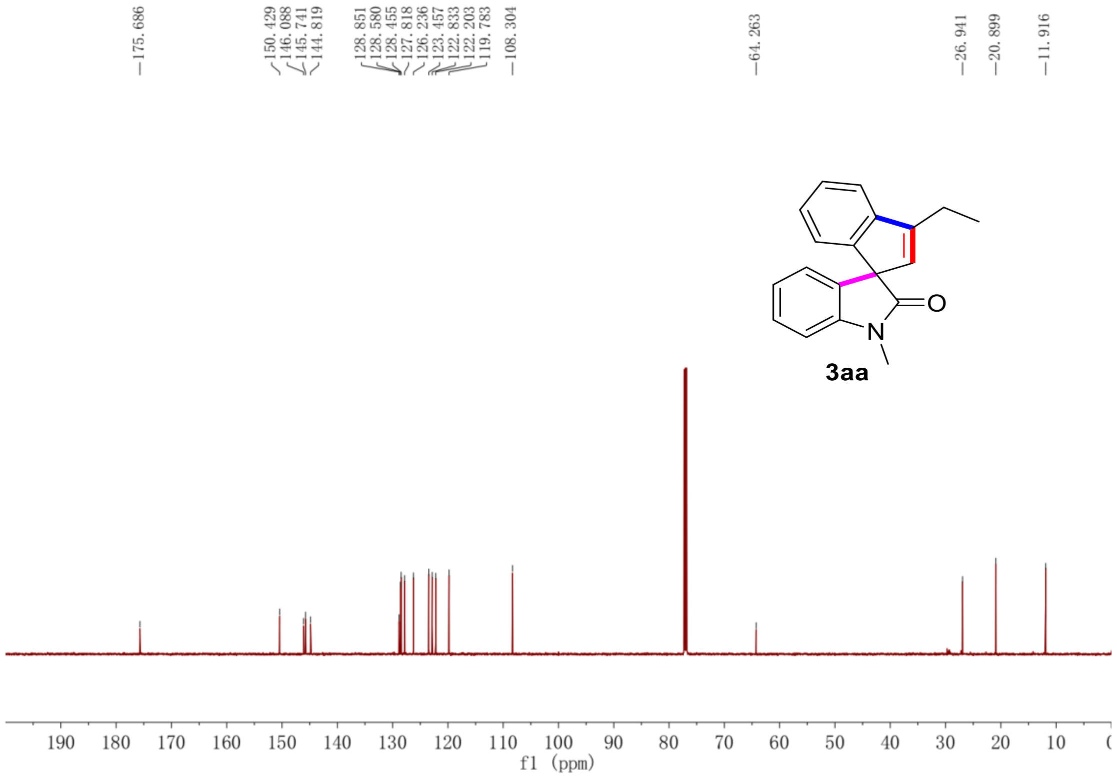

^{1}H NMR of **3ba** (600 MHz, Chloroform-*d*) and ^{13}C NMR of **3ba** (151 MHz, Chloroform-*d*)

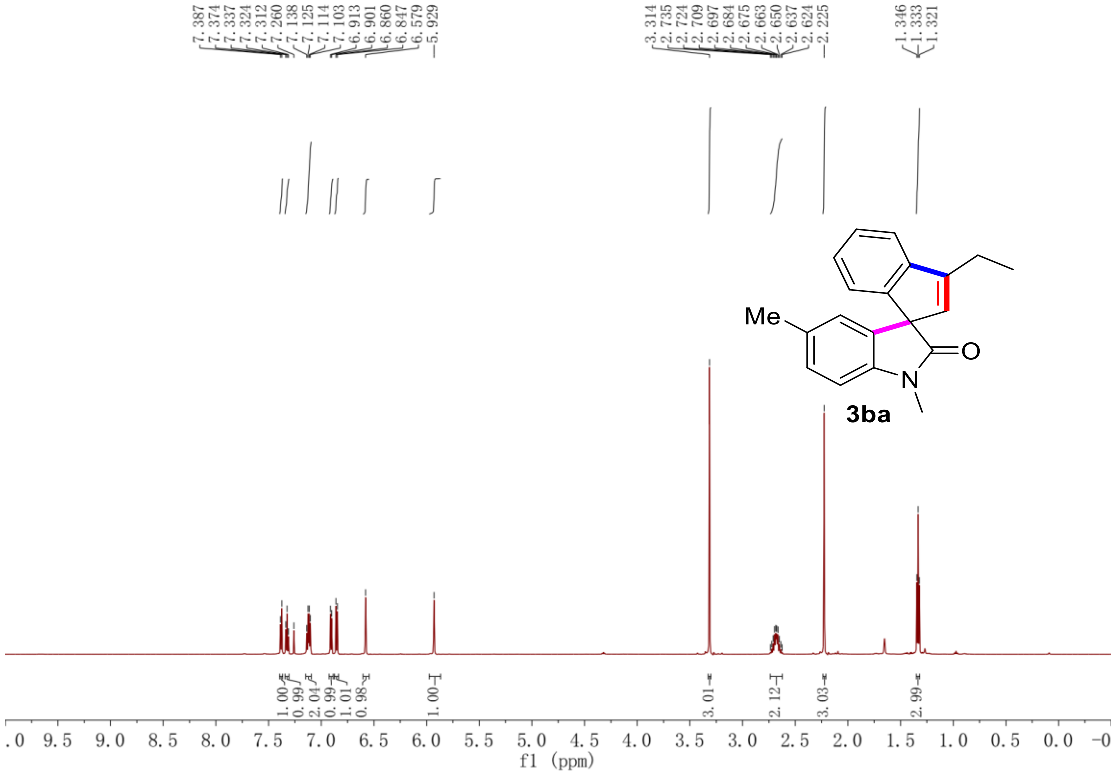

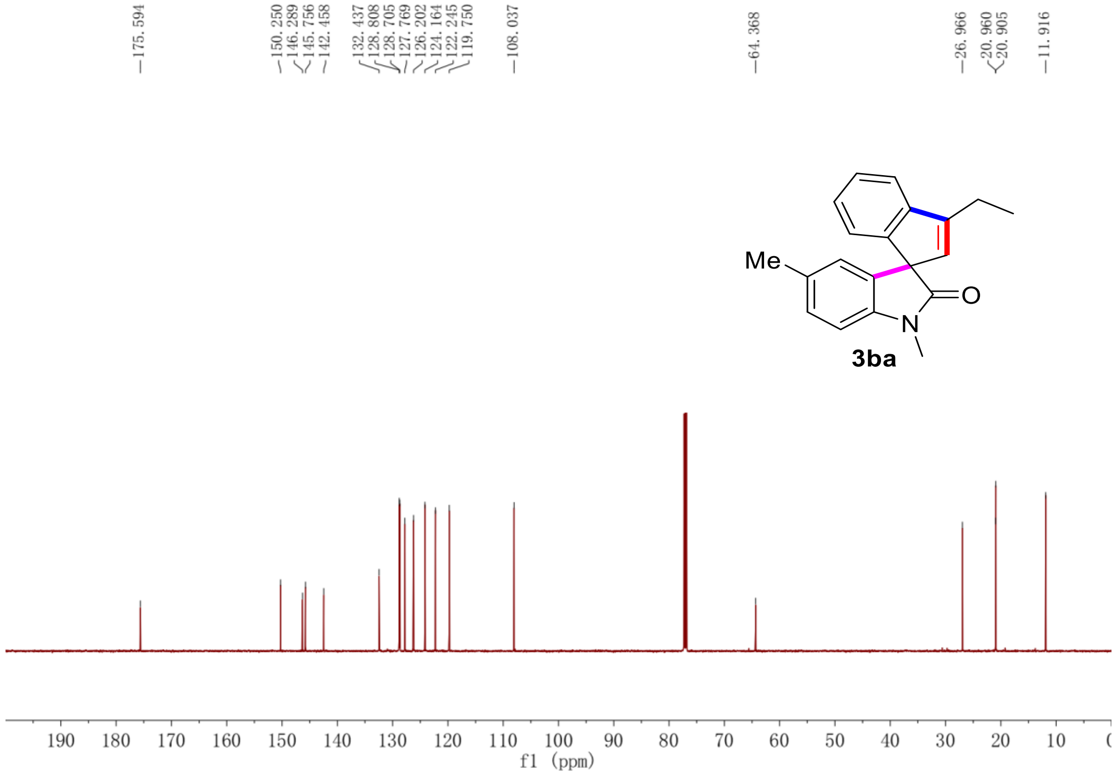

^{1}H NMR of **3ca** (600 MHz, Chloroform-*d*) and ^{13}C NMR of **3ca** (151 MHz, Chloroform-*d*)

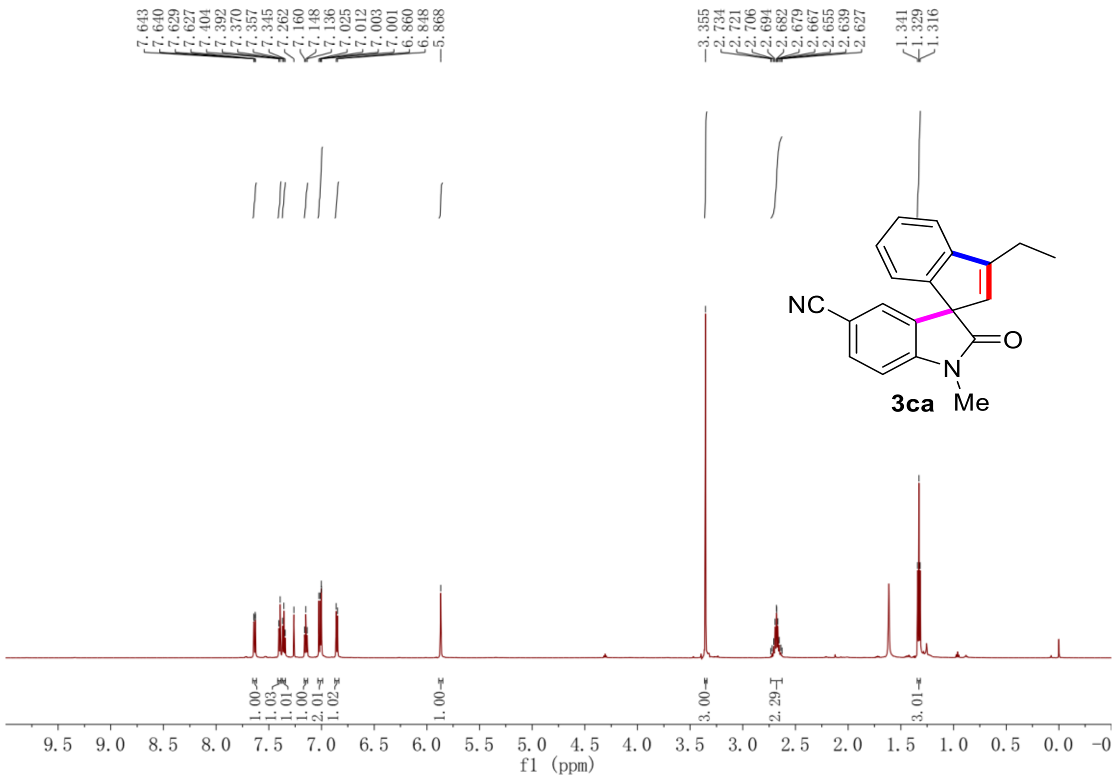

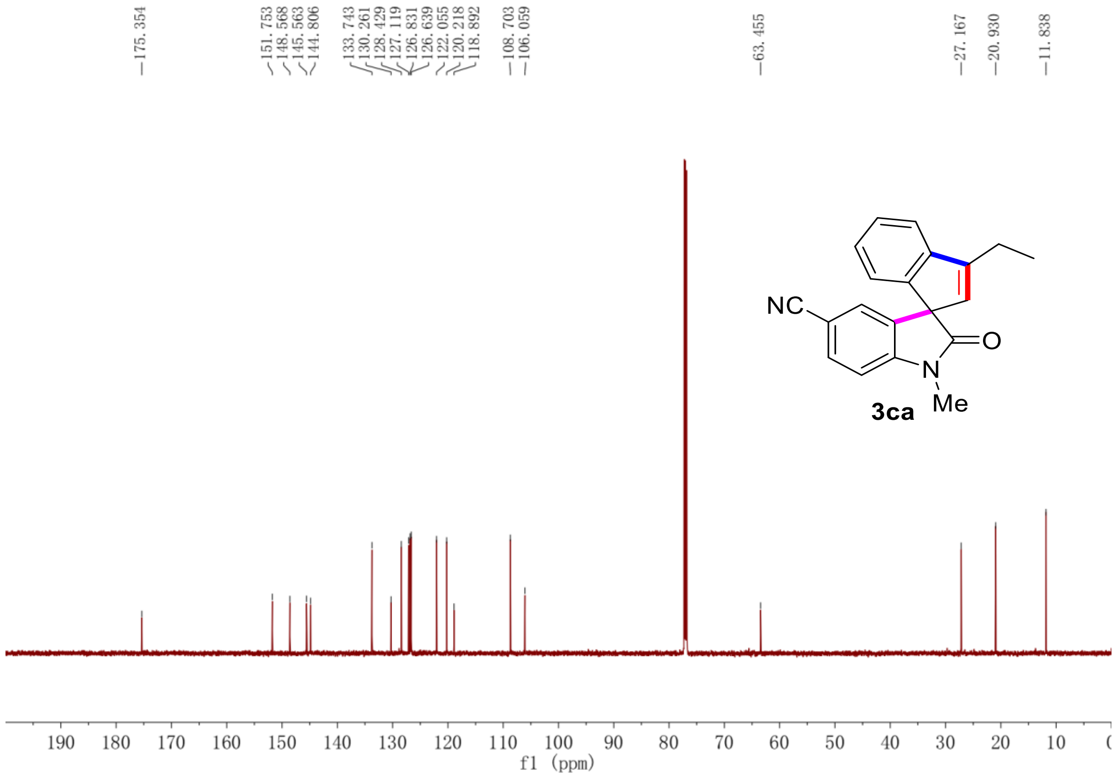

^{1}H NMR of **3da** (600 MHz, Chloroform-*d*) and ^{13}C NMR of **3da** (151 MHz, Chloroform-*d*)

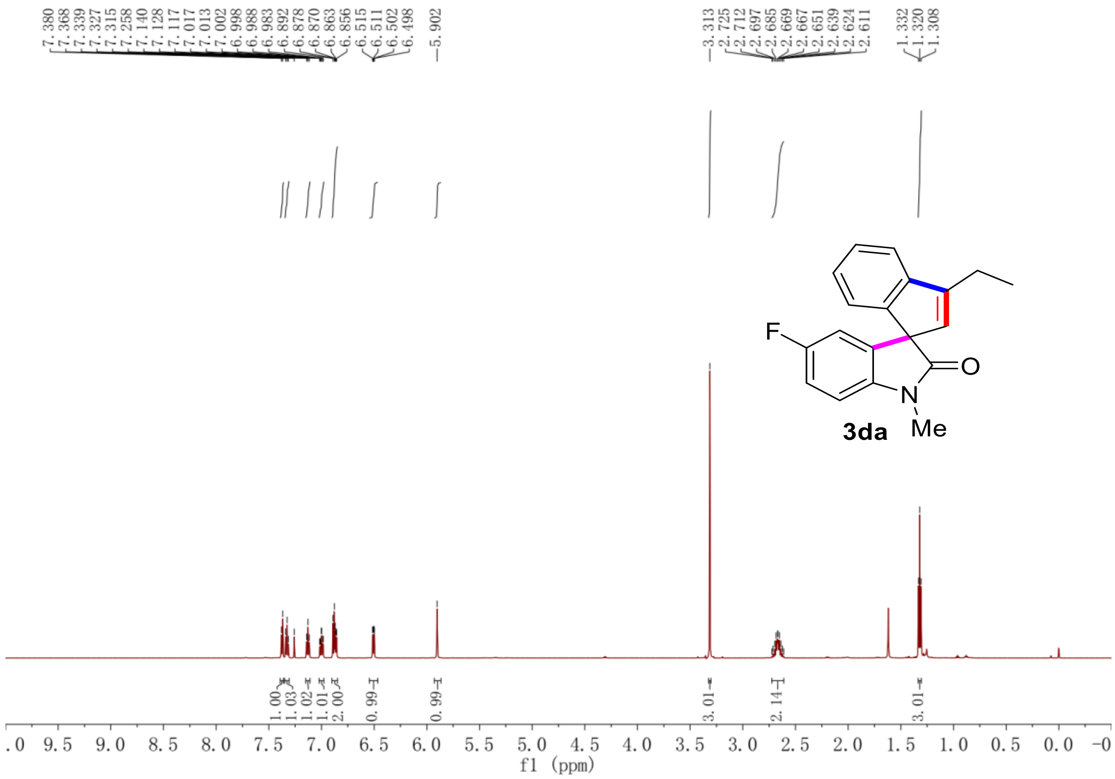

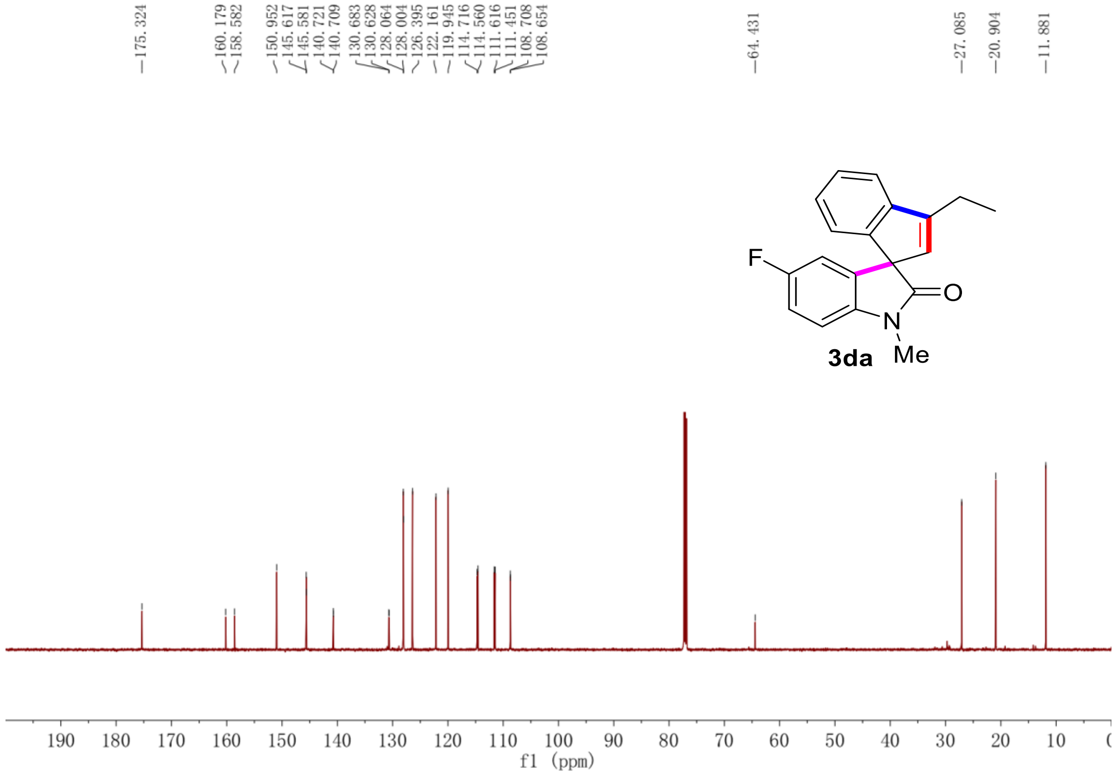

^{1}H NMR of **3ea** (600 MHz, Chloroform-*d*) and ^{13}C NMR of **3ea** (151 MHz, Chloroform-*d*)

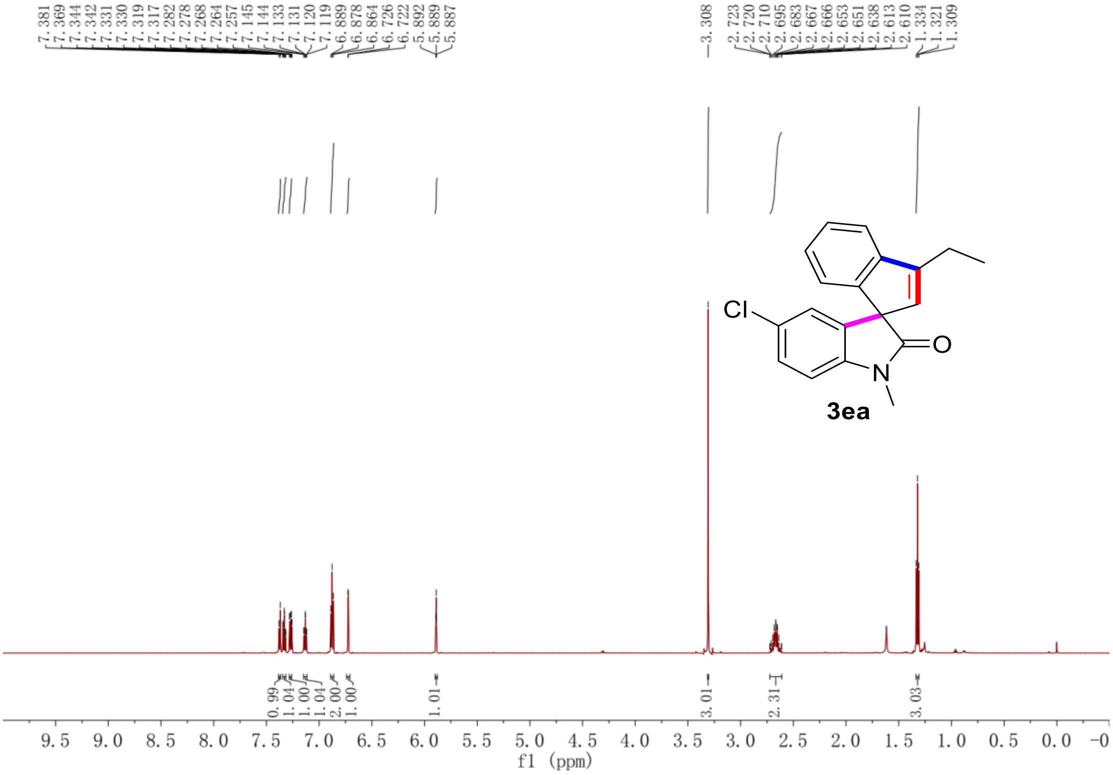

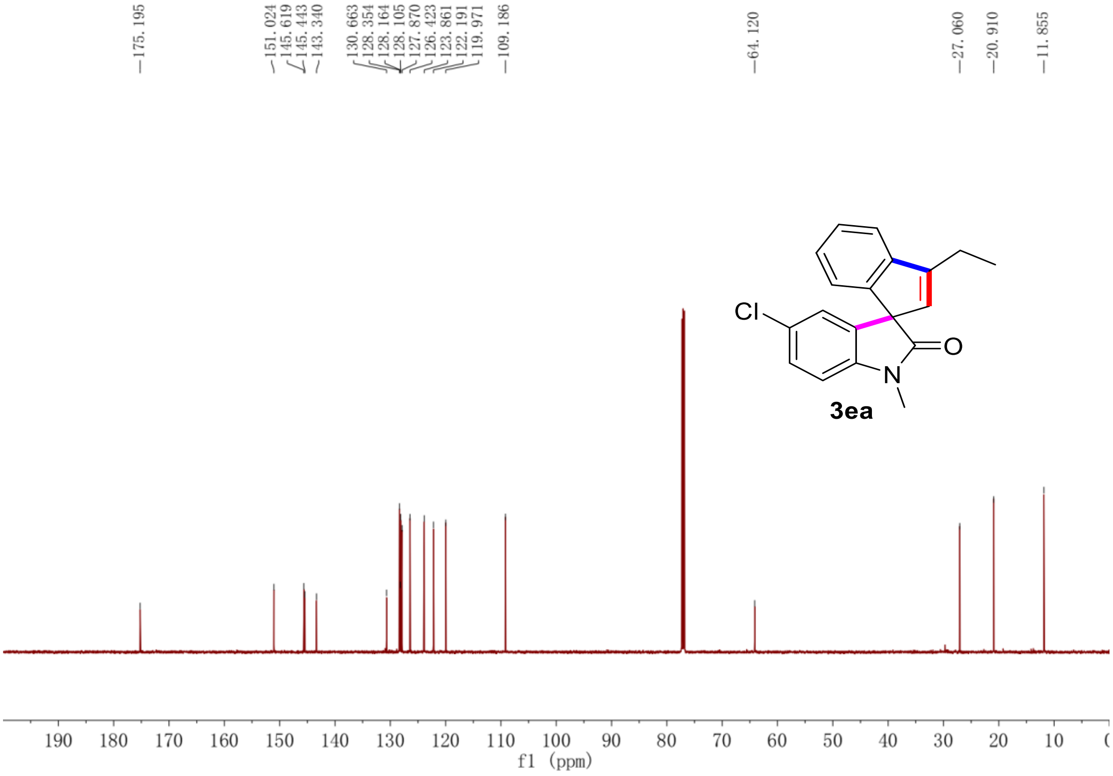

^{1}H NMR of **3fa** (600 MHz, Chloroform-*d*) and ^{13}C NMR of **3fa** (151 MHz, Chloroform-*d*)

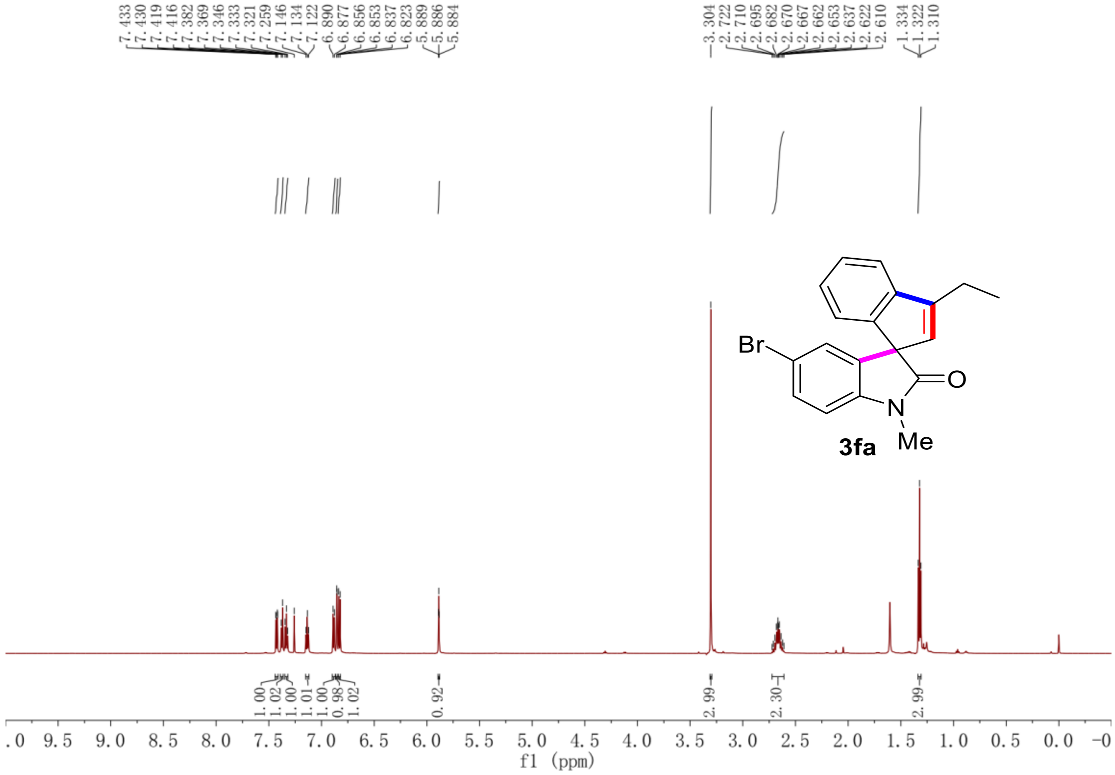

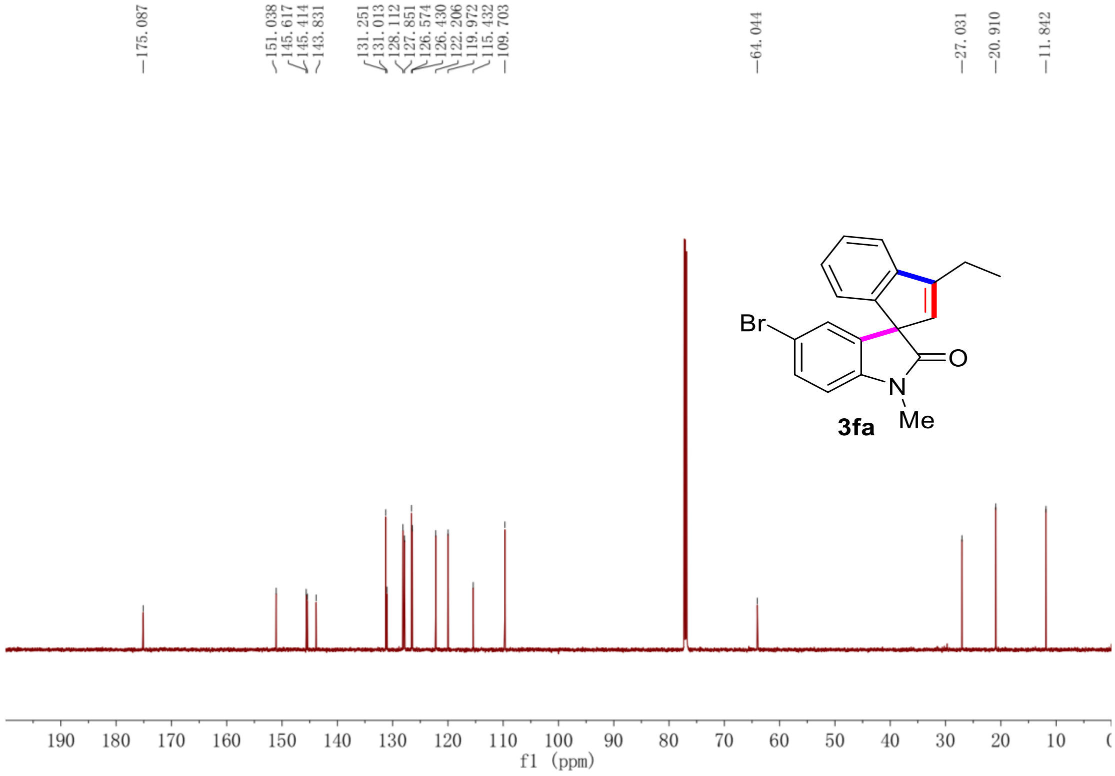

^{1}H NMR of **3ga** (600 MHz, Chloroform-*d*) and ^{13}C NMR of **3ga** (151 MHz, Chloroform-*d*)

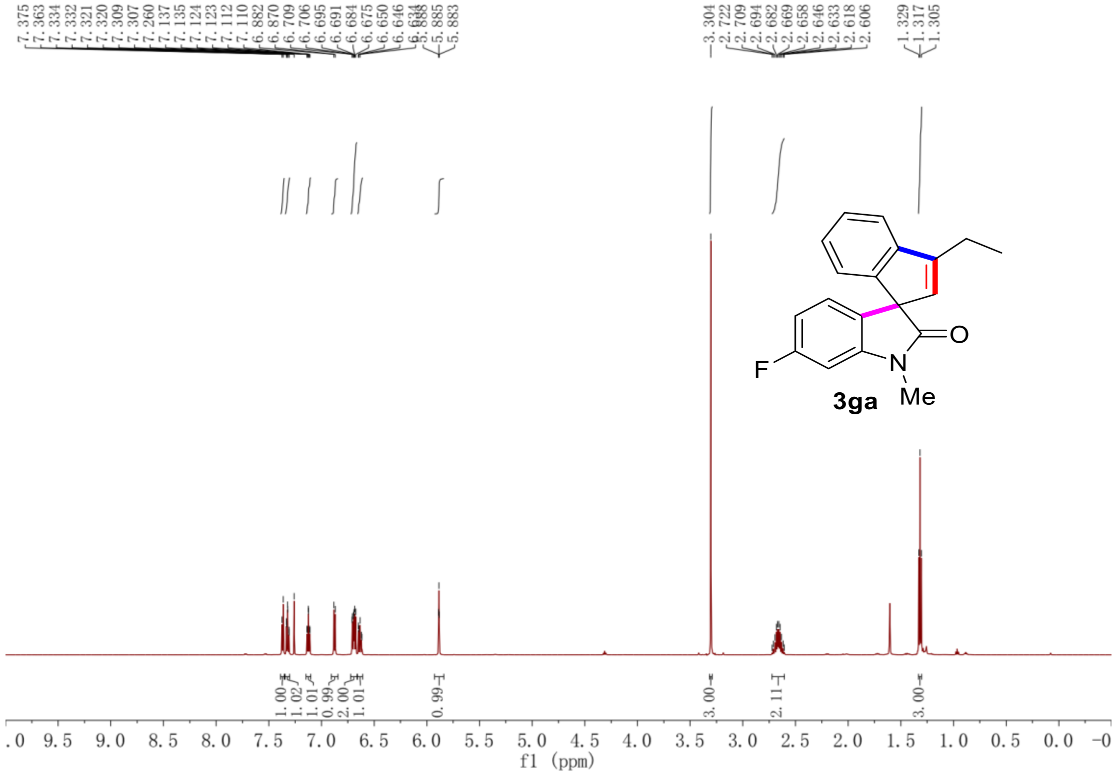

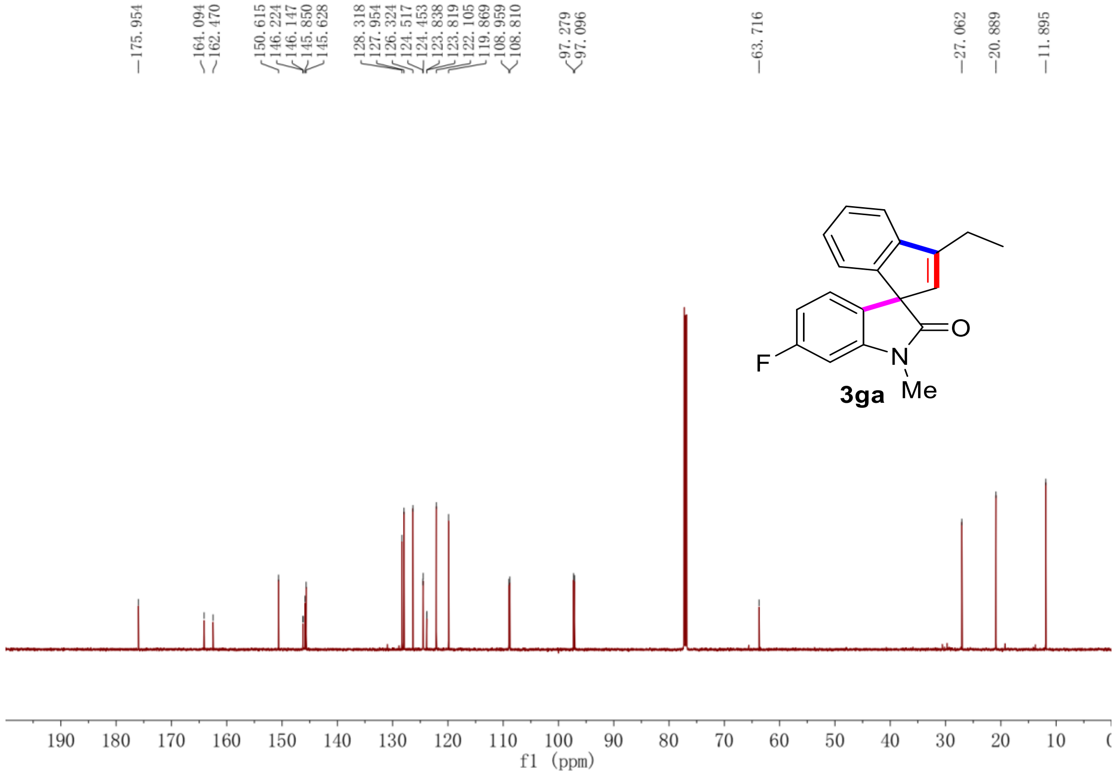

^{1}H NMR of **3ha** (600 MHz, Chloroform-*d*) and ^{13}C NMR of **3ha** (151 MHz, Chloroform-*d*)

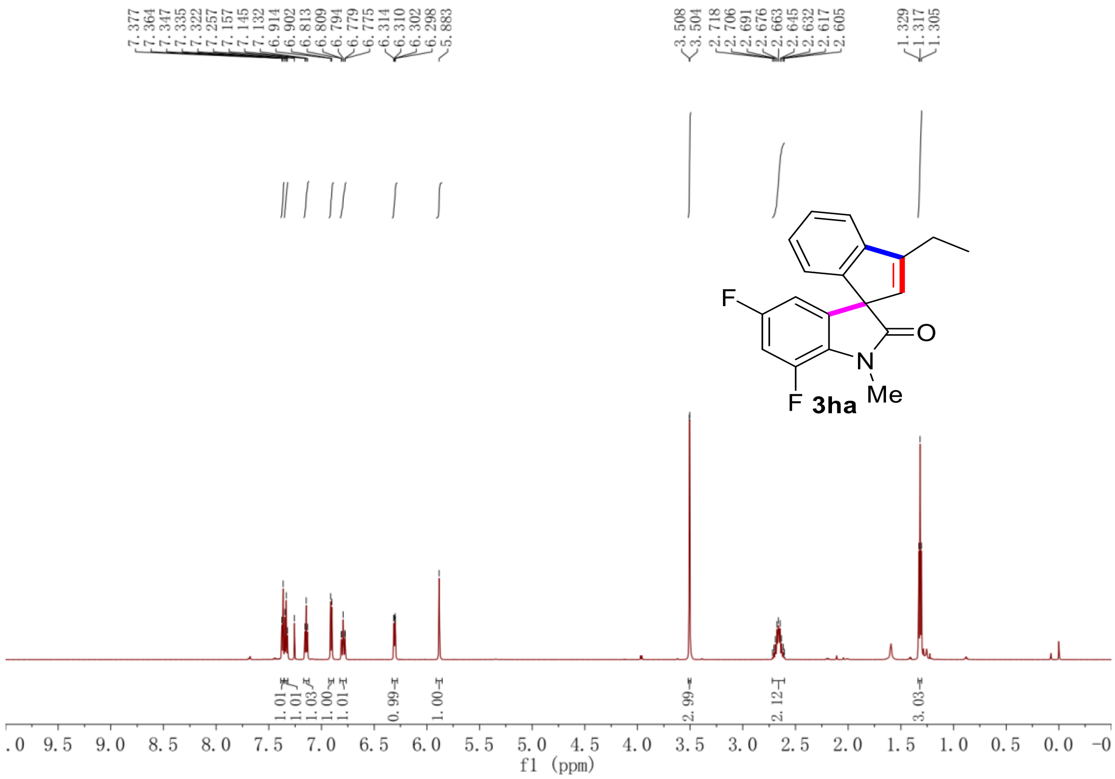

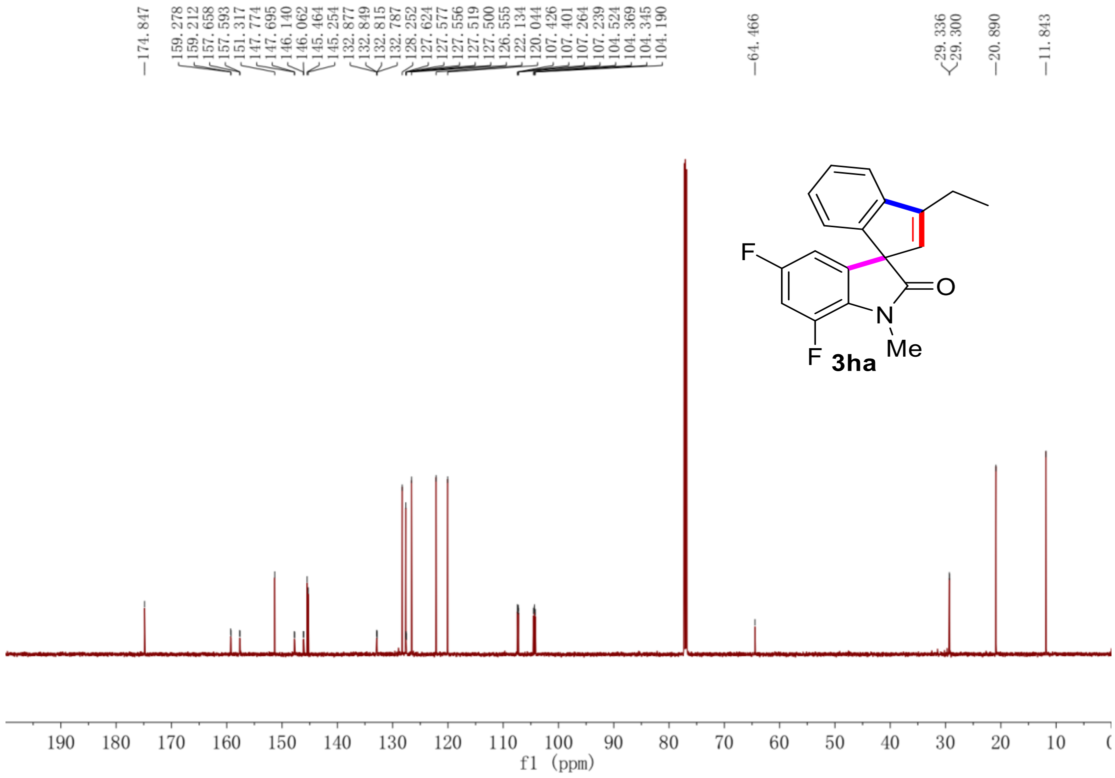

^{1}H NMR of **3ia** (600 MHz, Chloroform-*d*) and ^{13}C NMR of **3ia** (151 MHz, Chloroform-*d*)

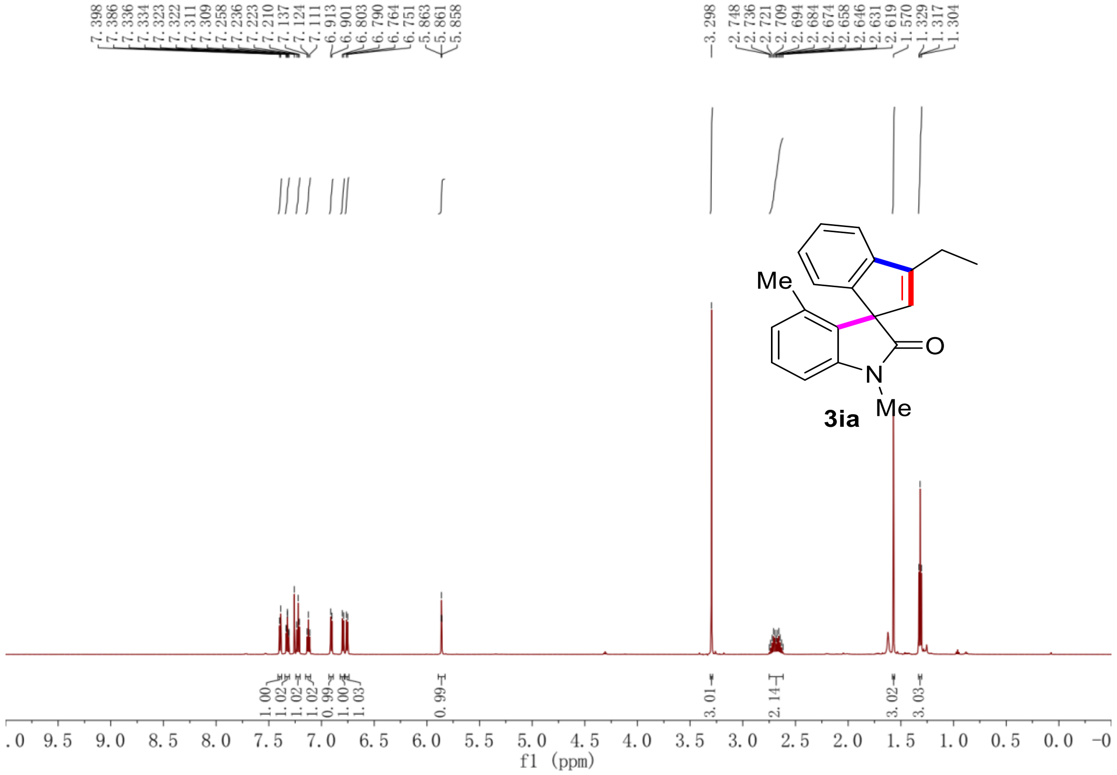

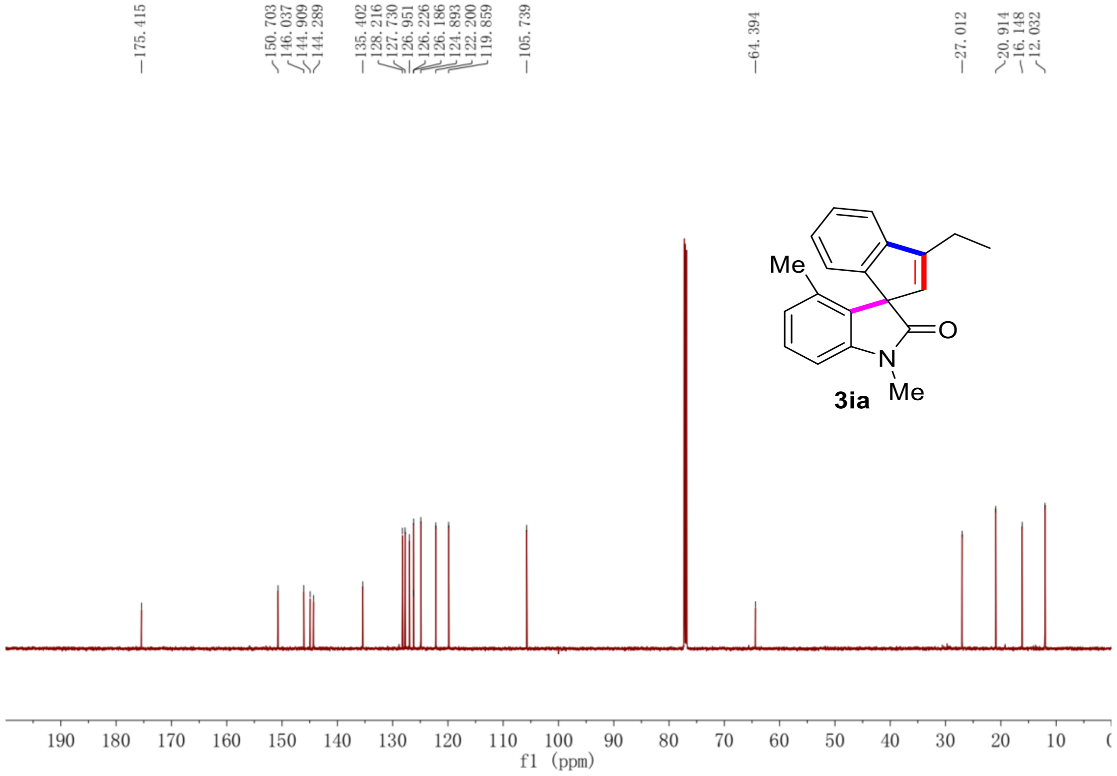

^{1}H NMR of **3ja** (600 MHz, Chloroform-*d*) and ^{13}C NMR of **3ja** (151 MHz, Chloroform-*d*)

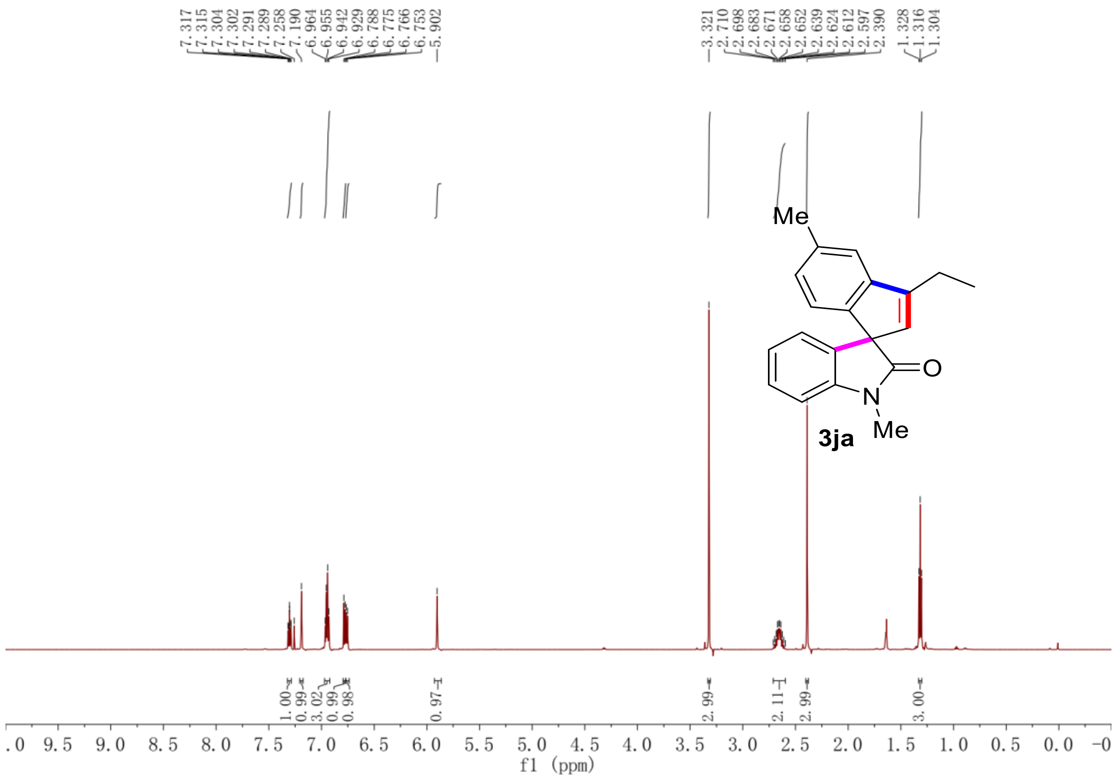

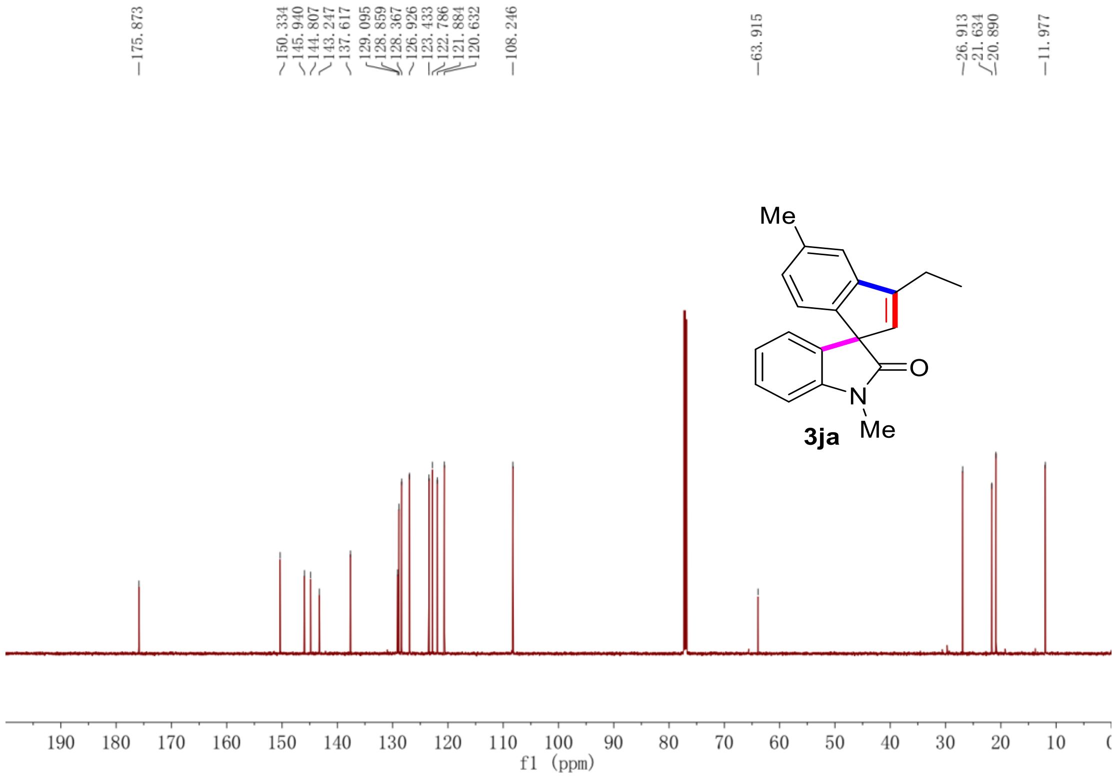

^{1}H NMR of **3ka** (600 MHz, Chloroform-*d*) and ^{13}C NMR of **3ka** (151 MHz, Chloroform-*d*)

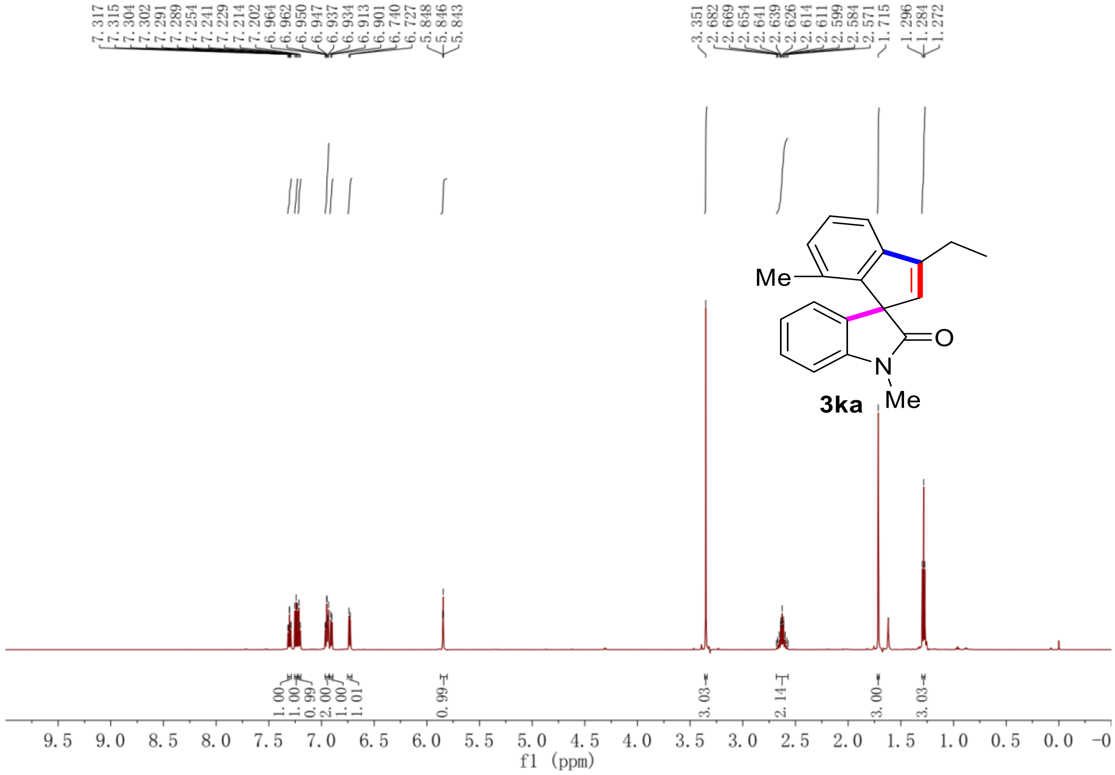

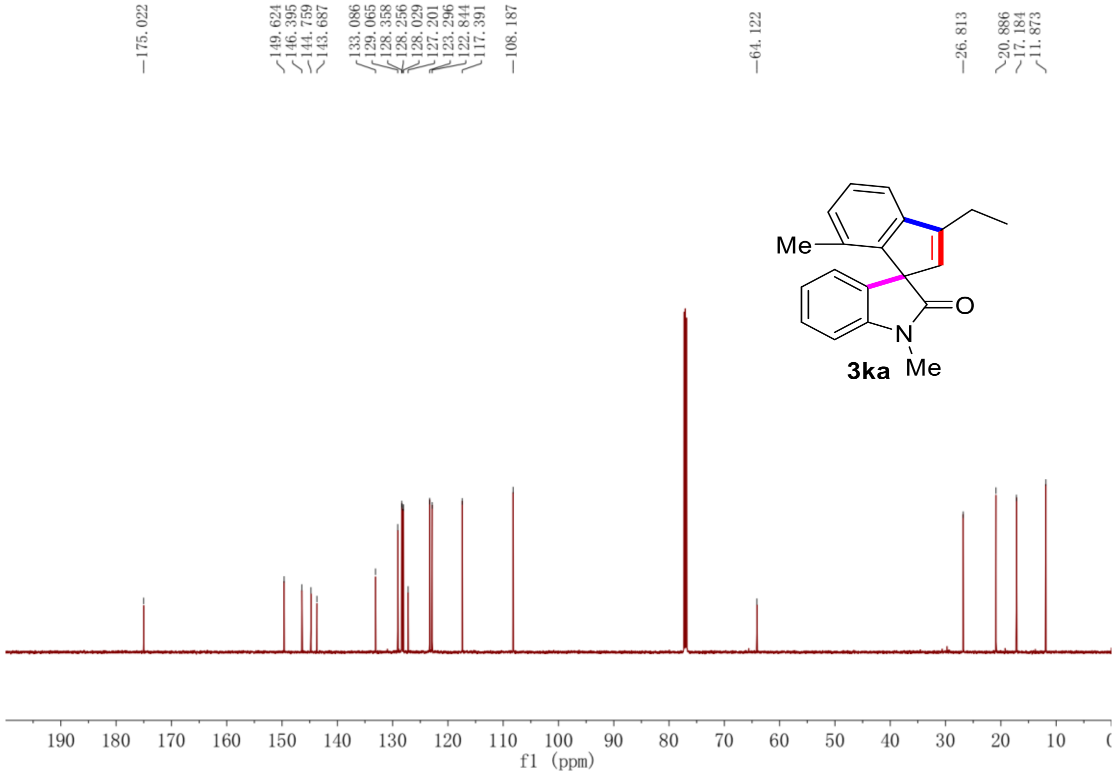

^{1}H NMR of **3la** (600 MHz, Chloroform-*d*) and ^{13}C NMR of **3la** (151 MHz, Chloroform-*d*)

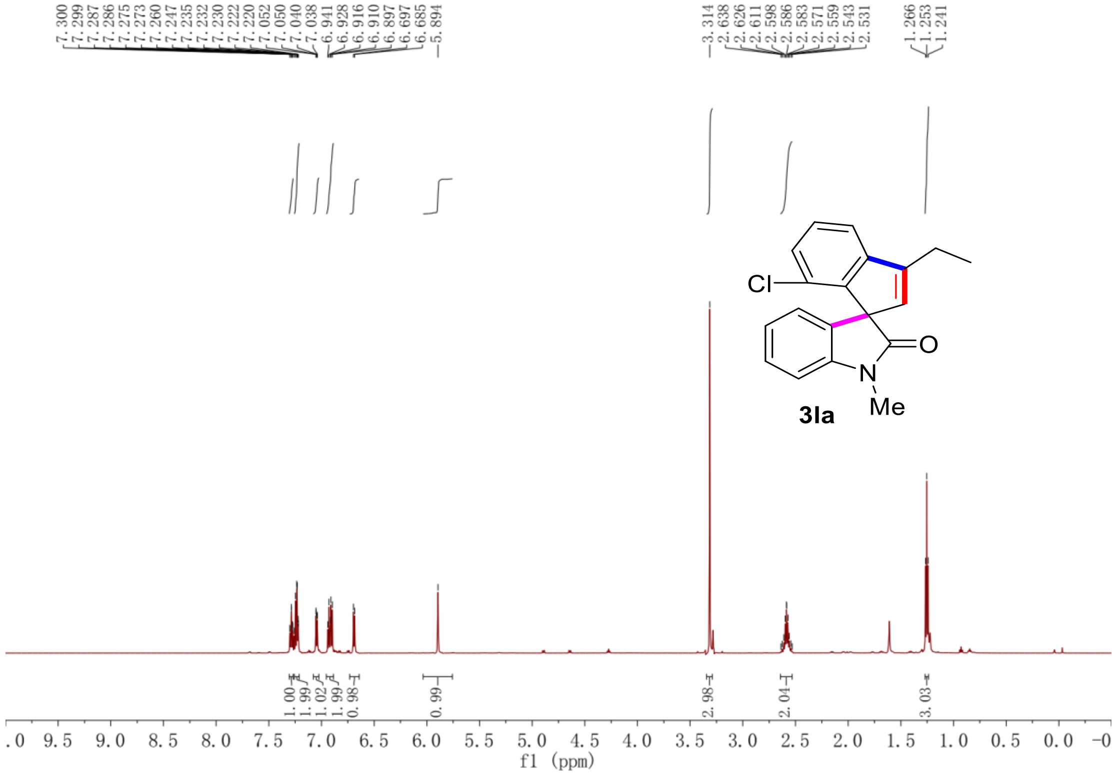

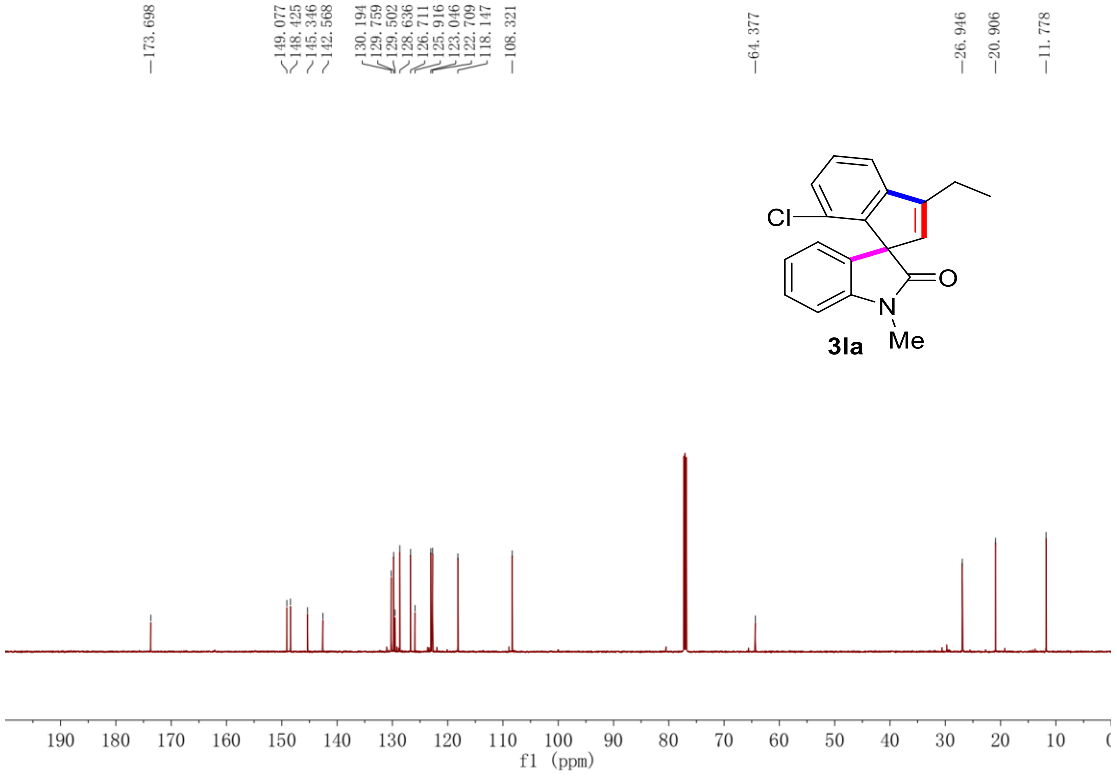

^{1}H NMR of **3ma** (600 MHz, Chloroform-*d*) and ^{13}C NMR of **3ma** (151 MHz, Chloroform-*d*)

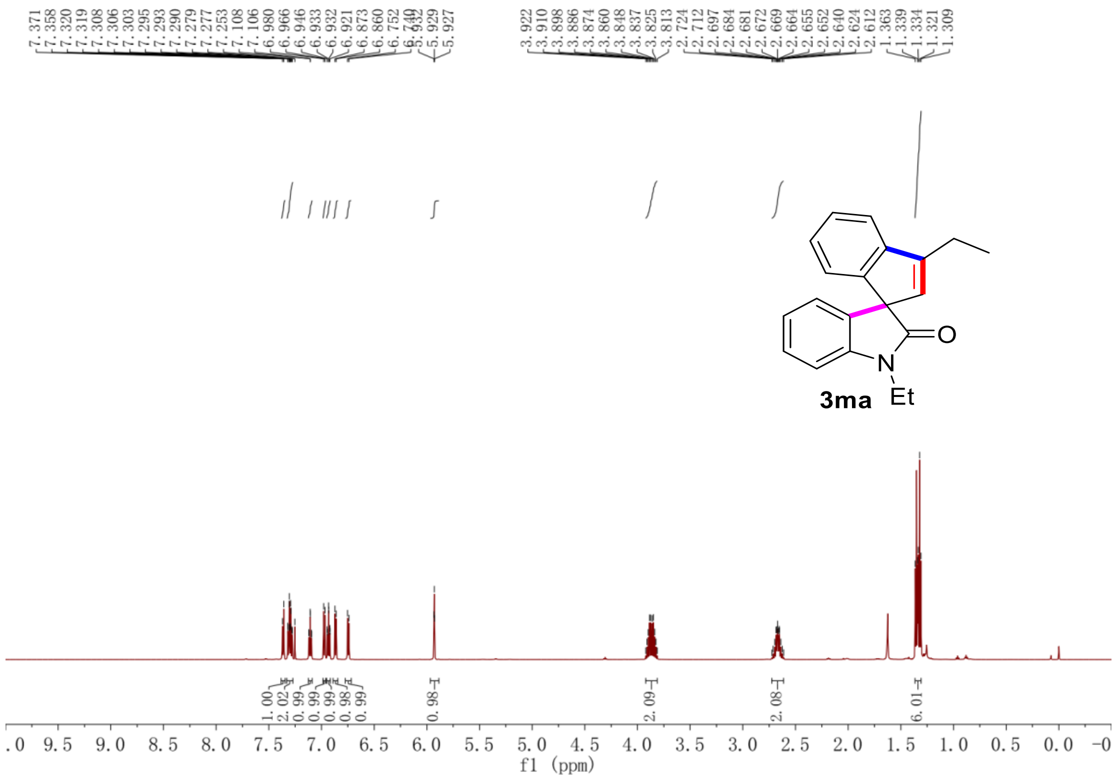

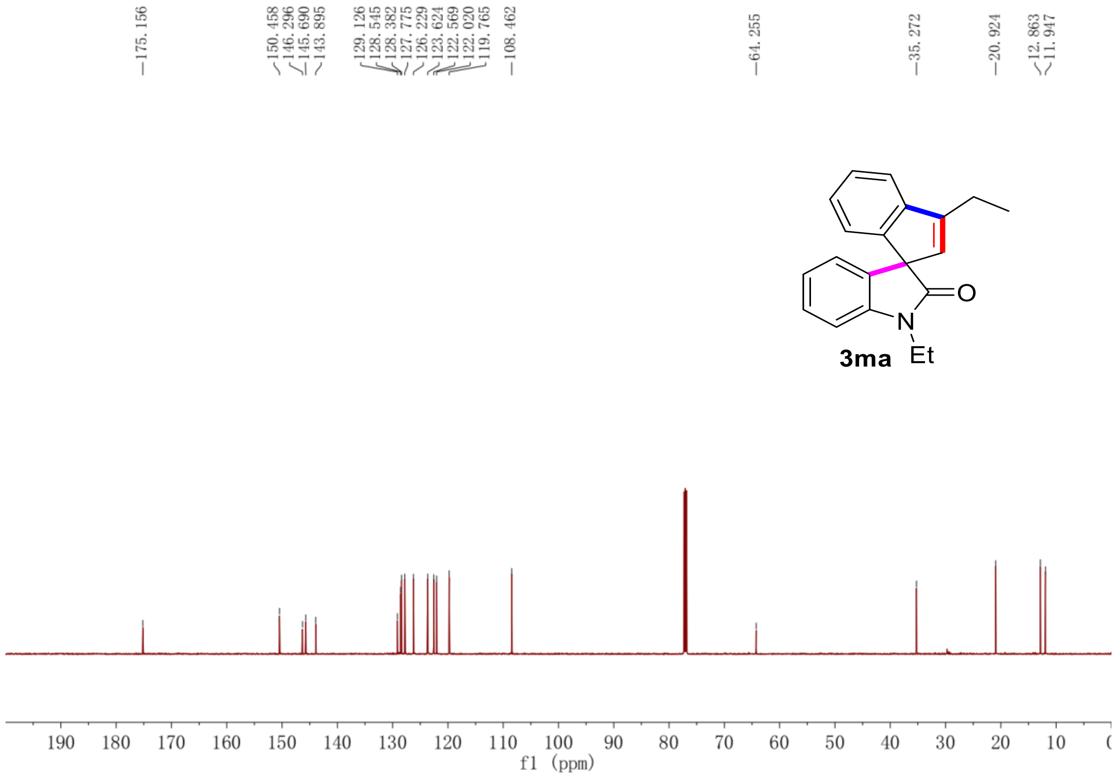

^{1}H NMR of **3na** (600 MHz, Chloroform-*d*) and ^{13}C NMR of **3na** (151 MHz, Chloroform-*d*)

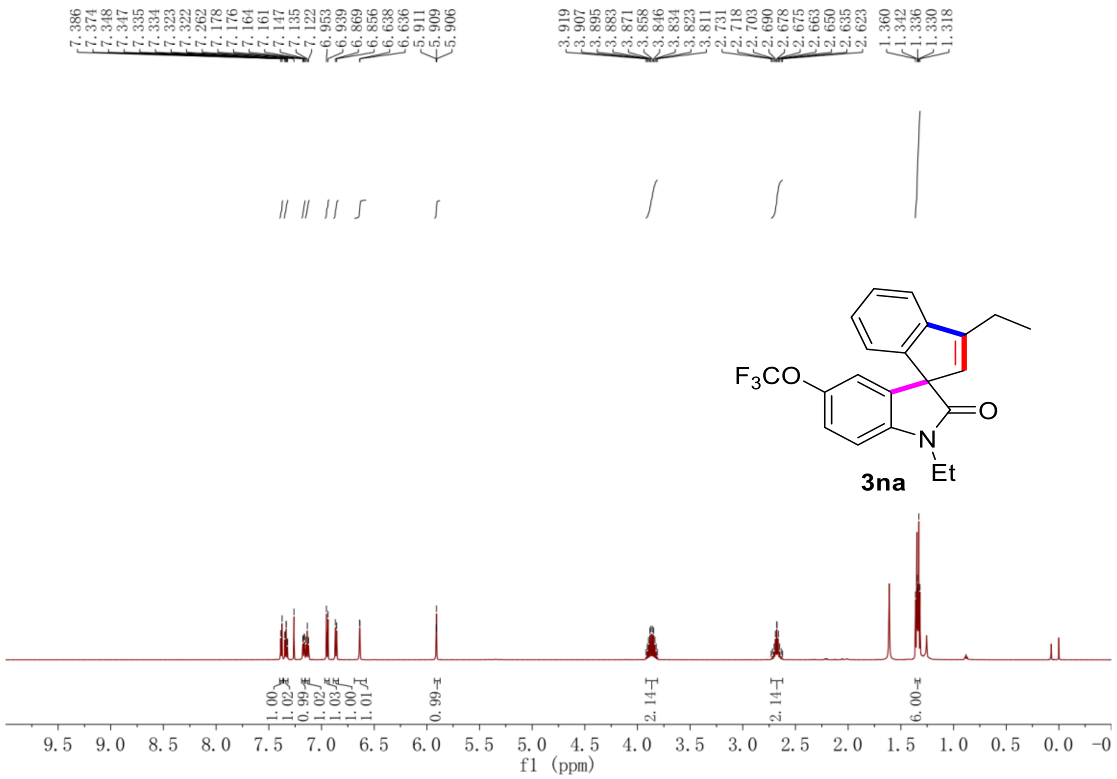

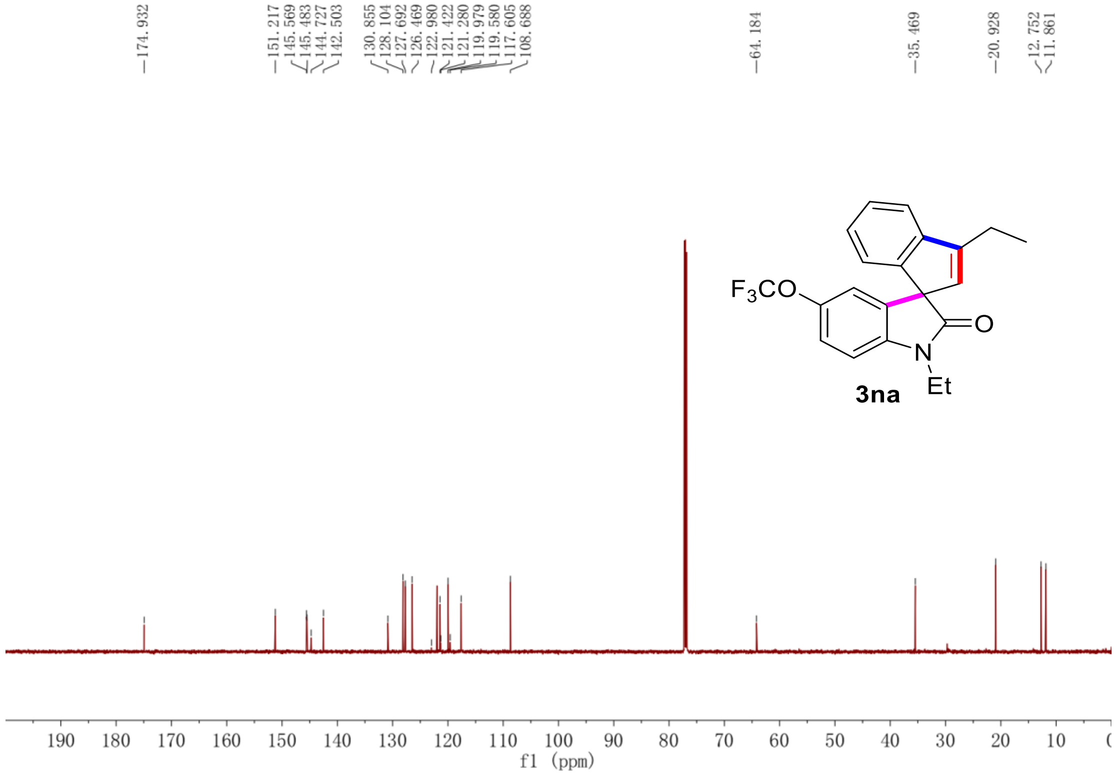

^{1}H NMR of **3oa** (600 MHz, Chloroform-*d*) and ^{13}C NMR of **3oa** (151 MHz, Chloroform-*d*)

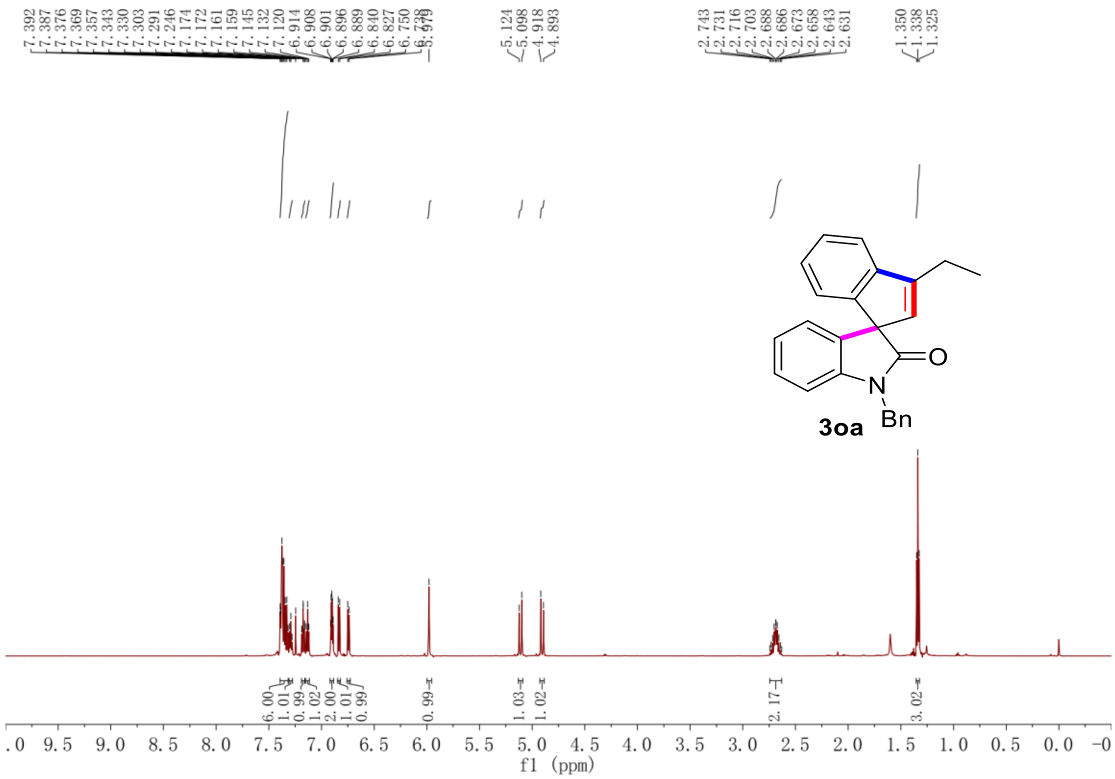

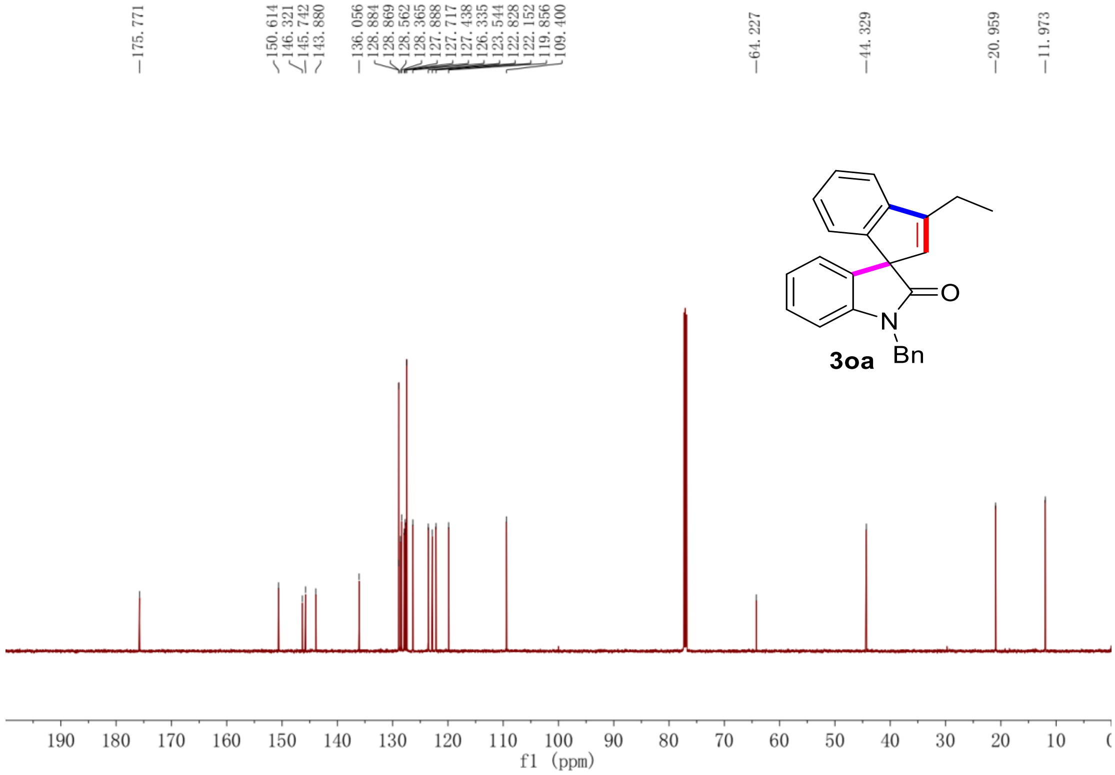

^{1}H NMR of **3pa** (600 MHz, Chloroform-*d*) and ^{13}C NMR of **3pa** (151 MHz, Chloroform-*d*)

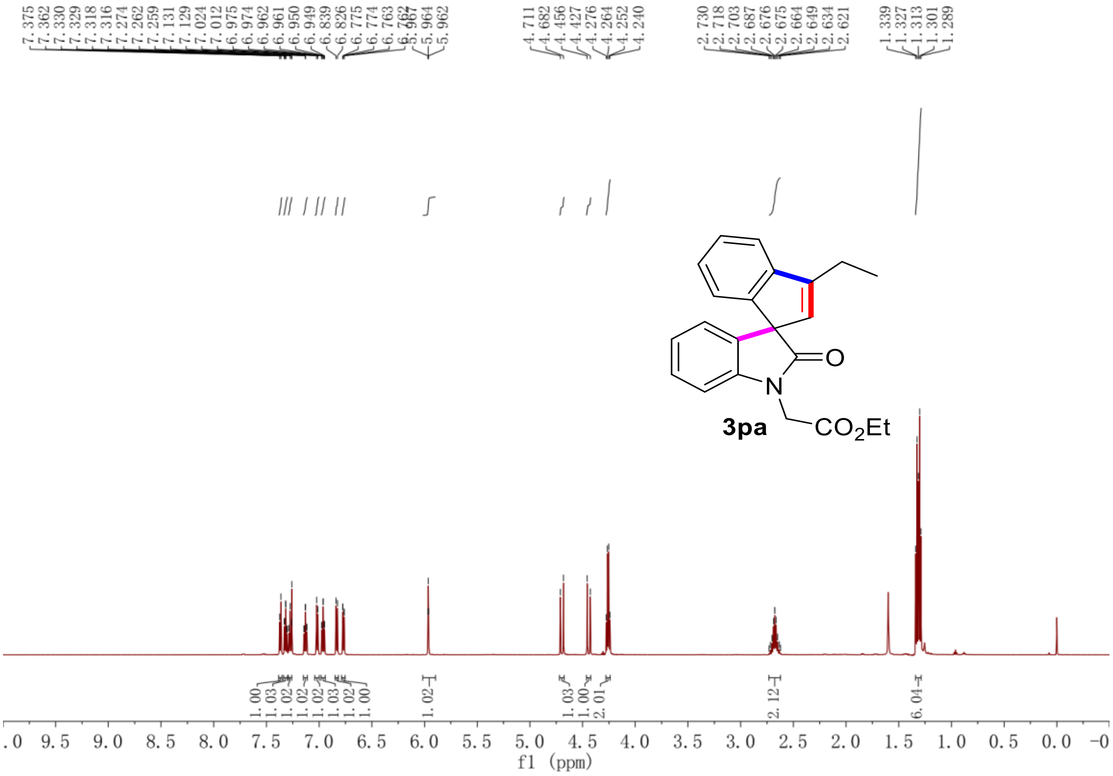

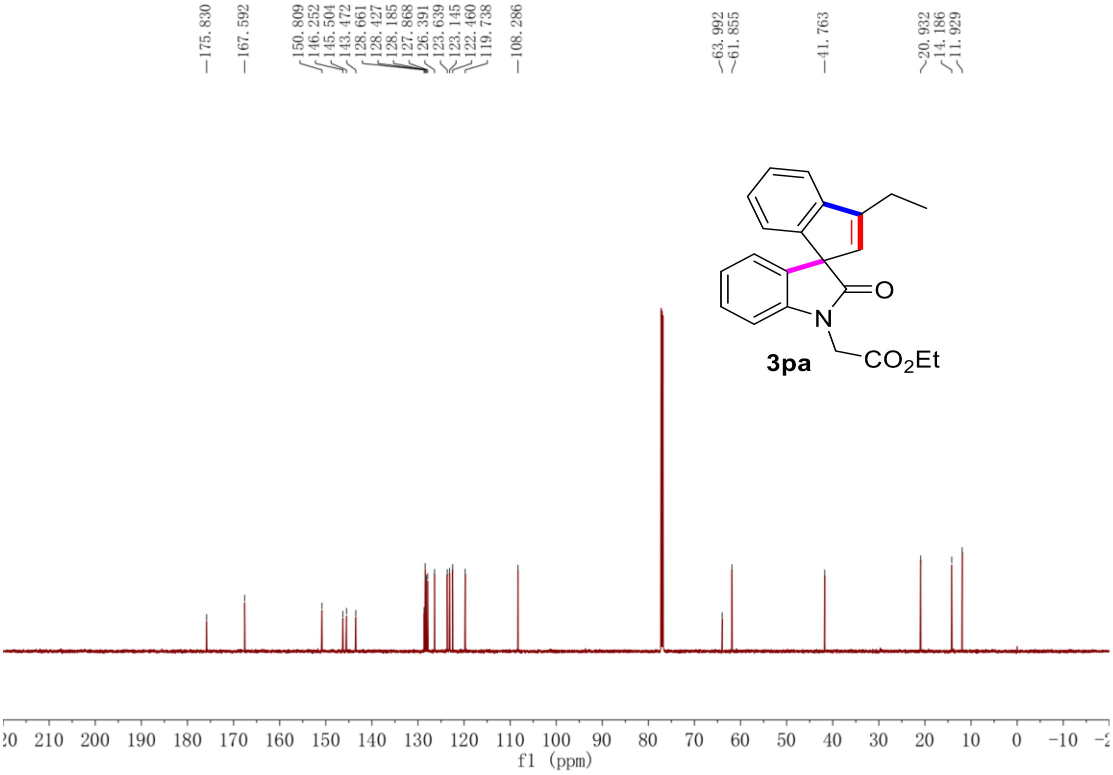

^{1}H NMR of **3qa** (600 MHz, Chloroform-*d*) and ^{13}C NMR of **3qa** (151 MHz, Chloroform-*d*)

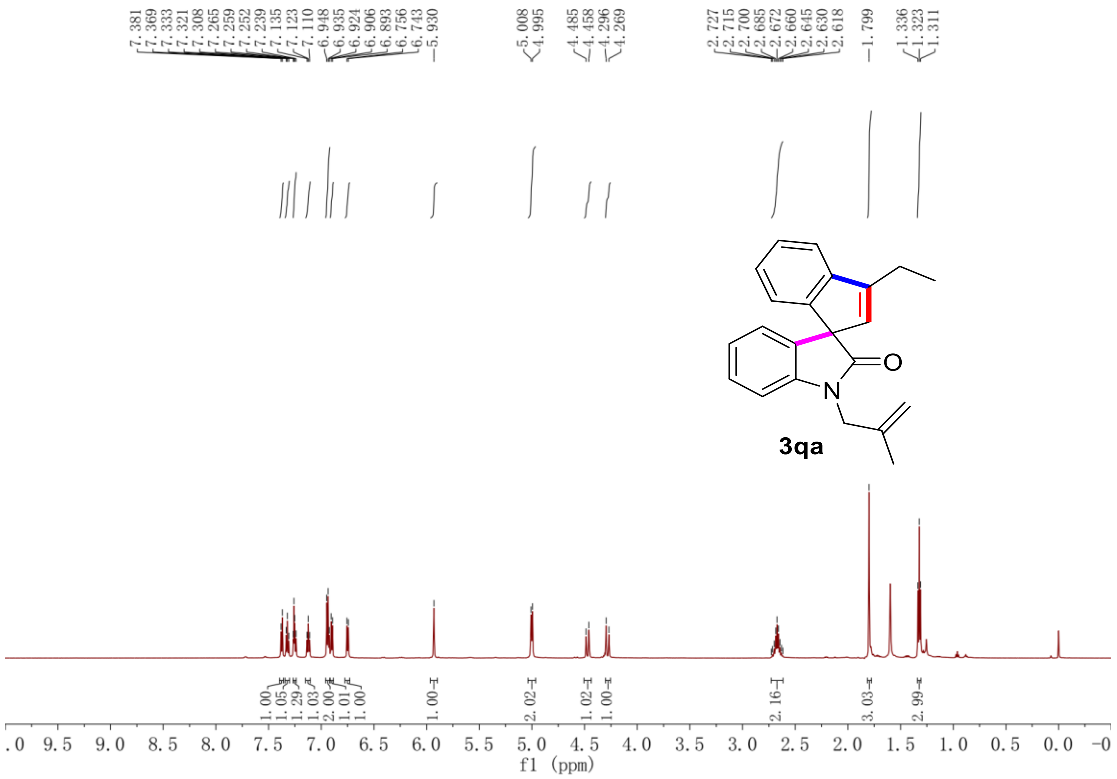

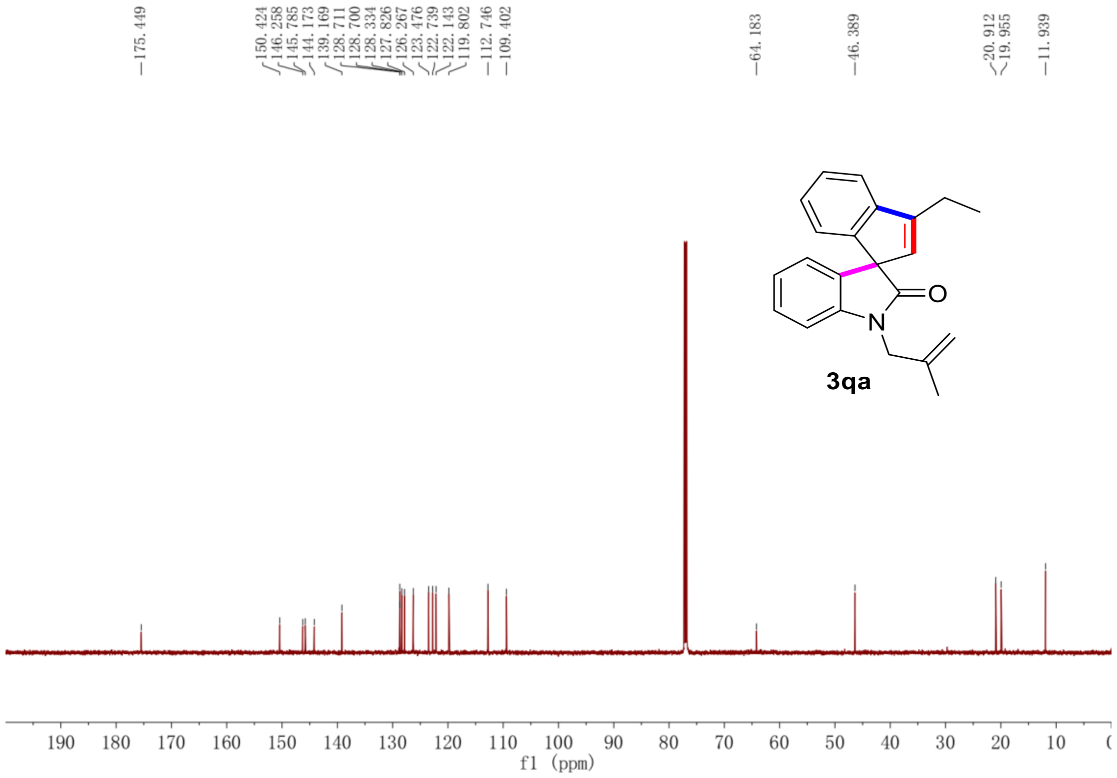

^{1}H NMR of **3ra** (600 MHz, Chloroform-*d*) and ^{13}C NMR of **3ra** (151 MHz, Chloroform-*d*)

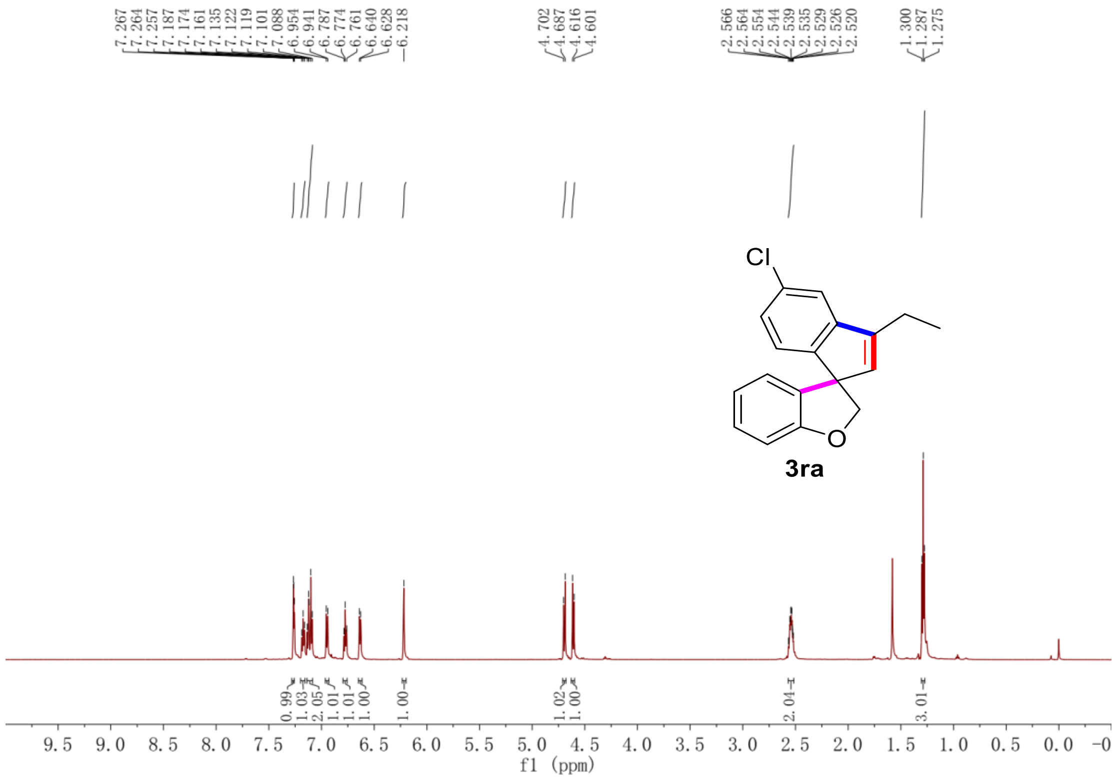

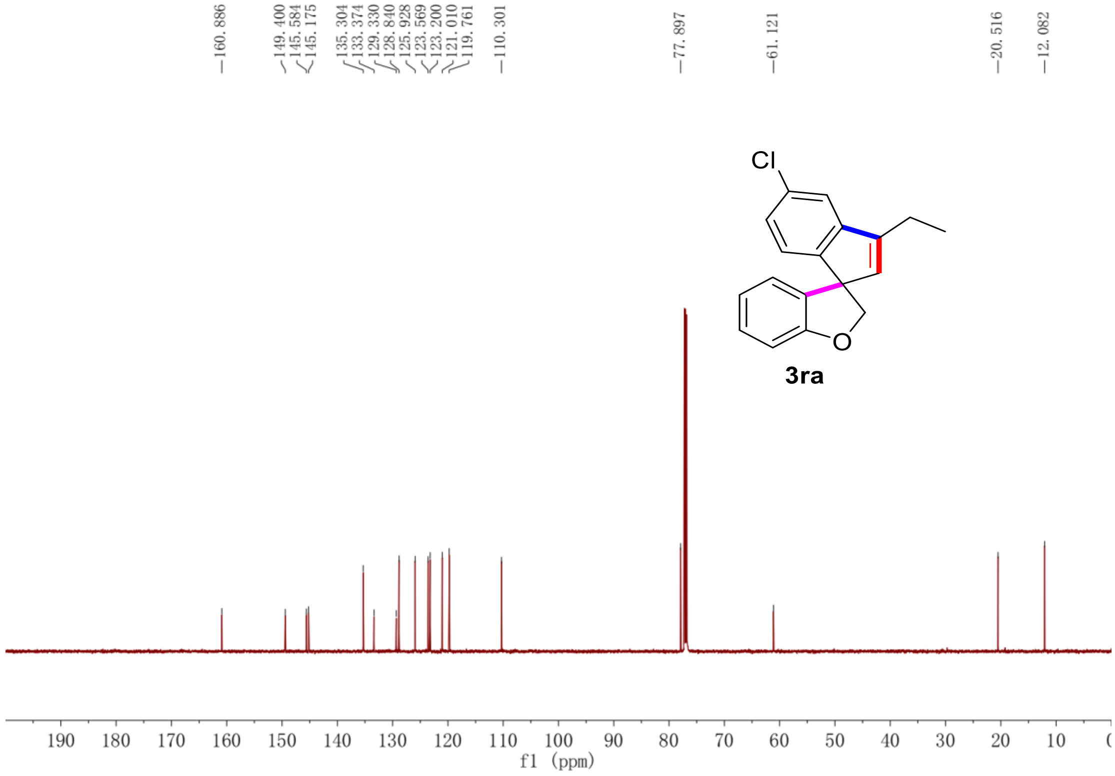

^{1}H NMR of **3ab** (600 MHz, Chloroform-*d*) and ^{13}C NMR of **3ab** (101 MHz, Chloroform-*d*)

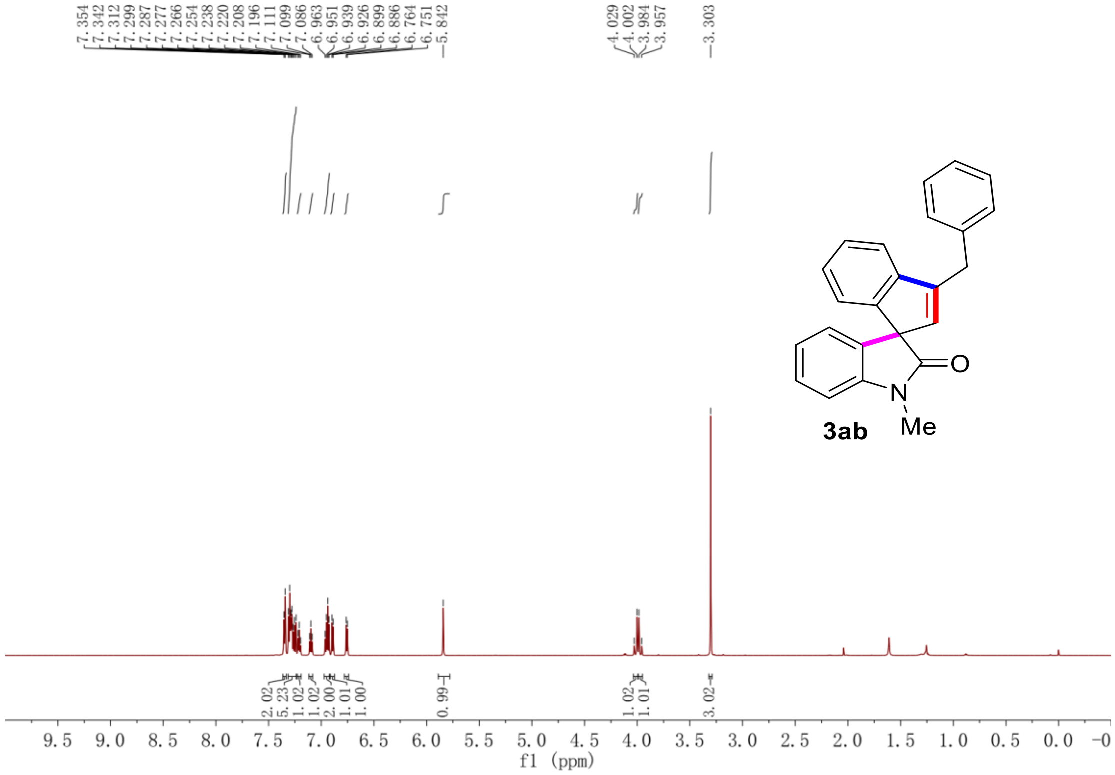

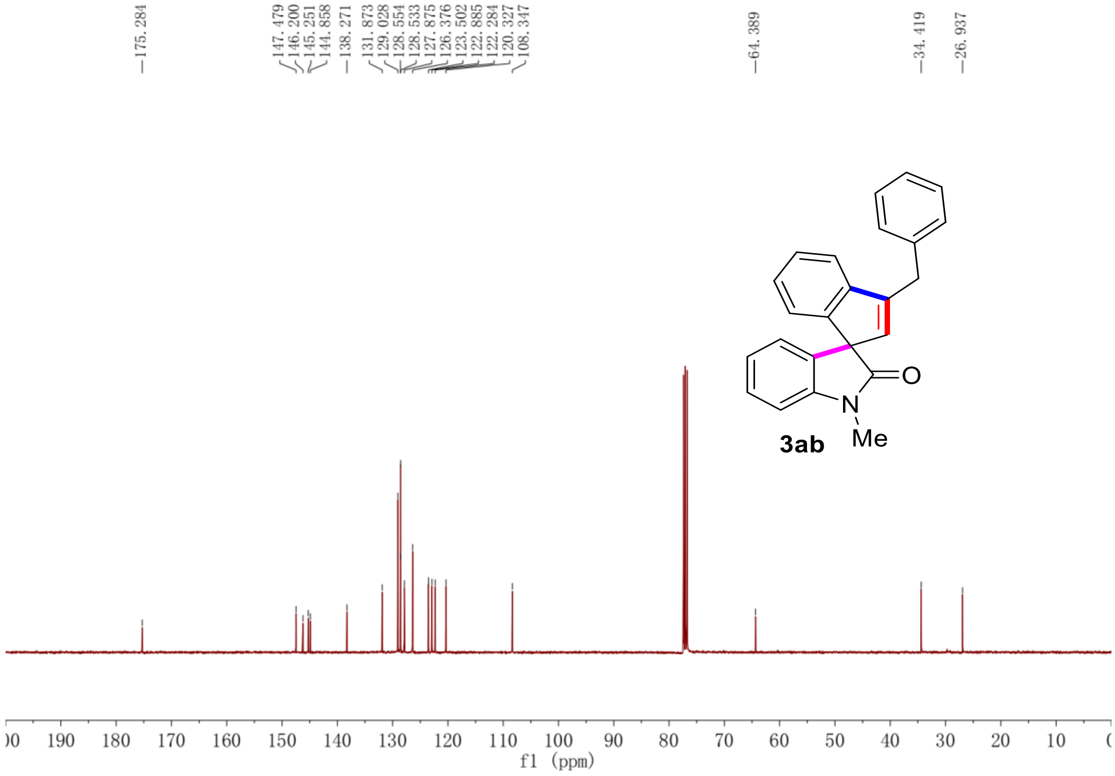

^{1}H NMR of **3ab-l** (400 MHz, Chloroform-*d*) and ^{13}C NMR of **3ab-l** (101 MHz, Chloroform-*d*)

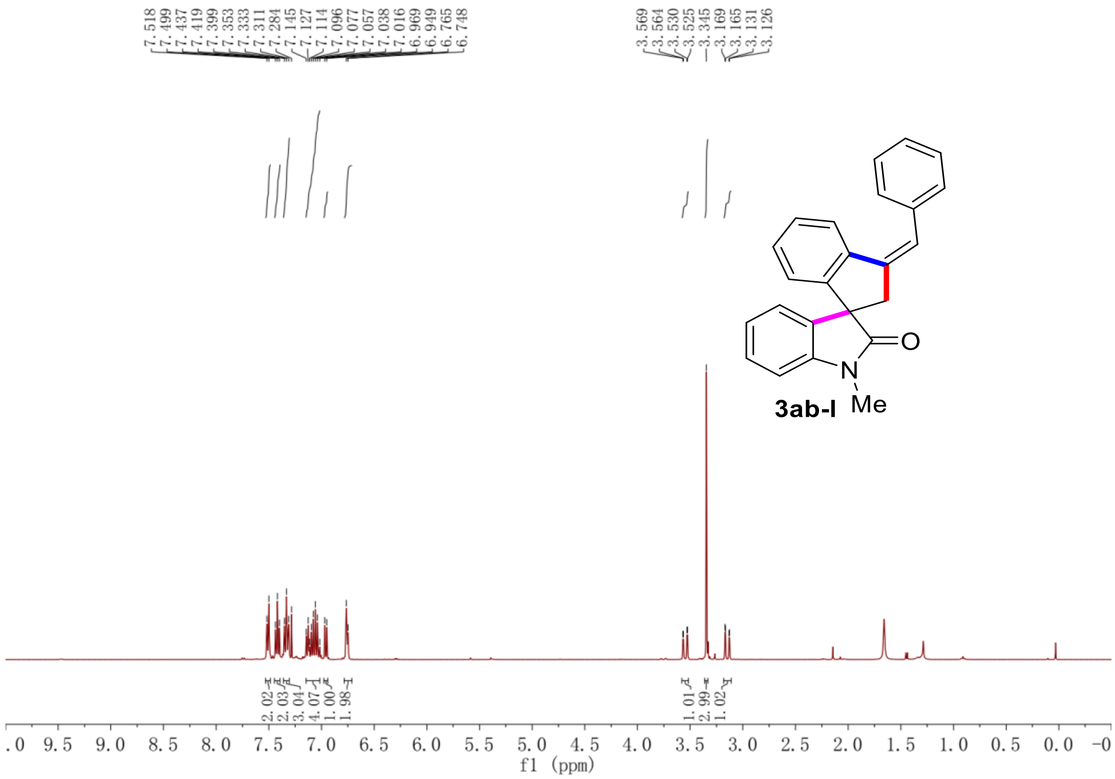

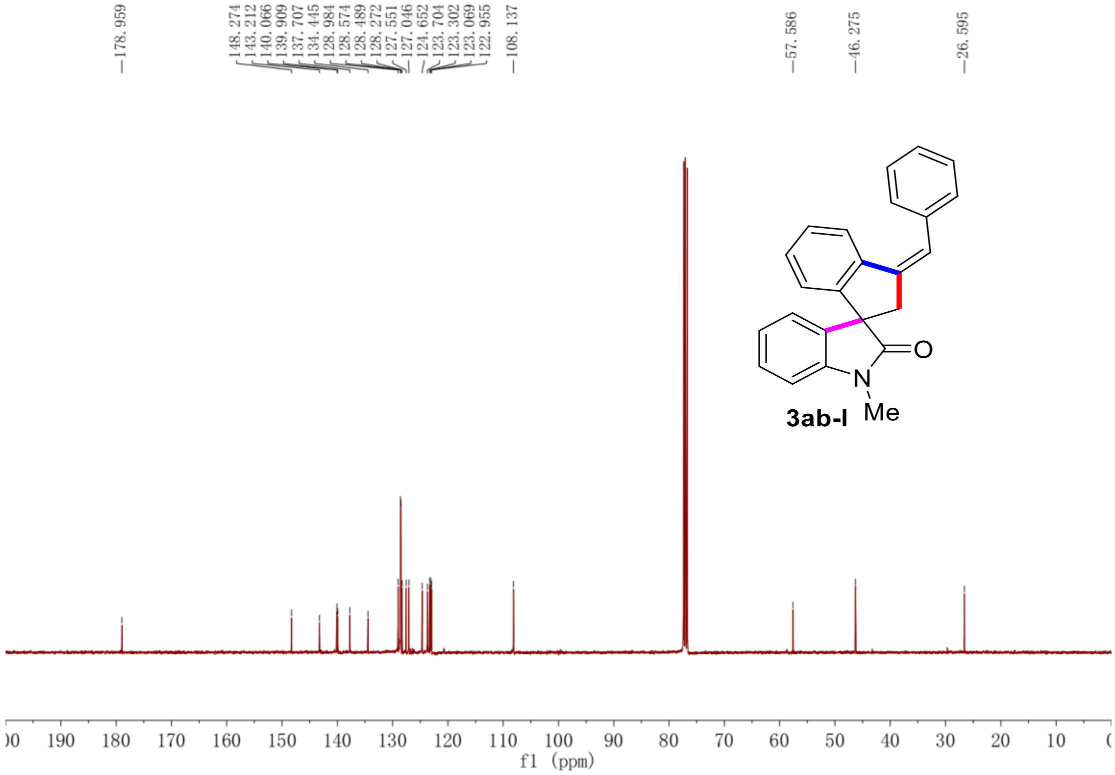

^{1}H NMR of **3ac** (400 MHz, Chloroform-*d*) and ^{13}C NMR of **3ac** (101 MHz, Chloroform-*d*)

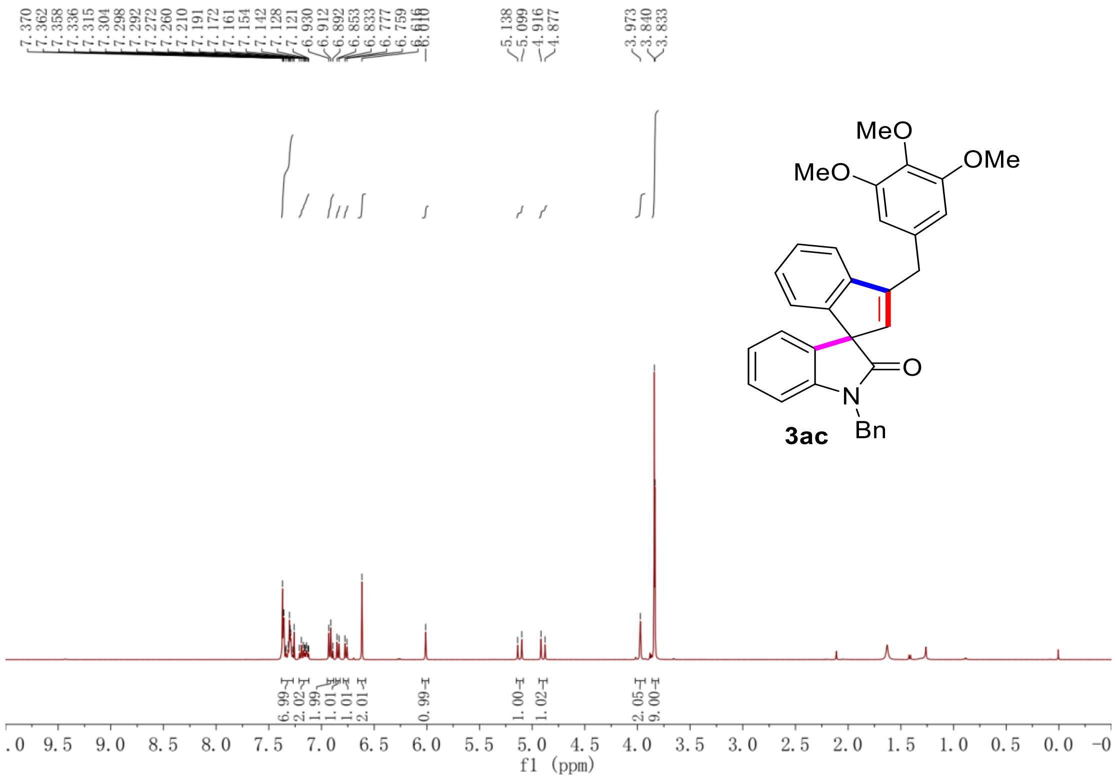

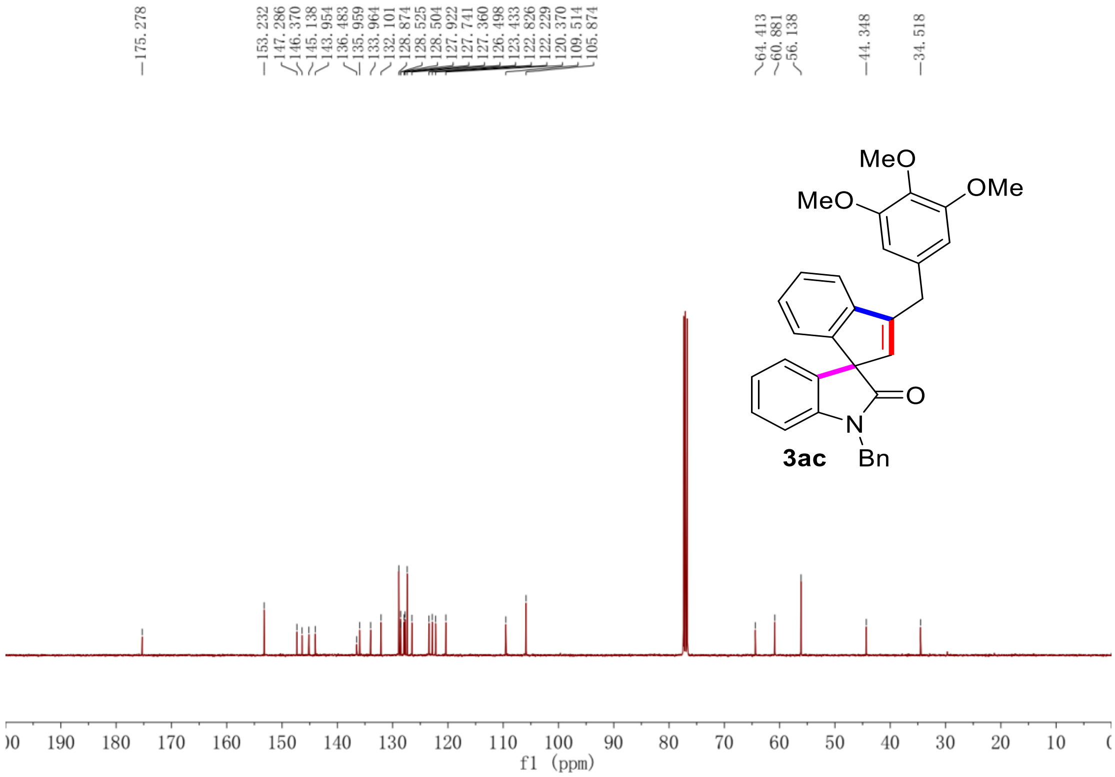

^{1}H NMR of **3ac-l** (600 MHz, Chloroform-*d*) and ^{13}C NMR of **3ac-lb** (151 MHz, Chloroform-*d*)

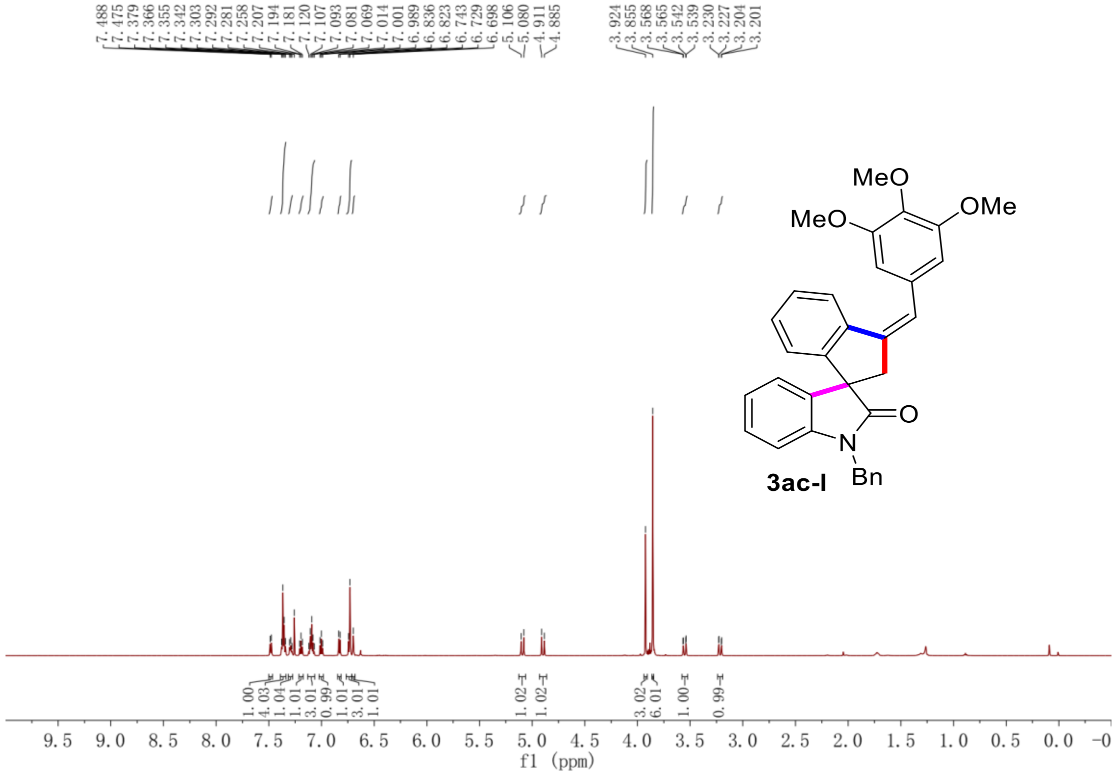

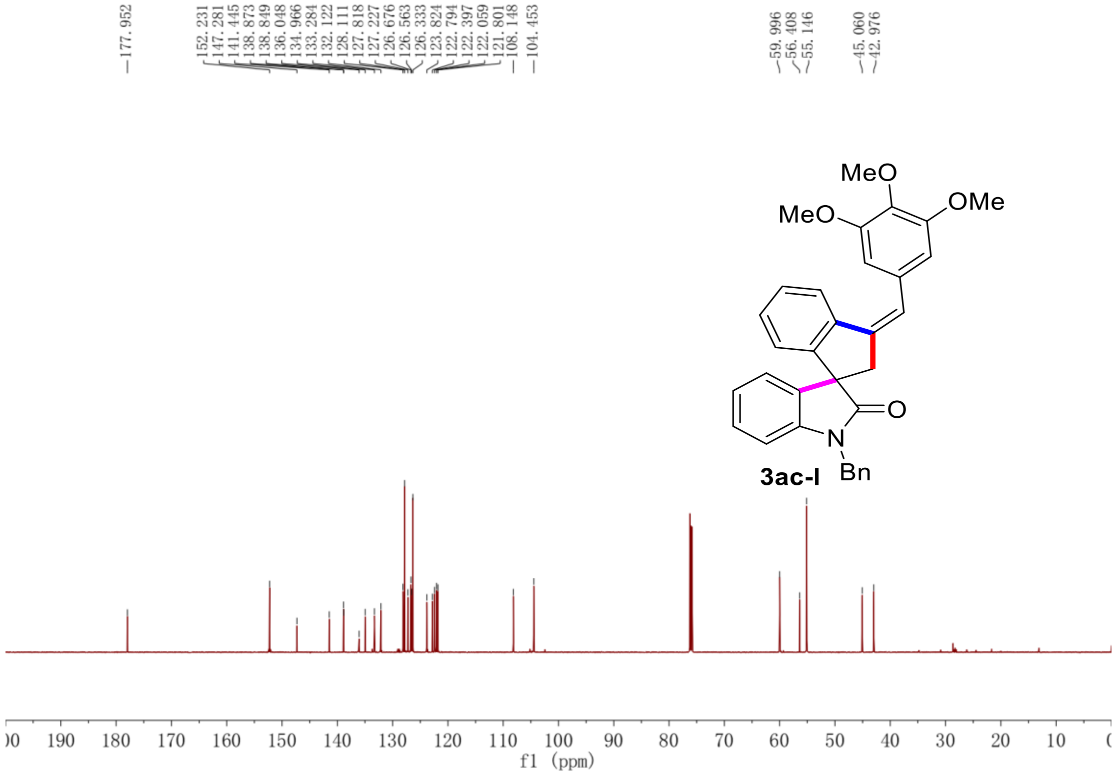

^{1}H NMR of **3ad** (400 MHz, Chloroform-*d*) and ^{13}C NMR of **3ad** (101 MHz, Chloroform-*d*)

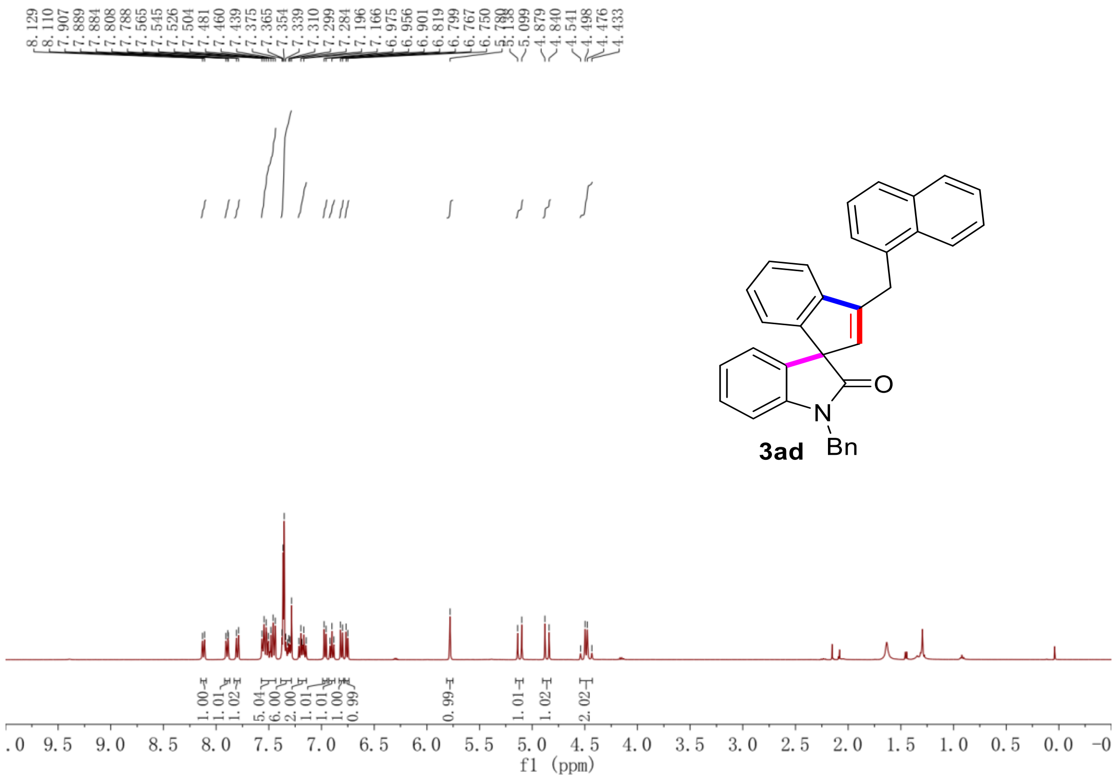

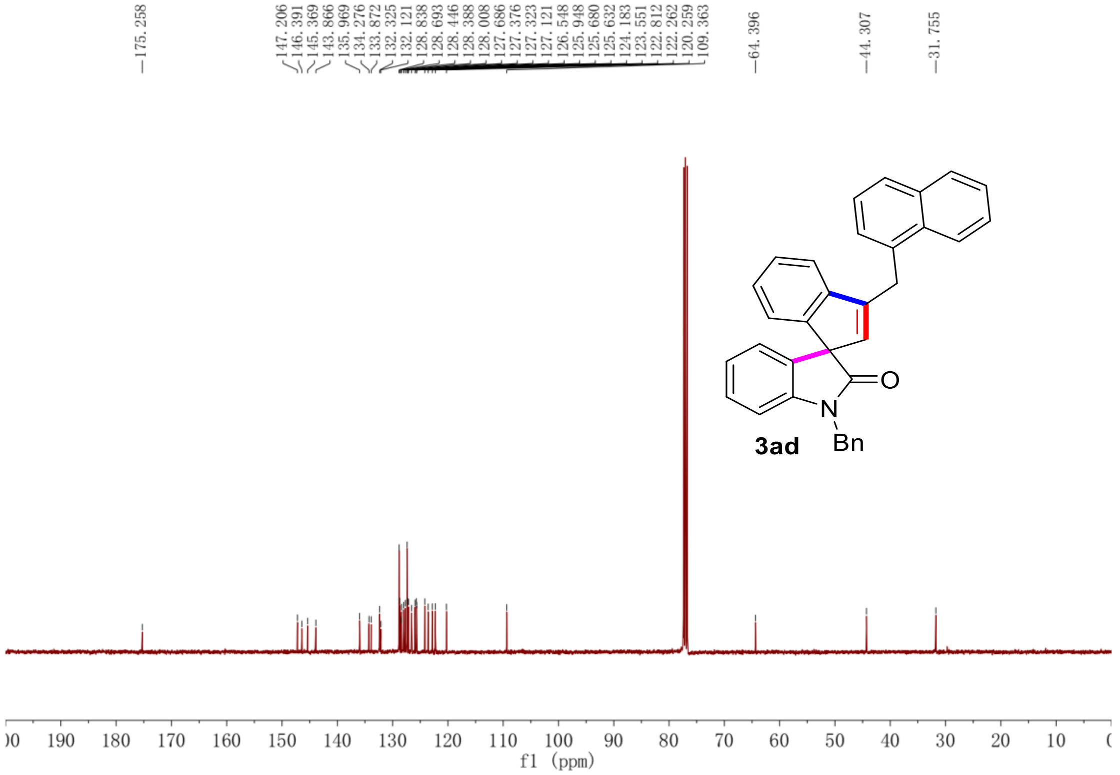

^{1}H NMR of **3ad-l** (600 MHz, Chloroform-*d*) and ^{13}C NMR of **3ad-l** (151 MHz, Chloroform-*d*)

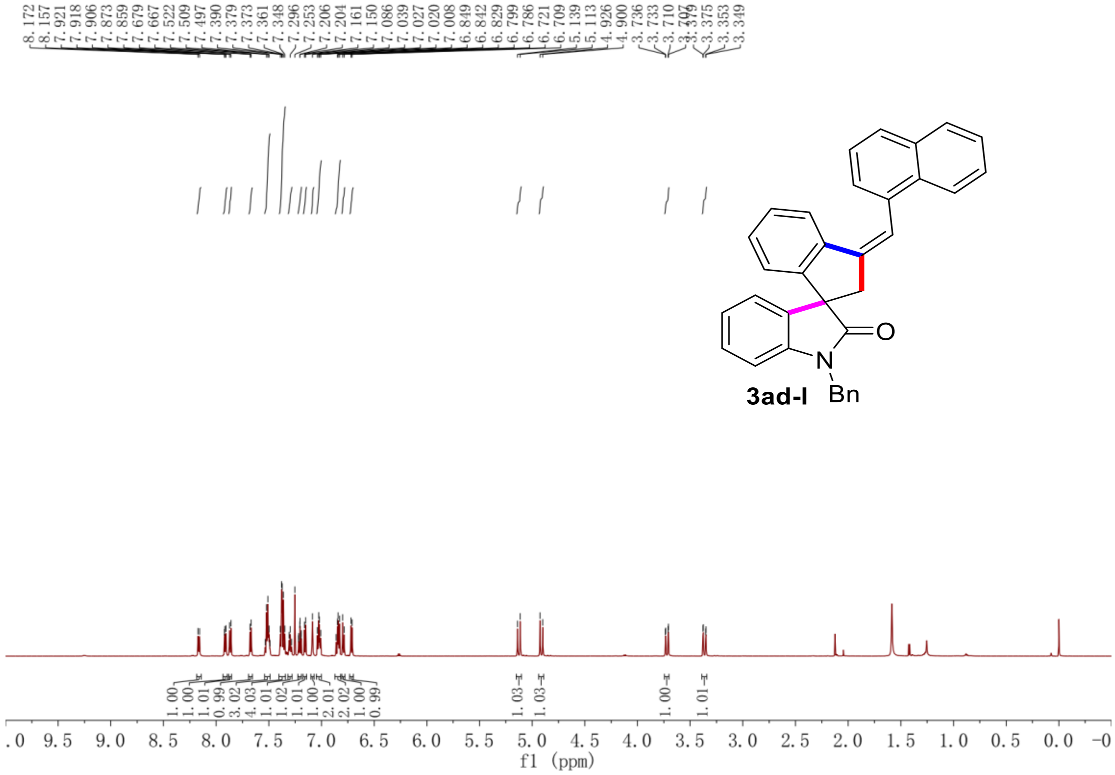

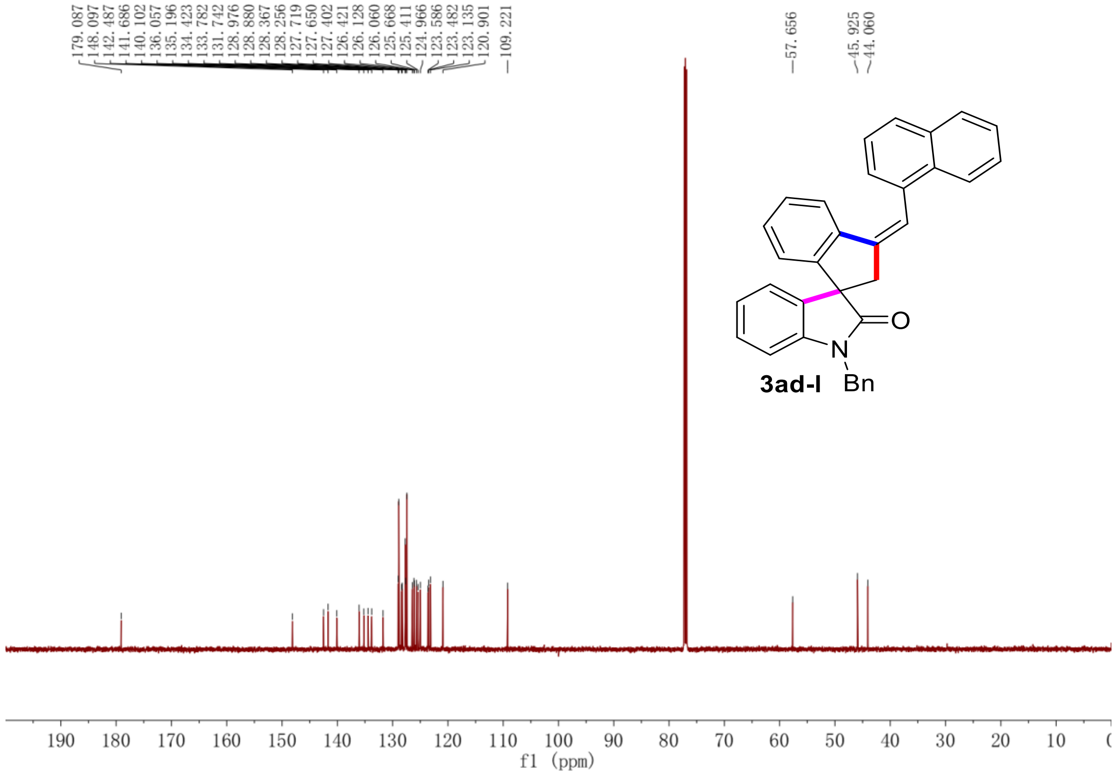

^{1}H NMR of **3ae-l** (600 MHz, Chloroform-*d*) and ^{13}C NMR of **3ae-l** (151 MHz, Chloroform-*d*)

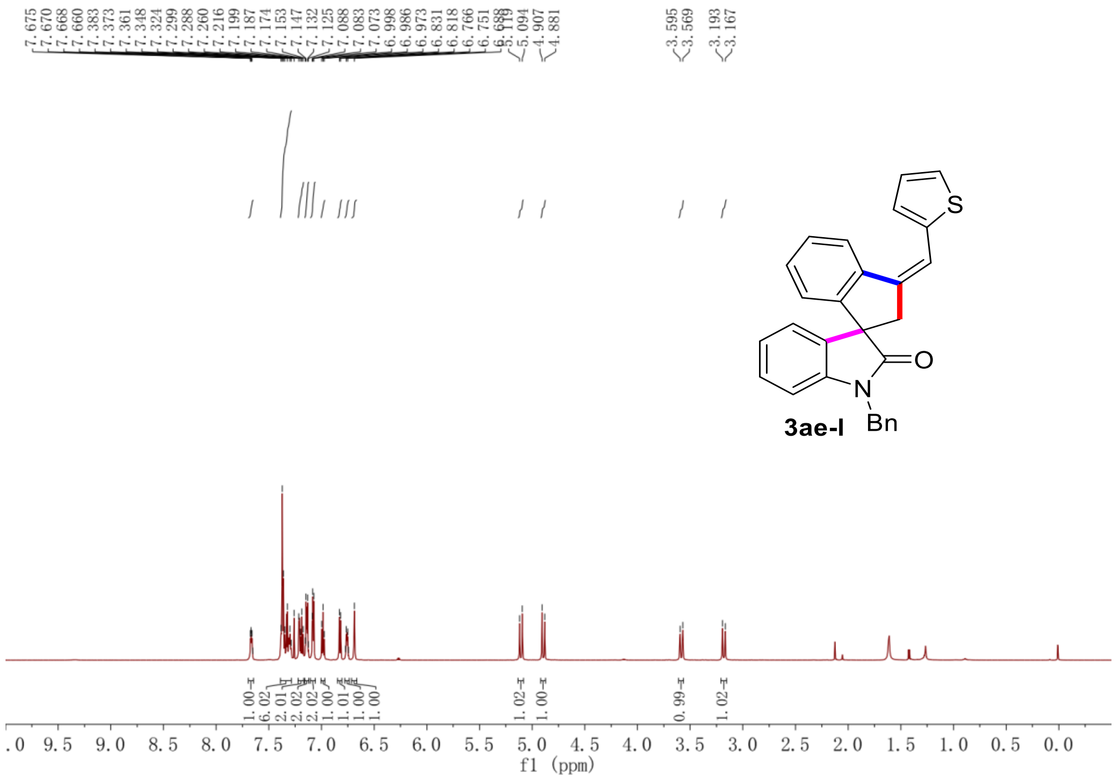

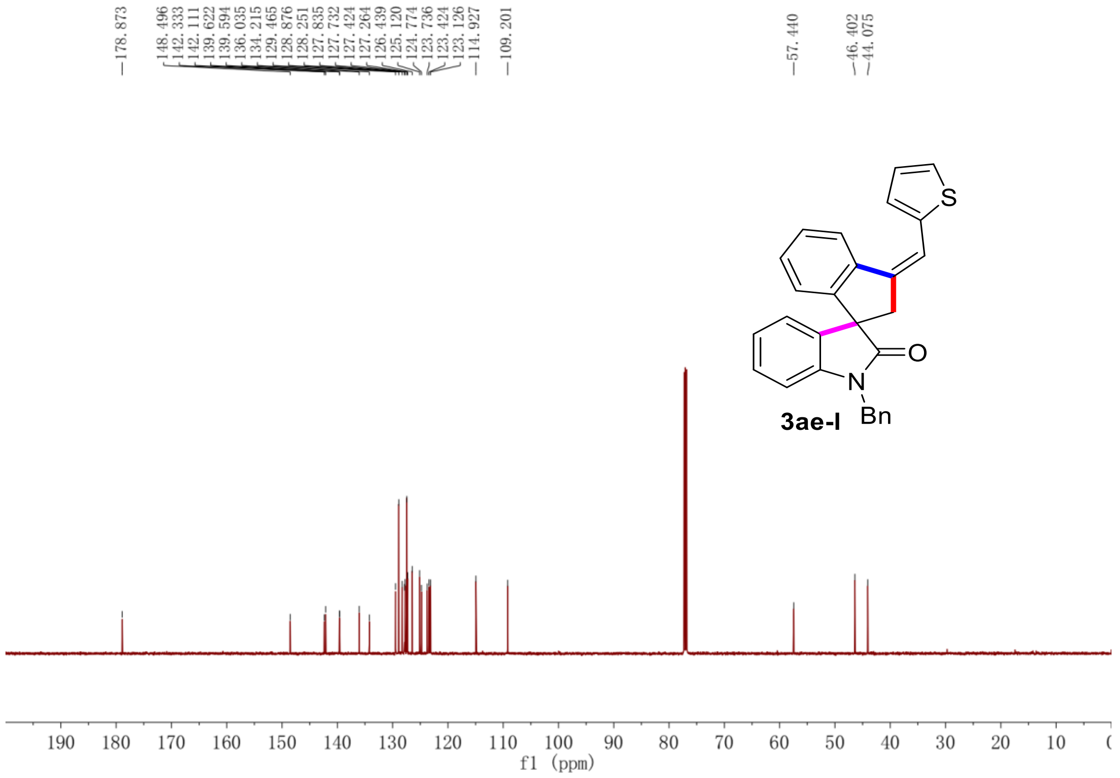

^{1}H NMR of **3af** (600 MHz, Chloroform-*d*) and ^{13}C NMR of **3af** (151 MHz, Chloroform-*d*)

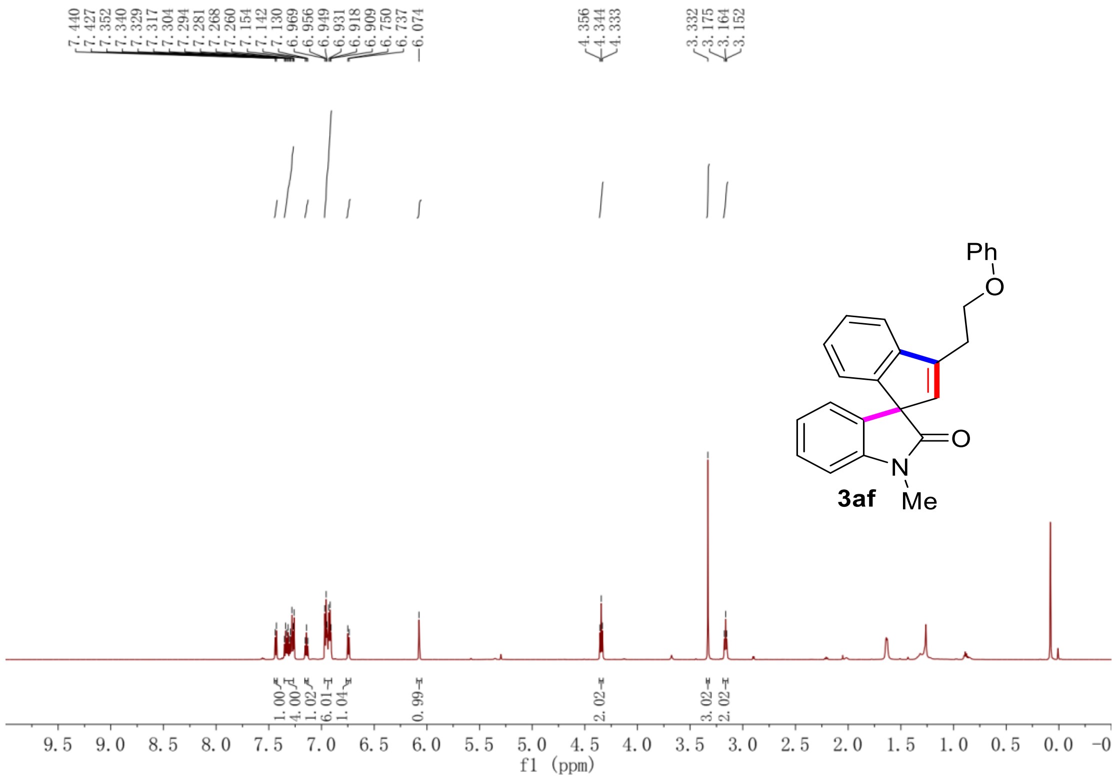

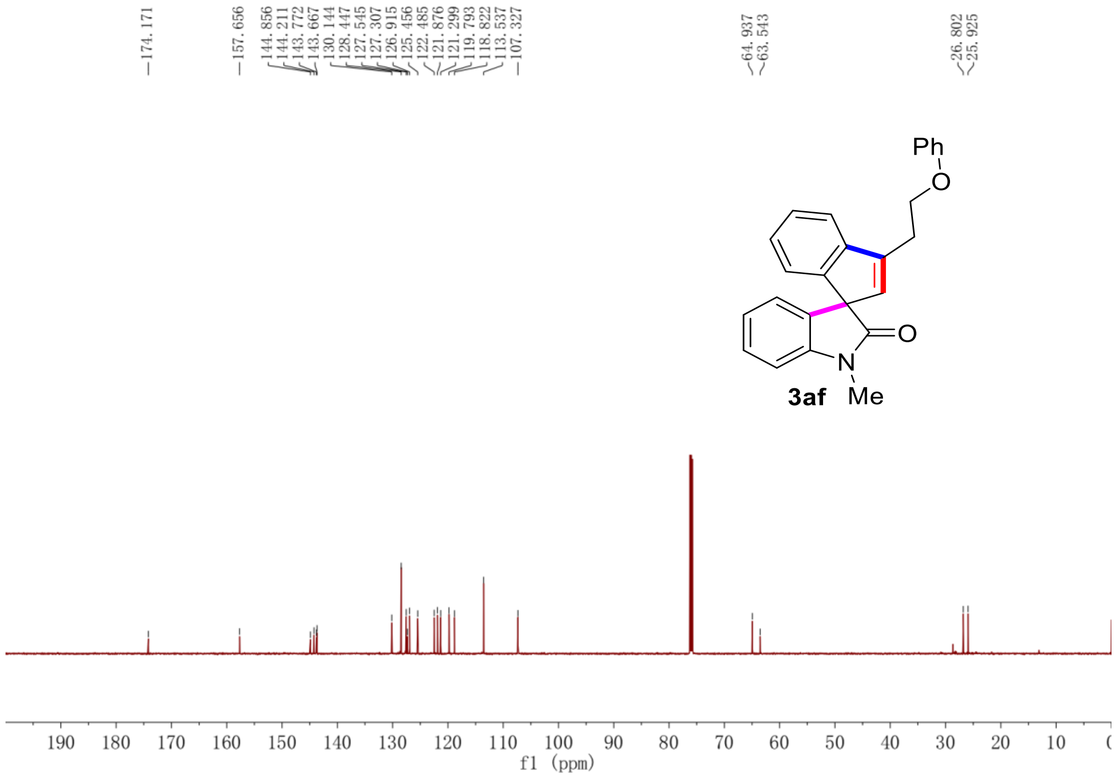

^{1}H NMR of **3af-l** (400 MHz, Chloroform-*d*) and ^{13}C NMR of **3af-l** (101 MHz, Chloroform-*d*)

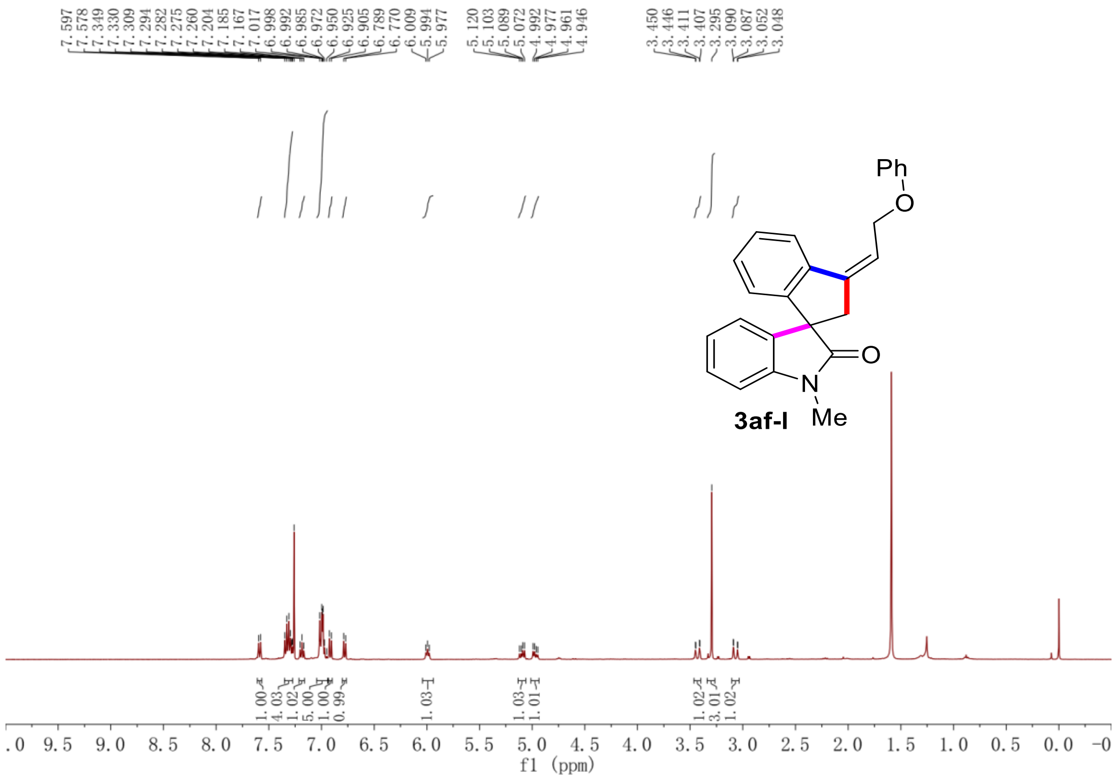

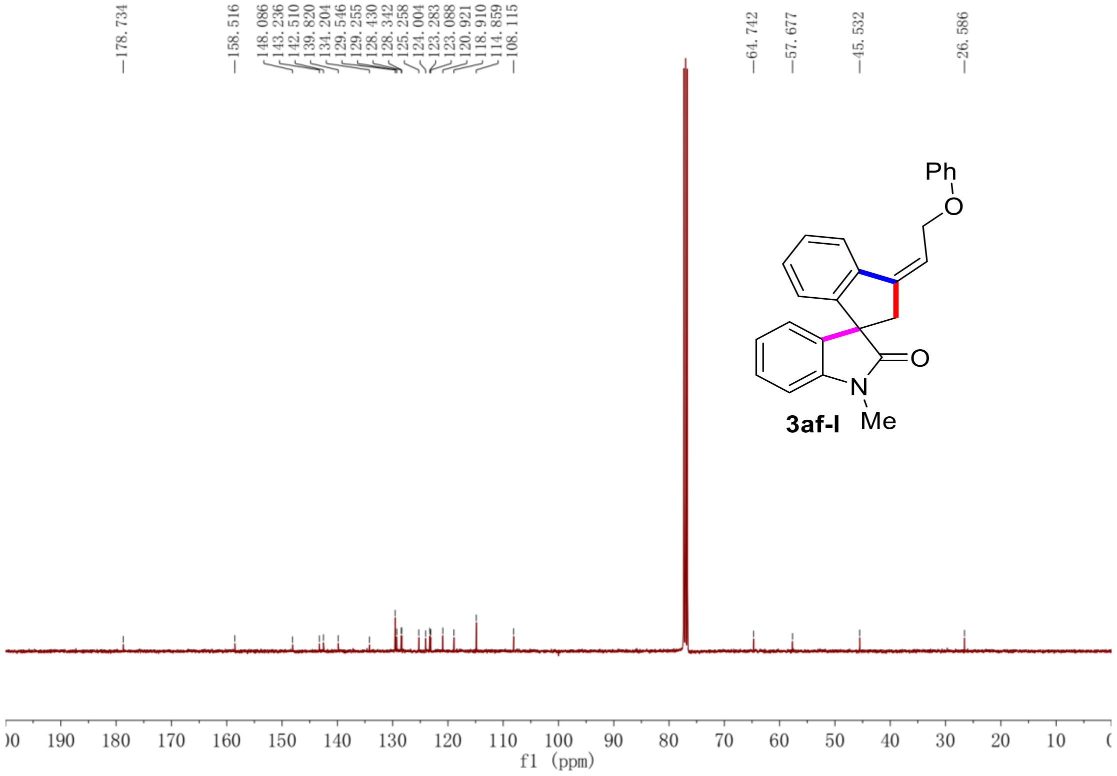

6. X-Ray crystal structure of 3ja (CCDC: 2119724).

General procedure for crystal culture of **3ja**: To a test tube (15 mL) with added **3ja** (41.1 mg), dichloromethane (1.0 mL) was added slowly to make it dissolve completely. After it dissolved, a mixture of petroleum ether (2.0 mL) was added. Then, the test tube was sealed with a rubber stopper, and connected to air with a syringe needle. Finally, the tube was put in a dry and ventilated place to make the organic solvent to volatilize slowly. After a few days, the crystal of **3ja** was obtained. The X-ray crystal structure of **3ja** was shown in Figure **S1**.

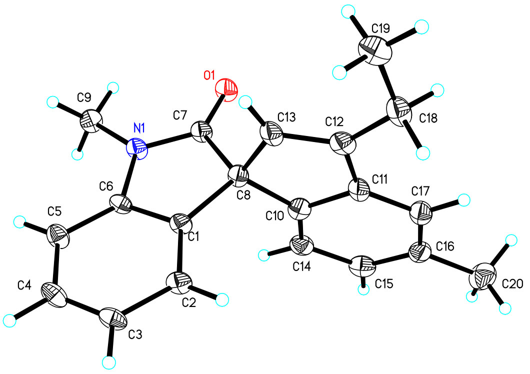

Figure S1 ORTEP diagram of **3ja** with thermal displacement parameters drawn at 30% probability

210913c_a

Table 1 Crystal data and structure refinement for 210913c_a.

Identification code	210913c_a
Empirical formula	$C_{20}H_{19}NO$
Formula weight	289.36
Temperature/K	140.0
Crystal system	orthorhombic
Space group	$P2_12_12_1$
a/Å	9.456(2)
b/Å	10.771(2)
c/Å	15.383(4)
α/°	90
β/°	90
γ/°	90
Volume/Å^3	1566.8(6)
Z	4
ρ_{calc}g/cm^3	1.227
μ/mm^{-1}	0.075
F(000)	616.0
Crystal size/mm^3	0.26 × 0.17 × 0.16
Radiation	MoKα (λ = 0.71073)
2Θ range for data collection/°	5.056 to 50.196
Index ranges	-11 ≤ h ≤ 11, -12 ≤ k ≤ 12, -18 ≤ l ≤ 18
Reflections collected	18279
Independent reflections	2782 [R_{int} = 0.1285, R_{sigma} = 0.0553]
Data/restraints/parameters	2782/0/203
Goodness-of-fit on F^2	1.119
Final R indexes [I>=2σ (I)]	R_1 = 0.0772, wR_2 = 0.1994
Final R indexes [all data]	R_1 = 0.0831, wR_2 = 0.2063
Largest diff. peak/hole / e Å^{-3}	0.60/-0.33
Flack parameter	0.1(10)

7. X-Ray crystal structure of 3ac-l (CCDC: 2119735).

General procedure for crystal culture of **3ac-l**: To a test tube (15 mL) with added **3ac-l** (51.4 mg), dichloromethane (1.0 mL) was added slowly to make it dissolve completely. After it dissolved, a mixture of petroleum ether (2.0 mL) was added. Then, the test tube was sealed with a rubber stopper, and connected to air with a syringe needle. Finally, the tube was put in a dry and ventilated place to make the organic solvent to volatilize slowly. After a few days, the crystal of **3ac-l** was obtained. The X-ray crystal structure of **3ac-l** was shown in Figure **S2**.

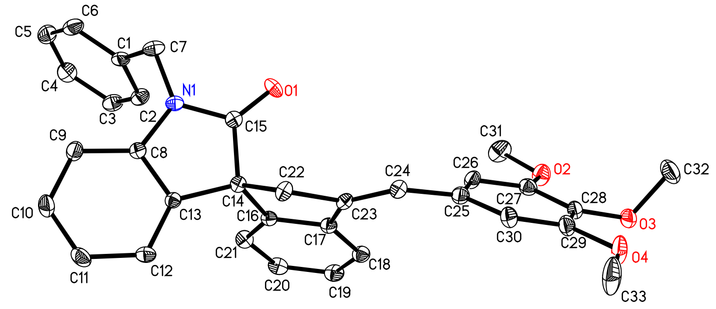

Figure S2 ORTEP diagram of **3ac-l** with thermal displacement parameters drawn at 30% probability

mo_211011a_0m_a

Table 1 Crystal data and structure refinement for mo_211011a_0m_a.

Identification code	mo_211011a_0m_a
Empirical formula	$C_{66}H_{60}N_2O_9$
Formula weight	1025.16
Temperature/K	144.00
Crystal system	monoclinic
Space group	$P2_1/n$
a/Å	11.1256(11)
b/Å	14.162(2)
c/Å	33.332(5)
α/°	90
β/°	93.887(5)
γ/°	90
Volume/Å^3	5239.7(13)
Z	4
ρ_{calc}g/cm^3	1.300
μ/mm^{-1}	0.086
F(000)	2168.0
Crystal size/mm^3	0.3 × 0.2 × 0.12
Radiation	MoKα (λ = 0.71073)
2Θ range for data collection/°	4.272 to 50.76
Index ranges	-13 ≤ h ≤ 12, -17 ≤ k ≤ 17, -40 ≤ l ≤ 40
Reflections collected	83667
Independent reflections	9654 [R_{int} = 0.0491, R_{sigma} = 0.0270]
Data/restraints/parameters	9654/0/705
Goodness-of-fit on F^2	1.054
Final R indexes [I>=2σ (I)]	R_1 = 0.0758, wR_2 = 0.2147
Final R indexes [all data]	R_1 = 0.0830, wR_2 = 0.2204
Largest diff. peak/hole / e Å^{-3}	0.40/-0.29

8. X-Ray crystal structure of 3af (CCDC: 2119736).

General procedure for crystal culture of **3af**: To a test tube (15 mL) with added **3af** (24.3 mg), dichloromethane (1.0 mL) was added slowly to make it dissolve completely. After it dissolved, a

mixture of petroleum ether (2.0 mL) was added. Then, the test tube was sealed with a rubber stopper, and connected to air with a syringe needle. Finally, the tube was put in a dry and ventilated place to make the organic solvent to volatilize slowly. After a few days, the crystal of **3af** was obtained. The X-ray crystal structure of **3af** was shown in Figure **S3**.

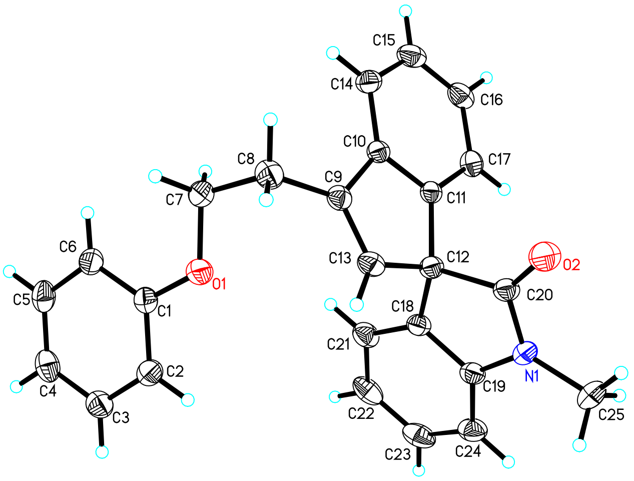

Figure S3 ORTEP diagram of **3af** with thermal displacement parameters drawn at 30% probability

mo_211025a_a

Table 1 Crystal data and structure refinement for mo_211025a_a.

Identification code	mo_211025a_a
Empirical formula	$C_{25}H_{21}NO_2$
Formula weight	367.43
Temperature/K	275.0
Crystal system	orthorhombic
Space group	$P2_12_12_1$
a/Å	7.976(2)
b/Å	11.146(3)
c/Å	21.390(5)
α/°	90
β/°	90
γ/°	90
Volume/Å^3	1901.5(8)
Z	4
ρ_{calc}g/cm^3	1.283
μ/mm^{-1}	0.081
F(000)	776.0
Crystal size/mm^3	0.29 × 0.16 × 0.10
Radiation	MoKα (λ = 0.71073)
2Θ range for data collection/°	3.808 to 50.768
Index ranges	-9 ≤ h ≤ 9, -13 ≤ k ≤ 13, -25 ≤ l ≤ 25
Reflections collected	29632
Independent reflections	3504 [R_{int} = 0.0513, R_{sigma} = 0.0256]
Data/restraints/parameters	3504/0/255
Goodness-of-fit on F^2	1.128
Final R indexes [I>=2σ (I)]	R_1 = 0.0339, wR_2 = 0.0804
Final R indexes [all data]	R_1 = 0.0432, wR_2 = 0.1006
Largest diff. peak/hole / e Å^{-3}	0.16/-0.15
Flack parameter	-0.8(6)
